# Synthesis, Selected Transformations, and Biological Activity of Alkoxy Analogues of Lepidilines A and C

**DOI:** 10.3390/ma13184190

**Published:** 2020-09-21

**Authors:** Grzegorz Mlostoń, Małgorzata Celeda, Wiktor Poper, Mateusz Kowalczyk, Katarzyna Gach-Janczak, Anna Janecka, Marcin Jasiński

**Affiliations:** 1 Department of Organic and Applied Chemistry, Faculty of Chemistry, University of Łódź, Tamka 12, 91403 Łódź, Poland; malgorzata.celeda@chemia.uni.lodz.pl (M.C.); wk.poper@gmail.com (W.P.); mateusz.kowalczyk4@unilodz.eu (M.K.); 2 The Bio-Med-Chem Doctoral School of the University of Lodz and Lodz Institutes of the Polish Academy of Sciences, Faculty of Biology and Environmental Protection, University of Łódź, Banacha 12/16, 90237 Łódź, Poland; 3 Department of Biomolecular Chemistry, Medical University of Łódź, Mazowiecka 6/8, 92215 Łódź, Poland; katarzyna.gach@umed.lodz.pl (K.G.-J.); anna.janecka@umed.lodz.pl (A.J.)

**Keywords:** imidazolium salts, lepidiline alkaloids, imidazole *N*-oxides, *N*-heterocyclic carbenes, sulfur-transfer reaction, anticancer activity

## Abstract

Condensation of diacetyl monooxime with formaldimines derived from alkoxyamines in glacial acetic acid at room temperature leads to corresponding 2-unsubstituted imidazole *N*-oxides bearing an alkoxy substituent at the N(1) atom of the imidazole ring. Subsequent *O*-benzylation afforded, depending on the type of alkylating agent, either symmetric or nonsymmetric alkoxyimidazolium salts considered as structural analogues of naturally occurring imidazole alkaloids, lepidilines A and C. Some of the obtained salts were tested as precursors of nucleophilic heterocyclic carbenes (NHCs), which in situ reacted with elemental sulfur to give the corresponding *N*-alkoxyimidazole-2-thiones. The cytotoxic activity of selected 4,5-dimethylimidazolium salts bearing either two benzyloxy or benzyloxy and 1-adamantyloxy groups at N(1) and N(3) atoms was evaluated against HL-60 and MCF-7 cell lines using the MTT (3-(4, 5-dimethylthiazol-2-yl)-2, 5-diphenyltetrazolium bromide) assay. Notably, in two cases of alkoxyimidazolium salts, no effect of the counterion exchange (Br^−^ → PF_6_^−^) on the biological activity was observed.

## 1. Introduction

Imidazolium salts constitute an important class of imidazole derivatives with diverse applications in modern organic synthesis and related disciplines. They are known as the core structure of many ionic liquids [1,2,3], which are widely applied as highly polar reaction media recommended as reusable “green solvents”, explored not only in academic laboratories but also in industrial processes. Another relevant field for applications of imidazolium salts relates to generation of nucleophilic heterocyclic carbenes (NHCs) [4,5,6]. Due to the milestone achievements by Arduengo, who isolated the first stable 1,3-diadamantylimidazol-2-ylidene [7,8], they changed from laboratory curiosities to powerful tools of current organic synthesis. Finally, imidazolium salts have extensively been studied as biologically active compounds which display antitumor, antimicrobial, antifungal, and antioxidant activities, among others [9,10,11].

**Figure 1:**
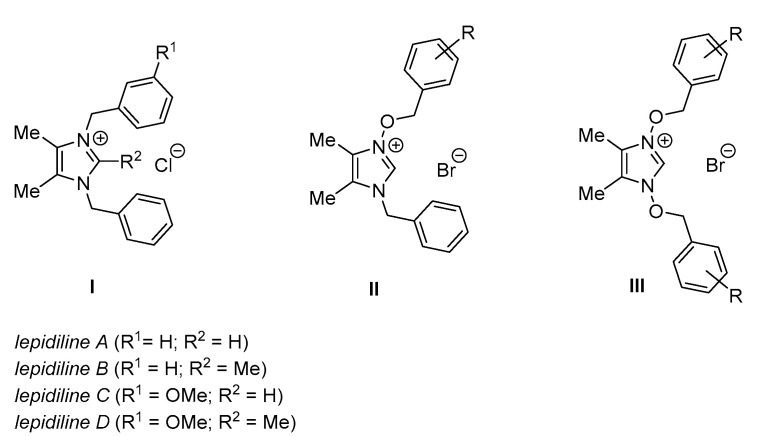
The structure of naturally occurring 4,5-dimethylimidazolium salts **I** (i.e., lepidiline alkaloids) and their 2-unsubstituted mono-alkoxy and bis-alkoxy analogues of type **II** and **III** studied in this work.

A remarkably interesting class of naturally occurring imidazolium alkaloids constitutes lepidilines A–D (**I**, Figure 1) isolated from *Lepidium meyenii* Walpers (so-called Peruvian maca), a South American plant, which is used as a food additive and folk medicine in this region [12,13,14].

The most characteristic feature of the lepidiline structure is the 4,5-dimethylimidazolium ring functionalized at both N atoms with benzyl residues. In the case of lepidilines C and D, the latter subunit contains a methoxy substituent located at the *meta* position. In addition, lepidilines B and D possess another methyl group attached to the C(2) atom of the imidazole ring, as depicted in Figure 1. All isolated compounds in this series were identified as imidazolium chlorides, and the structure of representative molecule of lepidiline A was unambiguously confirmed by X-ray analysis [12]. In the same work anticancer properties of lepidilines A and B were tested against a series of human cancer cell lines. For example, both compounds exhibit some activity toward the FDIGROV cell line but the latter molecule was slightly more active and showed promising activity also against the UMUC3, PACA2, and MDA231 lines. In addition to the protocols for the isolation of **I** from natural sources, the syntheses of lepidilines A and B via double one-pot *N*-benzylation of the respective parent heterocycle were also reported [15,16].

In more recent publications by our group, straightforward protocols for the synthesis of alkoxy-functionalized imidazolium salts, as well as their applications for generation of the corresponding *N*-alkoxyimidazol-2-ylidenes, were demonstrated [17,18]. In these studies, the respective 2-unsubstituted imidazole *N*-oxides served as convenient substrates. Upon treatment with alkyl bromides, they provided desired imidazolium salts in high yields and purity. On the basis of earlier findings, we envisioned possible application of the developed protocols for the preparation of hitherto unknown alkoxy analogues of lepidilines A and C. Hence, the main goal of the present work was the synthesis, detailed spectroscopic analysis, and initial cytotoxicity screening of a series of *N*-benzyloxy (**II**) and *N*,*N*′-bis-benzyloxy (**III**) imidazolium salts. Furthermore, application of the title imidazolium salts as NHC precursors for sulfur-transfer reactions leading to little-known alkoxy-substituted, non-enolisable imidazole-2-thiones should also be checked.

## 2. Materials and Methods

### 2.1. Synthesis

*General information*. All commercially available solvents and reagents were used as received. If not stated otherwise, reactions were performed in flame-dried flasks under the atmosphere of inert gas with addition of the reactants using a syringe; subsequent manipulation was conducted in air. NMR spectra were taken with Bruker AVIII (^1^H-NMR (600 MHz); ^13^C-NMR (151 MHz)). Chemical shifts are given relative to solvent residual peaks; integrals in accordance with assignments and coupling constants *J* are given in Hz. For detailed peak assignments, two-dimensional (2D) spectra were measured (COSY, HMQC). Mass spectra were performed with a Varian 500-MS LC Ion Trap or with a Waters Synapt G2-Si mass spectrometers (Milford, MA, USA). Infrared (IR) measurements were performed with an Agilent Cary 630 Fourier-transform IR (FTIR) spectrometer, in neat. Elemental analyses were obtained with a Vario EL III instrument (Elementar Analysensysteme GmbH, Langenselbold, Germany). Melting points were determined in capillaries with an Aldrich Melt-Temp II apparatus and they are uncorrected.

*Starting materials.* The starting formaldimines 1 were prepared by analogy to a previously reported protocol, comprising alkylation of commercially available *N*-hydroxyphtalimide with appropriate alkyl halide and subsequent hydrazine-mediated release (hydrazinolysis) of the alkoxyamine, followed by its condensation with formaldehyde [18].

*Spectroscopic data*: The ^1^H and ^13^C NMR spectra of all new compounds are collected in Supplementary Materials.

#### 2.1.1. Synthesis of Imidazole N-Oxides 7 and 8

*Method A*: To a solution of diacetyl monooxime (**2a**, 505 mg, 5.0 mmol) or benzyl monooxime (**2b**, 1.12 g, 5.0 mmol) in glacial acetic acid (15 mL) was added appropriate formaldimine **1** (5.0 mmol), and the resulting mixture was stirred at room temperature overnight. Then, excess concentrated hydrochloric acid was added (0.2 mL), the solvents were removed under reduced pressure, the resulting was dissolved in methanol (100 mL), excess solid NaHCO_3_ (ca. 5.0 g) was added, and the stirring was continued for ca. 30 min until the evolution of CO_2_ ceased. After the crude organic salt was fully neutralized, the solvent was removed in vacuo and the residue was triturated with dichloromethane (30 mL). The precipitate was filtered off and the solvent was evaporated to give imidazole *N*-oxide **3**, which was either further purified by column chromatography or recrystallization from a diisopropyl ether/dichloromethane mixture. As per the literature, known imidazole *N*-oxides **3a**–**b**,**g**–**i** crude products were washed with a portion of diethyl ether (ca. 30 mL) and used as received. Analytically pure samples were obtained by crystallization from a diisopropyl ether/dichloromethane mixture (slow evaporation at room temperature).

*Method B*: A mixture of equimolar amounts of α-hydroxyiminoketone of type **2** (5.0 mmol) and corresponding formaldimine **1** (5.0 mmol) in EtOH (10 mL) was refluxed for 4 h. The solvent was removed, and the resulting oily material was triturated with several portions of diethyl ether (4 × 15 mL). The resulting crude imidazole *N*-oxides **3** were purified by recrystallization from diisopropyl ether/dichloromethane mixture (slow evaporation at room temperature).

1-Benzyl-4,5-dimethyl-1*H*-imidazole 3-oxide (**3a**): *Method B*: 880 mg (87%). Colorless solid, melting point (m.p.) 200–201 °C (199–201 °C [19]). ^1^H-NMR (CDCl_3_, 600 MHz): δ 2.07, 2.20 (2 s, 3 H each, 2 Me), 5.00 (s, 2 H), 7.08–7.11, 7.31–7.38 (2 m, 2 H, 3 H, Bn), 7.88 (s_br_, 1 H, C(2)H) ppm.

1-Benzyl-4,5-diphenyl-1*H*-imidazole 3-oxide (**3b**): *Method B*: 1.32 g (81%). Colorless solid, m.p. 176–177 °C (176–178 °C [19]). ^1^H-NMR (CDCl_3_, 600 MHz): δ 4.93 (s, 2 H), 7.03–7.05, 7.18–7.42, 7.55–7.58 (3 m, 2 H, 11 H, 2 H, 3 Ph), 7.98 (s, 1 H, C(2)H) ppm.

1-Benzyloxy-4,5-dimethyl-1*H*-imidazole 3-oxide (**3c**): *Method A*: 719 mg (66%); *Method B*: 0%. Crude product was purified by column chromatography (SiO_2_, AcOEt/MeOH 1:1, *R_f_* = 0.5) to give 7d as colorless solid, m.p. 103–105 °C. ^1^H-NMR (CDCl_3_, 600 MHz): δ 1.94, 2.10 (2 s, 3 H each, 2 Me), 5.03 (s, 2 H, Bn), 7.27–7.29, 7.35–7.42 (2 m, 2 H, 3 H, Bn), 7.73 (s_br_, 1 H, C(2)H) ppm. ^13^C-NMR (CDCl_3_, 151 MHz): δ 7.0, 7.2 (2 q, 2 Me), 82.7 (t, Bn), 119.3 (s, Im), 120.6 (d, C(2)), 123.7 (s, Im), 129.0, 129.9, 130.1 (3 d, Bn), 132.4 (s, Bn) ppm. IR (neat): *ν* 3070, 1675, 1457, 1390, 1172, 1079, 941, 908 cm^−1^. Electrospray ionization (ESI)–MS (*m***/***z*): 241.2 (42, [*M* + Na]^+^), 219.3 (100, [*M* + H]^+^). C_12_H_14_N_2_O_2_·0.8 H_2_O: calculated, C 61.95, H 6.76, N 12.04; found, C 61.90, H 6.84, N 12.10.

1-(2-Methylbenzyloxy)-4,5-dimethyl-1*H*-imidazole 3-oxide (**3d**): *Method A*: 709 mg (61%). Colorless solid, m.p. 89–91 °C. ^1^H-NMR (CDCl_3_, 600 MHz): δ 1.91, 2.07, 2.36 (3 s, 3 H each, 3 Me), 5.07 (s, 2 H, CH_2_), 7.05–7.07, 7.11–7.14, 7.19–7.21, 7.25–7.29 (4 m, 1 H each), 7.70 (s, 1 H, C(2)H) ppm. ^13^C-NMR (CDCl_3_, 151 MHz): δ 6.9, 7.1, 18.8 (3 q, 3 Me), 80.9 (t, CH_2_), 119.3 (s, Im), 120.5 (d, C(2)), 123.6 (s, Im), 126.4, 130.4 (2 d, 2 CH), 130.6 (s, *i*-C), 130.8, 131.2 (2 d, 2 CH), 138.0 (s, *i*-C) ppm. IR (neat): *ν* 1444, 1351, 1169, 1079, 922, 744 cm^−1^. ESI-MS (*m***/***z*): 255.1 (88, [*M* + Na]^+^), 233.1 (100, [*M* + H]^+^). C_13_H_16_N_2_O_2_·2H_2_O (268.3): calcdulated, C 58.19, H 7.51, N 10.44; found, C 58.34, H 6.78, N 10.64.

1-(4-Methylbenzyloxy)-4,5-dimethyl-1*H*-imidazole 3-oxide (**3e**): *Method A*: 789 mg (68%). Colorless solid, m.p. 101–102 °C. ^1^H-NMR (CDCl_3_, 600 MHz): δ 1.93, 2.08, 2.32 (3 s, 3 H each, 3 Me), 4.96 (s, 2 H, CH_2_), 7.14 (m_c_, 4 H), 7.65 (s, 1 H, C(2)H) ppm. ^13^C-NMR (CDCl_3_, 151 MHz): δ 6.9, 7.2, 21.2 (3 q, 3 Me), 82.5 (t, CH_2_), 119.2 (s, Im), 120.6 (d, C(2)), 123.5 (s, Im), 129.4 (s, *i*-C), 129.6, 129.9 (2 d, 4 CH), 140.2 (s, *i*-C) ppm. IR (neat): *ν* 1601, 1448, 1318, 1299, 1172, 1170, 922 cm^−1^. ESI-MS (*m*/*z*): 255.0 (46, [*M* + Na]^+^), 233.1 (100, [*M* + H]^+^). C_13_H_16_N_2_O_2_**·**H_2_O: (250.29): calculated, C 62.38, H 7.25, N 11.19; found, C 62.06, H 7.07, N 11.60.

1-(3,5-Dimethylbenzyloxy)-4,5-dimethyl-1*H*-imidazole 3-oxide (**3f**): *Method A*: 1.17 g (95%). Colorless solid, m.p. 96–98 °C. ^1^H-NMR (CDCl_3_, 600 MHz): δ 1.98, 2.10 (2 s, 3 H each, 2 Me), 2.27 (s, 6 H, 2 Me), 4.94 (s, 2 H, CH_2_), 6.88 (s_br_, 2 H), 7.01 (s_br_, 1 H), 7.69 (s, 1 H, C(2)H) ppm. ^13^C-NMR (CDCl_3_, 151 MHz): δ 6.9, 7.2 (2 q, 2 Me), 21.1 (q, 2 Me), 82.9 (t, CH_2_), 119.2 (s, Im), 120.6 (d, C(2)), 123.6 (s, Im), 127.4 (d, 2 CH), 131.6 (d, CH), 132.3 (s, *i*-C), 138.7 (s, 2 *i*-C) ppm. IR (neat): *ν* 1608, 1394, 1172, 1081, 938 cm^−1^. ESI-MS (*m***/***z*): 285.1 (100, [*M* + K]^+^), 269.1 (54, [*M* + Na]^+^), 247.1 (87, [*M* + H]^+^). C_14_H_18_N_2_O_2_·1.3 H_2_O: calculated, C 62.20, H 7.71, N 10.36; found, C 62.14, H 7.53, N 10.30.

1-Adamantyl-4,5-dimethyl-1*H*-imidazole 3-oxide (**3g**): *Method A*: 677 mg (55%). Colorless solid, m.p. 179–180 °C (decomposed) (m.p. 180–182 °C (decomposed) [20]). ^1^H-NMR (CDCl_3_, 600 MHz): δ 1.72, 1.77 (2 d_br_, *J* ≈ 12.5 Hz, 6 H, Ad), 2.13 (m_c_, 6 H, Ad), 2.17 (s, 3 H, Me), 2.24 (m_c_, 3 H, Ad), 2.36 (s, 3 H, Me), 7.89 (s, 1 H, C(2)H) ppm.

1-Adamantyl-4,5-diphenyl-1*H*-imidazole 3-oxide (**3h**): *Method A*: 814 mg (44%). Colorless solid, m.p. 234–239 °C (decomposed) (m.p. 238–241 °C (decomposed) [20]). ^1^H-NMR (CDCl_3_, 600 MHz): δ 1.54, 1.65 (2 d_br_, *J* ≈ 12.2 Hz, 6 H, Ad), 2.05 (m_c_, 6 H, Ad), 2.11 (m_c_, 3 H, Ad), 7.14–7.21, 7.33–7.50 (2 m, 3 H, 7 H, 2 Ph), 8.21 (s, 1 H, C(2)H) ppm.

1-Adamantyloxy-4,5-dimethyl-1*H*-imidazole 3-oxide (**3i**): *Method A*: 968 mg (74%). Pale yellow solid, m.p. 103–106 °C (m.p. 104–106 °C [17]). ^1^H-NMR (CDCl_3_, 600 MHz): δ 1.59, 1.70 (2 d_br_, *J* ≈ 12.3 Hz, 6 H, Ad), 1.86 (m_c_, 6 H, Ad), 2.16, 2.20 (2 s, 3 H each, 2 Me), 2.27 (m_c_, 3 H, Ad), 7.85 (s, 1 H, C(2)H) ppm.

#### 2.1.2. General Procedure for the Synthesis of Imidazolium Bromides 4 and 5 

To a solution of corresponding imidazole *N*-oxide **3** (1.0 mmol) in dry dichloromethane (2.0 mL) was added excess alkyl bromide (5.0 mL), and the resulting mixture was stirred at rt until the starting *N*-oxide was fully consumed (thin-layer chromatography (TLC) monitoring: SiO_2_, EtOAc/MeOH 6:1; typically 24–48 h). After the solvent was removed under reduced pressure, the resulting crude product was triturated with several portions of Et_2_O (4 × 10 mL) in order to remove excess of unconsumed alkylating agent. The product was dried under high vacuum to give the corresponding imidazolium bromides, whose identity was confirmed by NMR spectroscopy. Analytically pure samples of products **4** and **5** were obtained by crystallization from diisopropyl ether/dichloromethane mixture (slow evaporation at room temperature).

1-Benzyl-3-benzyloxy-4,5-dimethylimidazolium bromide (**4a**): 369 mg (99%). Colorless solid, m.p. 148–150 °C. ^1^H-NMR (CDCl_3_, 600 MHz): δ 1.92, 2.07 (2 s, 3 H each, 2 Me), 5.52, 5.57 (2 s, 2 H each, 2 Bn), 7.28–7.42, 7.49–7.52 (2 m, 8 H, 2 H, 2 Bn), 11.00 (s, 1 H, C(2)H) ppm. ^13^C-NMR (CDCl_3_, 151 MHz): δ 7.1, 8.9 (2 q, 2 Me), 51.3 (t, NBn), 84.0 (t, OBn), 124.1, 124.8 (2 s, Im), 128.0, 129.9, 129.0, 129.2, 130.3, 130.6 (6 d, 2 Bn), 131.5 (s, Bn), 132.5 (d_br_, C(2)), 132.9 (s, Bn) ppm. IR (neat): *ν* 2924, 1453, 1340, 1139, 909 cm^−1^. C_19_H_21_N_2_OBr (373.3): calculated, C 61.13, H 5.67, N 7.50; found, C 61.08, H 5.73, N 7.69.

1-Benzyl-3-benzyloxy-4,5-diphenylimidazolium bromide (**4b**): 442 mg (89%). Colorless solid, m.p. 167–169 °C. ^1^H-NMR (CDCl_3_, 600 MHz): δ 5.42 (s, 2 H, Bn), 5.58 (s, 2 H, Bn), 7.11–7.13, 7.18–7.21, 7.25–7.33, 7.39–7.45, 7.51–7.54 (5 m, 2 H, 4 H, 10 H, 3 H, 1 H, 2 Ph, 2 Bn), 11.17 (s, 1 H, C(2)H) ppm. ^13^C-NMR (CDCl_3_, 151 MHz): δ 51.3 (t, NBn), 84.3 (t, OBn), 122.9, 124.2 (2 s, Im), 128.61*, 128.63, 128.73, 128.91, 128.94, 130.5, 130.8, 130.9 (8 d, 20 CH, 2 Ph, 2 Bn), 128.72, 129.3, 129.4, 133.1 (4 s, 2 Ph, 2 Bn), 133.7 (d_br_, C(2)) ppm; *higher intensity. IR (neat): *ν* 2861, 1547, 1456, 1385, 1340, 951, 913 cm^−1^. C_29_H_25_N_2_OBr (497.4): calculated, C 70.02, H 5.07, N 5.63; found, C 69.12, H 5.15, N 5.76.

1-Adamantyl-3-benzyloxy-4,5-dimethylimidazolium bromide (**4c**): 384 mg (92%). Colorless solid, m.p. 196–197 °C. ^1^H-NMR (CDCl_3_, 600 MHz): δ 1.70–1.77 (m, 6 H, Ad), 1.96 (s, 3 H, Me), 2.28 (m_c_, 3 H, Ad), 2.30–2.33 (m, 6 H, Ad), 2.38 (s, 3 H, Me), 5.77 (s, 2 H, Bn), 7.32–7.37, 7.56–7.59 (2 m, 3 H, 2 H, Bn), 10.44 (s, 1 H, C(2)H) ppm. ^13^C-NMR (CDCl_3_, 151 MHz): δ 7.0, 12.4 (2 q, 2 Me), 29.5, 35.2, 41.6 (d, t, t, Ad), 64.0 (s, Ad), 84.0 (t, Bn), 123.1, 126.2 (2 s, Im), 128.7, 129.9, 130.8 (3 d, Bn), 131.8 (d_br_, C(2)), 132.2 (s, Bn) ppm. IR (neat): *ν* 2911, 2853, 1457, 1303, 1224, 1178, 913 cm^−1^. C_22_H_29_N_2_OBr∙CHCl_3_∙H_2_O (554.77): calculated, C 49.79, H 5.81, N 5.05; found, C 50.09, H 5.79, N 5.40.

1-Adamantyl-3-benzyloxy-4,5-diphenylimidazolium bromide (**4d**): 475 mg (88%). Colorless solid, m.p. 180–182 °C. ^1^H-NMR (CDCl_3_, 600 MHz): δ 1.54–1.62 (m, 6 H, Ad), 2.14 (m_c_, 3 H, Ad), 2.27 (m_c_, 6 H, Ad), 5.58 (s, 2 H, Bn), 7.10–7.12, 7.19–7.34, 7.37–7.39, 7.44–7.46 (4 m, 2 H, 10 H, 2 H, 1 H, 2 Ph, Bn), 10.79 (s, 1 H, C(2)H) ppm. ^13^C-NMR (CDCl_3_, 151 MHz): δ 29.7, 35.0, 42.5 (d, t, t, Ad), 66.5 (s, Ad), 84.0 (t, Bn), 123.1, 127.3 (2 s, Im), 128.4, 128.5, 128.6, 129.68, 129.71, 129.9, 130.6, 131.0, 132.4 (9 d, 2 Ph, Bn), 128.3, 130.4, 131.5 (3 s, 2 Ph, Bn), 132.9 (d_br_, C(2)) ppm. IR (neat): *ν* 2911, 1444, 1157, 911 cm^−1^. C_32_H_33_N_2_OBr·1.5 CHCl_3_ (720.58): calculated, C 55.84, H 4.83, N 3.89; found, C 55.70, H 5.24, N 4.45.

1,3-Dibenzyloxy-4,5-dimethylimidazolium bromide (**5a**): 384 mg (99%). Colorless solid, m.p. 110–111 °C. ^1^H-NMR (CDCl_3_, 600 MHz): δ 1.77 (s, 6 H, 2 Me), 5.77 (s, 4 H, 2 Bn), 7.35–7.43, 7.52–7.55 (2 m, 6 H, 4 H, 2 Bn), 11.80 (s, 1 H, C(2)H) ppm. ^13^C-NMR (CDCl_3_, 151 MHz): δ 7.0 (q, 2 Me), 84.1 (t, 2 Bn), 122.3 (s, C(4), C(5)), 128.9 (d, 4 CH, 2 Bn), 129.8 (d_br_, C(2)), 130.3, 130.8 (2 d, 6 CH, 2 Bn), 131.8 (s, 2 *i*-C, 2 Bn) ppm. IR (neat): *ν* 2816, 1623, 1455, 1388, 1215, 1075, 947, 904 cm^−1^. Crude sample of 5a was transformed into analytically pure imidazole-2-thione 7c (see below).

1,3-Di-(2-methylbenzyloxy)-4,5-dimethylimidazolium bromide (**5b**): 374 mg (90%). Colorless solid, m.p. 132–133 °C. ^1^H-NMR (CDCl_3_, 600 MHz): δ 1.74 (s, 6 H, 2 Me), 2.45 (s, 6 H, 2 Me), 5.75 (s, 4 H, 2 CH_2_), 7.09–7.11, 7.18–7.20, 7.25–7.28, 7.40–7.43 (4 m, 2 H each), 11.64 (s, 1 H, C(2)H) ppm. ^13^C-NMR (CDCl_3_, 151 MHz): δ 6.8 (q, 2 Me), 19.1 (q, 2 Me), 82.6 (t, 2 CH_2_), 122.3 (s, C(4), C(5)), 126.3 (d, 2 CH), 129.7 (d_br_, C(2)), 130.1 (s, 2 *i*-C), 130.6, 130.7, 132.0 (3 d, 6 CH), 138.7 (s, 2 *i*-C) ppm. IR (neat): *ν* 2825, 2691, 1629, 1461, 1440, 1392, 1215, 1081, 922, 871, 749 cm^−1^. C_21_H_25_N_2_O_2_Br (417.3): calculated, C 60.44, H 6.04, N 6.71; found, C 60.29, H 5.95, N 7.43.

1,3-Di-(4-methylbenzyloxy)-4,5-dimethylimidazolium bromide (**5c**): 404 mg (97%). Colorless solid, m.p. 111–113 °C. ^1^H-NMR (CDCl_3_, 600 MHz): δ 1.75 (s, 6 H, 2 Me), 2.31 (s, 6 H, 2 Me), 5.64 (s, 4 H, 2 CH_2_), 7.09–7.12, 7.34–7.36 (2 m, 4 H each), 11.73 (s, 1 H, C(2)H) ppm. ^13^C-NMR (CDCl_3_, 151 MHz): δ 7.1 (q, 2 Me), 21.3 (q, 2 Me), 83.9 (t, 2 CH_2_), 122.2 (s, C(4), C(5)), 128.7 (s, 2 *i*-C), 129.4 (d_br_, C(2)), 129.5, 130.7 (2 d, 8 CH), 140.4 (s, 2 *i*-C) ppm. IR (neat): *ν* 2924, 2900, 1625, 1527, 1440, 1381, 1279, 1208, 919, 870, 807 cm^−1^. C_21_H_25_N_2_O_2_Br·H_2_O (435.3): calculated, C 57.94, H 6.25, N 6.43; found, C 57.01, H 6.23, N 6.86.

1,3-Di-(3,5-dimethylbenzyloxy)-4,5-dimethylimidazolium bromide (**5d**): 391 mg (88%). Colorless solid, m.p. 141–143 °C. ^1^H-NMR (CDCl_3_, 600 MHz): δ 1.89 (s, 6 H, 2 Me), 2.24 (s, 12 H, 4 Me), 5.55 (s, 4 H, 2 CH_2_), 6.99 (s_br_, 2 H), 7.06 (s_br_, 4 H), 11.62 (s, 1 H, C(2)H) ppm. ^13^C-NMR (CDCl_3_, 151 MHz): δ 7.1 (q, 2 Me), 21.0 (q, 4 Me), 84.5 (t, 2 CH_2_), 122.2 (s, C(4), C(5)), 128.1 (d, 4 CH), 129.6 (d_br_, C(2)), 131.4 (s, 2 *i*-C), 131.8 (d, 2 CH), 138.5 (s, 4 *i*-C) ppm. IR (neat): *ν* 2917, 1610, 1459, 1384, 1079, 934, 896 cm^−1^. C_23_H_29_N_2_O_2_Br·H_2_O (463.4): calculated, C 59.61, H 6.74, N 6.05; found, C 59.54, H 7.00, N 6.42.

1-Benzyloxy-3-(2-methylbenzyloxy)-4,5-dimethylimidazolium bromide (**5e**) (in a ca. 20:1:1 mixture with 5a and 5b: yield 383 mg (95%)). Pale yellow oil. ^1^H-NMR (CDCl_3_, 600 MHz): δ 1.73, 1.80, 2.46 (3 s, 3 H each, 3 Me), 5.72, 5.73 (2 s, 2 H each, 2 CH_2_), 7.07–7.10, 7.18–7.21, 7.26–7.30, 7.33–7.40, 7.52–7.55 (5 m, 1 H, 1 H, 1 H, 4 H, 2 H), 11.59 (s, 1 H, C(2)H) ppm. ^13^C-NMR (CDCl_3_, 151 MHz): δ 6.8, 7.0, 19.0 (3 q, 3 Me), 82.6, 84.0 (2 t, 2 CH_2_), 122.3, 122.4 (2 s, Im), 126.2, 128.8 (2 d, 3 CH), 129.3 (d_br_, C(2)), 130. (s, *i*-C), 130.2, 130.57, 130.60, 130.63 (4 d, 5 CH), 131.6 (s, *i*-C), 131.9 (d, CH), 138.6 (s, *i*-C) ppm. IR (neat): *ν* 2924, 1456, 1387, 1215, 1079, 911, 870, 749 cm^−1^. 

1-Benzyloxy-3-(4-methylbenzyloxy)-4,5-dimethylimidazolium bromide (**5f**): 380 mg (98%). Colorless solid, m.p. 124–126 °C. ^1^H-NMR (CDCl_3_, 600 MHz): δ 1.73, 1.74 (2 s, 3 H each, 2 Me), 2.30 (s, 3 H, Me), 5.62, 5.68 (2 s, 2 H each, 2 CH_2_), 7.09–7.11, 7.25–7.37, 7.45–7.47 (3 m, 2 H, 5 H, 2 H), 11.68 (s, 1 H, C(2)H) ppm. ^13^C-NMR (CDCl_3_, 151 MHz): δ 7.04, 7.06 (2 q, 2 Me), 21.3 (q, Me), 83.92, 83.95 (2 t, 2 CH_2_), 122.2, 122.3 (2 s, Im), 128.6 (s), 128.8 (d, 2 CH), 129.9 (d_br_, C(2)), 129.5, 130.2, 130.66, 130.67 (4 d, 7 CH), 131.6, 140.4 (2 s) ppm. IR (neat): *ν* 2917, 1449, 1375, 1215, 1077, 876 cm^−1^. C_20_H_23_N_2_O_2_Br (403.3): calculated, C 59.56, H 5.75, N 6.95; found, C 59.63, H 5.76, N 7.15.

1-Benzyloxy-3-(3,5-dimethylbenzyloxy)-4,5-dimethylimidazolium bromide (**5g**) (in a 10:1 mixture with 5d: yield 404 mg (97%)). Colorless oil. ^1^H-NMR (CDCl_3_, 600 MHz): δ 1.79, 1.85 (2 s, 3 H each, 2 Me), 2.27 (s, 6 H, 2 Me), 5.59, 5.69 (2 s, 2 H each, 2 CH_2_), 7.01 (s_br_, 1 H), 7.09 (s_br_, 2 H), 7.30–7.39, 7.46–7.48 (2 m, 3 H, 2 H), 11.64 (s, 1 H, C(2)H) ppm. ^13^C-NMR (CDCl_3_, 151 MHz): δ 7.0, 7.1 (2 q, 2 Me), 21.0 (q, 2 Me), 84.2, 84.5 (2 t, 2 CH_2_), 122.3, 122.4 (2 s, Im), 128.2, 128.9 (2 d, 4 CH), 129.4 (d_br_, C(2)), 130.2, 130.6 (2 d, 3 CH), 131.5, 131.7 (2 s, 2 *i*-C), 131.8 (d, CH), 138.5 (s, 2 *i*-C) ppm. IR (neat): *ν* 2923, 1472, 1375, 911, 898 cm^−1^.

1-Adamantyloxy-3-benzyloxy-4,5-dimethylimidazolium bromide (**5h**): 346 mg (80%). Colorless solid, m.p. 131–132 °C. ^1^H-NMR (CDCl_3_, 600 MHz): δ 1.61-1.69 (m, 6 H, Ad), 1.93 (m_c_, 6 H, Ad), 2.02, 2.16 (2 s, 3 H each, 2 Me), 2.32 (m_c_, 3 H, Ad), 5.84 (s, 2 H, Bn), 7.36–7.40, 7.60–7.63 (2 m, 3 H, 2 H, Bn), 11.42 (s, 1 H, C(2)H) ppm. ^13^C-NMR (CDCl_3_, 151 MHz): δ 7.4, 8.2 (2 q, 2 Me), 31.2, 35.3, 40.6 (d, t, t, Ad), 84.1 (t, Bn), 91.4 (s, Ad), 122.7, 123.4 (2 s, Im), 128.9, 130.2, 131.0 (3 d, Bn), 131.3 (d_br_, C(2)), 131.9 (s, Bn) ppm. IR (neat): *ν* 2902, 2851, 1638, 1456, 1358, 1217, 1049, 889, cm^−1^. C_22_H_29_N_2_O_2_Br∙0.5 H_2_O (442.4): calculated, C 59.73, H 6.83, N 6.33; found, C 59.54, H 7.00, N 6.42.

1-Adamantyloxy-3-(2-methylbenzyloxy)-4,5-dimethylimidazolium bromide (**5i**): 374 mg (86%). Colorless solid, m.p. 152–154 °C. ^1^H-NMR (CDCl_3_, 600 MHz): δ 1.62–1.68 (m, 6 H, Ad), 1.93 (s_br_, 3 H, Me), 1.95–1.98 (m, 6 H, Ad), 2.17 (s_br_, 3 H, Me), 2.33 (m_c_, 3 H, Ad), 2.51 (s, 3 H, Me), 5.88 (s, 2 H, Bn), 7.13–7.15, 7.22–7.24, 7.29–7.33, 7.49–7.51 (4 m, 1 H each), 11.47 (s, 1 H, C(2)H) ppm. ^13^C-NMR (CDCl_3_, 151 MHz): δ 7.2, 8.2, 19.2 (3 q, 3 Me), 31.2, 35.3, 40.6 (d, t, t, Ad), 82.8 (t, CH_2_), 91.5 (s, Ad), 122.9, 123.4 (2 s, Im),126.3 (d, CH), 130.4 (s, *i*-C), 130.6, 130.7 (2 d, 2 CH), 131.3 (d_br_, C(2)), 132.5 (d, CH), 138.7 (s, *i*-C) ppm. IR (neat): *ν* 2906, 2849, 1738, 1358, 1216, 1048, 889, 743, cm^−1^. C_23_H_31_N_2_O_2_Br·CHCl_3_ (566.8): calculated, C 50.86, H 5.69, N 4.94; found, C 50.16, H 5.82, N 5.26.

1-Adamantyloxy-3-(4-methylbenzyloxy)-4,5-dimethylimidazolium bromide (**5j**): 342 mg (78%). Colorless solid, m.p. 130–132 °C. ^1^H-NMR (CDCl_3_, 600 MHz): δ 1.59–1.67 (m, 6 H, Ad), 1.92 (m_c_, 6 H, Ad), 2.01, 2.16 (2 s, 3 H each, 2 Me), 2.33 (m_c_, 3 H, Ad), 2.33 (s, 3 H, Me), 5.77 (s, 2 H, CH_2_), 7.15–7.17, 7.46–7.48 (2 m, 2 H each), 11.38 (s, 1 H, C(2)H) ppm. ^13^C-NMR (CDCl_3_, 151 MHz): δ 7.4, 8.2, 21.4 (3 q, 3 Me), 31.2, 35.3, 40.6 (d, t, t, Ad), 84.1 (t, CH_2_), 91.4 (s, Ad), 122.8, 123.3 (2 s, Im), 128.9 (s, *i*-C), 129.6, 131.0 (2 d, 4 CH), 131.3 (d_br_, C(2)), 140.4 (s, *i*-C) ppm. IR (neat): *ν* 2911, 2853, 1738, 1378, 1354, 1216, 1043, 879, 813, cm^−1^. C_23_H_31_N_2_O_2_Br (447.4): calcd. C 61.74, H 6.98, N 6.26; found: C 61.54, H 7.26, N 6.21.

1-Adamantyloxy-3-(3,5-dimethylbenzyloxy)-4,5-dimethylimidazolium bromide (**5k**): 306 mg (66%). Pale yellow solid, m.p. 150–152 °C. ^1^H-NMR (CDCl_3_, 600 MHz): δ 1.60–1.66 (m, 6 H, Ad), 1.92–1.94 (m, 6 H, Ad), 2.01, 2.16 (2 s, 3 H each, 2 Me), 2.28 (s, 6 H, 2 Me), 2.31 (m_c_, 3 H, Ad), 5.71 (s, 2 H, CH_2_), 7.01 (s_br_, 1 H), 7.15 (s_br_, 2 H), 11.43 (s, 1 H, C(2)H) ppm. ^13^C-NMR (CDCl_3_, 151 MHz): δ 7.4, 8.2 (2 q, 2 Me), 21.1 (q, 2 Me), 31.2, 35.3, 40.7 (d, t, t, Ad), 84.6 (t, CH_2_), 91.4 (s, Ad), 122.8, 123.3 (2 s, Im), 128.5 (d, 2 CH), 131.4 (d_br_, C(2)), 131.76 (s, *i*-C), 131.84 (d, CH), 138.5 (s, 2 *i*-C) ppm. IR (neat): *ν* 2910, 2848, 1738, 1359, 1209, 1050, 890, 844 cm^−1^. C_24_H_33_N_2_O_2_Br (461.43): calculated, C 62.47, H 7.21, N 6.07; found, C 62.10, H 7.50, N 5.80.

#### 2.1.3. General Procedure for the Synthesis of Hexafluorophosphates 6 

To a solution of the corresponding imidazolium bromide (0.5 mmol) in H_2_O (6 mL, in the case of bromide **4b**) or EtOH (5 mL, in the case of bromide **5k**) was added dropwise an excess of NH_4_PF_6_ (87 mg, 0.54 mmol) in H_2_O (2 mL) under vigorous stirring, at room temperature. After ca. 30 min, the precipitated crude hexafluorophosphate **6** was filtered and dried under vacuum.

1-Benzyl-3-benzyloxy-4,5-diphenylimidazolium hexafluorophosphate (**6a**): 222 mg (79%). Colorless solid, m.p. 163–166 °C (CH_2_Cl_2_/diisopropyl ether). ^1^H-NMR (CDCl_3_, 600 MHz): δ 5.35, 5.50 (2 s, 2 H each, 2 CH_2_), 7.04–7.08, 7.14–7.27, 7.36–7.40, 7.46–7.49 (4 m, 2 H, 14 H, 3 H, 1 H, 2 Ph, 2 Bn), 10.96 (s, 1 H, C(2)H) ppm. ^13^C-NMR (CDCl_3_, 151 MHz): δ 51.4 (t, NBn), 84.4 (t, OBn), 122.9, 124.3 (2 s, Im), 128.7*, 128.8, 129.01, 129.3, 129.5, 130.0, 130.6, 130.8, 131.0 (9 d, 20 CH, 2 Ph, 2 Bn), 128.99, 130.2, 130.9, 133.1 (4 s, 2 Ph, 2 Bn), 133.6 (d_br_, C(2)) ppm; *higher intensity. IR (neat): *ν* 2861, 1456, 1338, 1163, 833, 754, 690 cm^−1^. C_29_H_25_N_2_OPF_6_ (562.5): calculated, C 61.92, H 4.48, N 4.98; found, C 61.53, H 4.35 N 5.17.

1-Adamantyloxy-3-(3,5-dimethylbenzyloxy)-4,5-dimethylimidazolium hexafluorophosphate (**6b**): 157 mg (63%). Colorless solid, m.p. 150–152 °C (EtOH/H_2_O). ^1^H-NMR (CDCl_3_, 600 MHz): δ 1.59–1.66 (m, 6 H, Ad), 1.75–1.78 (m, 6 H, Ad), 2.23, 2.23 (2 s, 3 H each, 2 Me), 2.28 (m_c_, 3 H, Ad), 2.32 (s, 6 H, 2 Me), 5.32 (s, 2 H, CH_2_), 7.06 (s_br_, 1 H), 7.10 (s_br_, 2 H), 8.30 (s, 1 H, C(2)H) ppm. ^13^C-NMR (CDCl_3_, 151 MHz): δ 7.3, 8.3 (2 q, 2 Me), 21.1 (q, 2 Me), 31.3, 35.2, 40.3 (d, t, t, Ad), 84.0 (t, CH_2_), 91.8 (s, Ad), 124.1, 125.1 (2 s, Im), 127.5 (d, C(2)), 128.3 (d, 2 CH), 131.4 (s, *i*-C), 132.0 (d, CH), 139.0 (s, 2 *i*-C) ppm. IR (neat): *ν* 3144, 2920, 1449, 1294, 1039, 826 cm^−1^. C_24_H_33_N_2_O_2_PF_6_ (526.5): calculated, C 54.75, H 6.32, N 5.32; found, C 54.71, H 6.36, N 5.54.

#### 2.1.4. General Procedure for the Synthesis of Imidazole-2-Thiones 7

To a solution of 4,5-dimethylimidazolium bromide of type **4** or **5** (0.50 mmol) in dry pyridine (2.0 mL) was added Et_3_N (100 μL, 0.75 mmol), followed by a slight excess of elemental sulfur (19.2 mg, 0.60 mmol) at room temperature, and the resulting homogeneous solution was stirred magnetically for 24 h. After removal of solvents in vacuo, the resulting crude products were purified by recrystallization from MeOH to give *N*-benzyloxy-imidazole-2-thione **7**.

1-Benzyl-3-benzyloxy-4,5-dimethylimidazole-2-thione (**7a**): 122 mg (75%). Colorless crystals, m.p. 116–117 °C (MeOH). ^1^H-NMR (600 MHz, CDCl_3_): δ 1.79, 1.87 (2 s, 3 H each, 2 Me), 5.35 (s, 2 H, NCH_2_), 5.47 (s, 2 H, OCH_2_), 7.24–7.28, 7.31–7.33, 7.36–7.40, 7.50–7.52 (4 m, 3 H, 2 H, 3 H, 2 H, 2 Bn) ppm. ^13^C--NMR (CDCl_3_, 151 MHz): δ 7.4, 9.2 (2 q, 2 Me), 47.8 (t, NCH_2_), 78.0 (t, OCH_2_), 117.7, 120.0 (2 s, Im), 127.0, 127.6, 128.6, 128.7, 129.3, 130.4 (6 d, 2 Bn), 133.9, 136.3 (2 s, 2 Bn), 157.4 (s, C=S) ppm. IR (neat): *ν* 2924, 1403, 1340, 997 cm^−1^. ESI-MS (*m***/***z*): 347.3 (33, [*M* + Na]^+^), 325.4 (100, [*M* + H]^+^), 293.4 (31). C_19_H_20_N_2_OS (324.1): calculated, C 70.34, H 6.21, N 8.63, S 9.88; found, C 70.24, H 6.28, N 8.77, S 9.79.

1-Adamantyl-3-benzyloxy-4,5-dimethylimidazole-2-thione (**7b**): 95 mg (52%). Colorless crystals, m.p. 99–101 °C (MeOH). ^1^H-NMR (CDCl_3_, 600 MHz): δ 1.67-1.69 (m, 3 H, Ad), 1.82 (s, 3 H, Me), 1.83 (m_c_, 3 H, Ad), 2.19 (s, 3 H, Me), 2.21 (m_c_, 3 H, Ad), 2.83–2.85 (m, 6 H, Ad), 5.40 (s, 2 H, Bn), 7.33–7.37, 7.46–7.50 (2 m, 3 H, 2 H, Bn) ppm. ^13^C-NMR (CDCl_3_, 151 MHz): δ 7.8, 15.2 (2 q, 2 Me), 30.4, 36.0, 40.6 (d, t, t, Ad), 65.7 (s, Ad), 77.4 (t, Bn), 118.0, 121.0 (2 s, Im), 128.4, 129.1, 130.2 (3 d, Bn), 134.2 (s, Bn), 156.3 (s, C=S) ppm. IR (neat): *ν* 2913, 2851, 1454, 1360, 1273, 1250, 956 cm^−1^. ESI-MS (*m***/***z*): 369.3 (100, [*M* + H]^+^). C_22_H_28_N_2_OS (368.2): calculated, C 71.70, H 7.66, N 7.60, S 8.70; found, C 71.68, H 7.84, N 7.76, S 8.54.

1,3-Dibenzyloxy-4,5-dimethylimidazole-2-thione (**7c**): 124 mg (73%). Colorless crystals, m.p. 87–88 °C (MeOH). ^1^H-NMR (600 MHz, CDCl_3_): δ 1.68 (s, 6 H, 2 Me), 5.45 (s, 4 H, 2 CH_2_), 7.36–7.40, 7.47–7.59 (2 m, 6 H, 4 H, 2 Bn) ppm. ^13^C-NMR (CDCl_3_, 151 MHz): δ 7.3 (q, 2 Me), 78.3 (t, 2 Bn), 116.8 (s, C(4), C(5)), 128.6, 129.4, 130.5 (3 d, 2 Bn), 133.8 (s, 2 Bn), 152.8 (s, C=S) ppm. IR (neat): *ν* 2917, 1456, 1403, 1384, 1081, 956, 917 cm^−1^. ESI-MS (*m***/***z*): 363.3 (100, [*M* + Na]^+^), 341.3 (46, [*M* + H]^+^). C_19_H_20_N_2_O_2_S (340.1): calculated, C 67.03, H 5.92, N 8.23, S 9.42; found, C 67.16, H 5.99, N 8.35, S 9.52.

1-Adamantyloxy-3-benzyloxy-4,5-dimethylimidazole-2-thione (**7d**): 91 mg (48%). Colorless crystals, m.p. 108–109 °C (MeOH). ^1^H-NMR (600 MHz, CDCl_3_): δ 1.65 (m_c_, 6 H, Ad), 1.76, 2.05 (2 s, 3 H each, 2 Me), 2.15, 2.25 (2 m_c_, 6 H, 3 H Ad), 5.42 (s_br_, 2 H, Bn), 7.35–7.39, 7.47–7.49 (2 m, 3 H, 2 H, Bn) ppm. ^13^C-NMR (CDCl_3_, 151 MHz): δ 7.6, 9.4 (2 q, 2 Me), 31.5, 35.9, 42.0 (d, t, t, Ad), 77.9 (t, Bn), 89.0 (s, Ad), 117.2, 118.4 (2 s, Im), 128.5, 129.3, 130.5 (3 d, Bn), 134.0 (s, Bn), 157.0 (s, C=S) ppm. IR (neat): *ν* 2909, 2850, 1385, 1353, 1045, 945, 906 cm^−1^. ESI-MS (*m***/***z*): 385.2 (100, [*M* + H]^+^), 353.2 (93). C_22_H_28_N_2_O_2_S (384.2): calculated, C 68.72, H 7.34, N 7.29, S 8.34; found, C 68.65, H 7.39, N 7.23, S 8.20.

### 2.2. Cell Lines and Cell Culture

The promyelocytic leukemia HL-60 and breast cancer adenocarcinoma MCF-7 cell lines were purchased from the European Collection of Cell Cultures (ECACC). Leukemia cells were cultured in Roswell Park Memorial Institute (RPMI) 1640 plus GlutaMax I medium (Gibco/Life Technologies, Carlsbad, CA, USA). MCF-7 cells were maintained in Minimum Essential Medium Eagle (Sigma Aldrich, St. Louis, MO, USA) supplemented with 2 mM glutamine and MEM nonessential amino-acid solution (Sigma Aldrich, St. Louis, MO, USA). Both media were supplemented with 10% heat-inactivated fetal bovine serum (Biological Industries, Beit-Haemek, Israel) and antibiotics (100 U/mL penicillin and 100 μg/mL streptomycin) (Sigma-Aldrich, St. Louis, MO, USA). Human umbilical vein endothelial cells (HUVECs) and human mammary gland/breast cell line MCF-10A were purchased from the American Type Culture Collection (ATCC). HUVECs were cultured using the EGM-2 Endothelial Medium BulletKit, whereas MCF-10A was cultured using the MEGM Mammary Epithelial BulletKit, both purchased from Lonza (Lonza, Walkersville, MD, USA). Cells were maintained at 37 °C in 5% CO_2_ atmosphere and grown until 80% confluent.

### 2.3. In Vitro Cytotoxicity Assay

The MTT (3-(4, 5-dimethylthiazol-2-yl)-2, 5-diphenyltetrazolium bromide) assay was performed according to the known procedure [21]. Cells were seeded into 24-well plates at a density of 8 × 10^4^/mL and left to grow for 24 h. After being cultured for 48 h with various concentrations of the tested compounds, cells were incubated with MTT solution (5 mg/mL in phosphate-buffered saline) for 2 h. Then, the plates were centrifuged and the supernatant was discarded. Dimethyl sulfoxide (DMSO; 1 mL) was added to each well to dissolve the blue formazan product, whose absorbance was measured at 560 nm using a FlexStation 3 Multi-Mode Microplate Reader (Molecular Devices, LLC, CA, USA). The untreated cells were used as control. The data were expressed as mean ± SEM of three independent experiments.

## 3. Results and Discussion

**Scheme 1:**
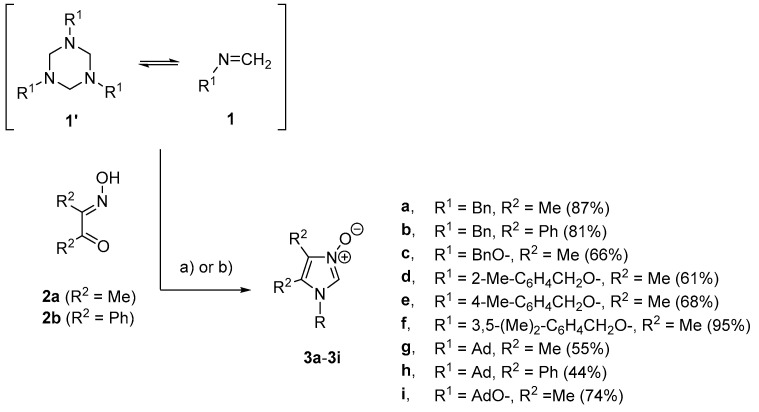
Applications of *N*-alkyl and *N*-alkoxy substituted Schiff bases (as monomeric formaldimines **1** and/or trimeric hexahydro-[1,3,5]triazines **1′** (see the main text for details) in the synthesis of imidazole *N*-oxides **3** via cyclocondensation with diacetyl monooxime (**2a**) or benzil monooxime (**2b**). *Reagents and conditions*: (a) EtOH, reflux, 3 h or (b) AcOH, room temperature (rt), overnight, then solid Na_2_CO_3_.

According to the general protocol, condensation of *N*-alkyl formaldimines **1** with α-hydroxyiminoketones of type **2** in boiling ethanol leads to 2-unsubstituted imidazole *N*-oxides **3** (Scheme 1) [22].

Thus, starting with model *N*-benzylformaldimine (**1a**) available as the respective trimer **1′a** (namely, 1,3,5-tribenzylhexahydro-[1,3,5]triazine) and **2a** or **2b**, the expected products **3a** and **3b** were isolated in high yields of 87% and 81%, respectively. In contrast to **1a**, the *N*-benzyloxyformaldimine (**1b**) appeared exclusively in monomeric form [23], and the attempted synthesis of the corresponding imidazole *N*-oxide **3c** via condensation with diacetyl monoxime (**2a**) in boiling EtOH was unsuccessful. However, when the reaction of **1b** with **2a** was repeated in glacial AcOH at room temperature in an overnight experiment, the target 1-benzyloxy-4,5-dimethylimidazole *N*-oxide (**3c**) was obtained in satisfactory yield (66%). Apparently, the application of acetic acid acting as a catalyst is necessary to initiate the cyclization reaction of less electrophilic *N*-alkoxy-formaldimines such as **1b**. On the basis of this observation, analogous imidazole *N*-oxides **3d**–**3f** bearing Me groups attached to the aromatic ring were successfully prepared and isolated as colorless solids in 61–95% yield. In extension of the series, 1-adamantyl- and 1-adamantyloxy-formaldimines **1f** and **1g**, respectively, were also involved in the study to provide imidazole *N*-oxides **3g**–**3i** with bulky Ad (1-adamantyl) moiety attached to N(1) atom of imidazole ring. The introduction of this group was aimed at tuning the biological activity by increasing the lipophilic character of the target products, as often observed for various organic compounds [24].

**Scheme 2:**
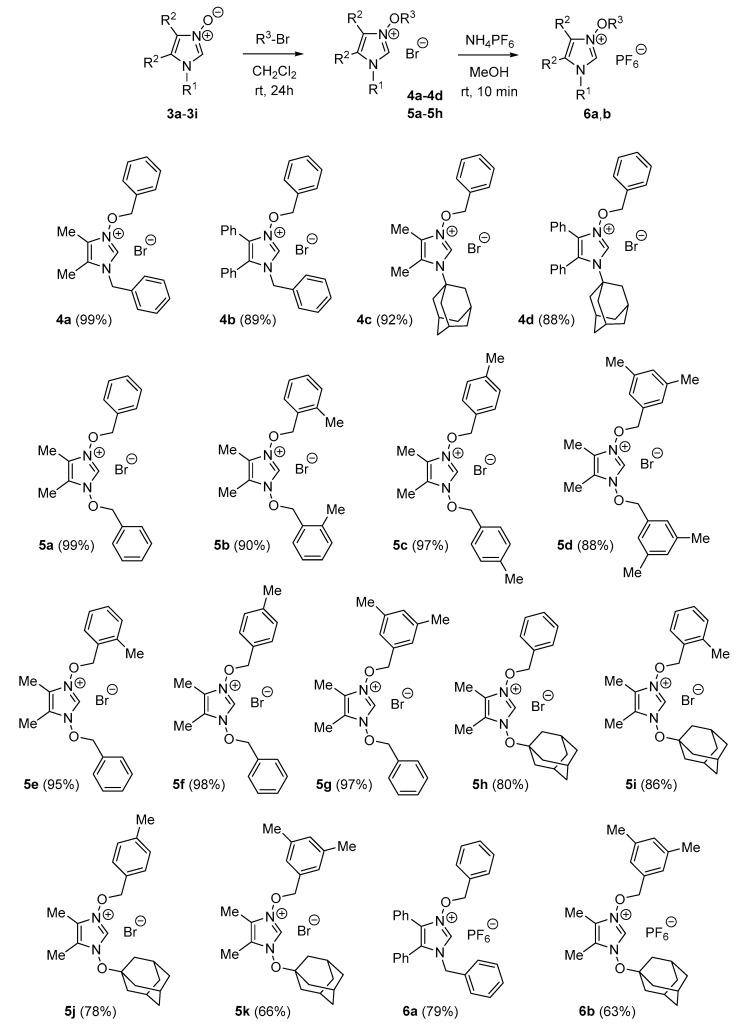
Synthesis of 4,5-dimethylimidazolium bromides **4** and **5** via *O*-alkylation of imidazole *N*-oxides **3**, and counterion exchange in selected bromides **4** and **5** leading to hexafluorophosphates **6a**–**6b**.

The first *O*-benzylations were performed starting with *N*-benzyl- and *N*-benzyloxy-imidazole *N*-oxides **3a** and **3c**, and were typically carried out in CH_2_Cl_2_ solutions, at room temperature, using a slight excess of benzyl bromide as an alkylating agent [25]. In both cases, the anticipated *O*-benzylation provided exclusively the respective nonsymmetric and symmetric imidazolium salts **4a** and **5a** as model compounds of type **II** and **III**, respectively (Figure 1 and Scheme 2).

Simple work-up by triturating of the crude reaction mixtures with several portions of dry Et_2_O allowed nearly quantitative isolation of spectroscopically pure products.

The structure of the obtained imidazolium bromides **4a** and **5a** was confirmed by spectroscopic methods. For example, in the ^1^H-NMR spectrum of nonsymmetric salt **4a**, the absorptions attributed to two Me groups were found at 1.92 and 2.07 ppm in the ^1^H-NMR. Although the signals of two nonequivalent –CH_2_– and –OCH_2_– groups in **4a** showed only little difference of the chemical shifts in the ^1^H-NMR spectrum (5.52 and 5.57 ppm), their absorptions in ^13^C-NMR found at 51.3 and 84.0 ppm clearly matched the proposed structure. On the other hand, the absorption in ^1^H-NMR of two spectroscopically equal Me groups in bis-alkoxyimidazolium salt **5a** was found at 1.77 ppm, along with the single signal at 5.77 ppm attributed to both –OCH_2_– units. Moreover, the absorptions of C(2)-*H* atom for both salts **4a** and **5a** were found at 11.00 and 11.80 ppm, respectively. As expected, these highly diagnostic signals were significantly low-field-shifted in comparison to their *N*-oxide precursors (7.88 ppm for **3a**, and 7.73 ppm for **3c**). The ^13^C-NMR spectra of both model salts clearly confirmed the postulated structures. Particularly, the diagnostic broadened signals of the C(2) atoms in **4a** and **5a** were found at 132.5 and 129.8 ppm, respectively. Similar results were obtained in the case of the synthesis of further C_2_-symmetric imidazolium salts **5b**–**5d**; in all these cases, only one set of signals attributed to the benzyloxy groups in both ^1^H- and ^13^C-NMR was found.

**Scheme 3:**
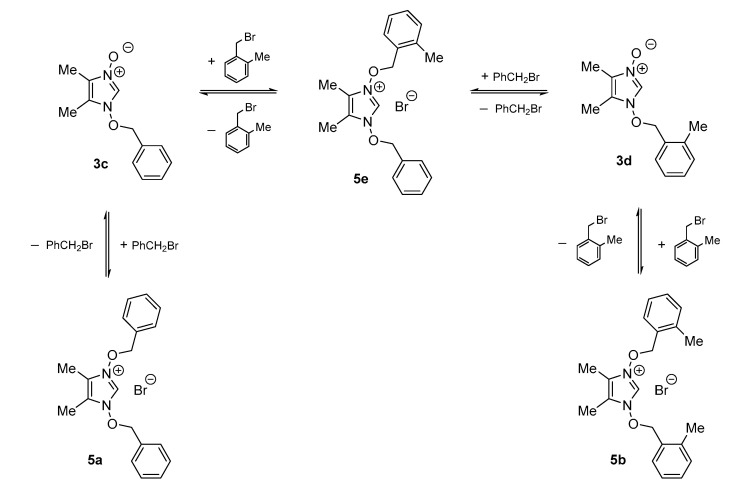
The “benzyl dance” in the attempted synthesis of nonsymmetric imidazolium bromide **5e** via *O*-alkylations of imidazole *N*-oxides **3c** and **3d**.

Preparation of nonsymmetric imidazolium bromides **5e**–**5g** with benzyl bromide also occurred smoothly at room temperature, starting with imidazole *N*-oxides **3d**–**3f** bearing at the N(1) atom 2-methylbenzyloxy, 4-methylbenzyloxy, and 3,5-dimethylbenzyloxy groups, respectively. However, whereas, in the case of 4-methyl-substituted derivative **3e,** the imidazolium salt **5f** was a sole product, in the two other cases, unexpectedly, mixtures of three different salts were detected on the basis of the ^1^H-NMR spectra of crude products. For example, careful analysis of the crude products obtained from *N*-oxide **3d** and benzyl bromide revealed the formation of two symmetric salts **5a** and **5b**, along with desired **5e** in a ratio of 3:10:4 (Scheme 3).

Attempted separation of this mixture via either chromatography or fractional crystallization was unsuccessful. For that reason, the alternative synthesis of **5e** via treatment of imidazole *N*-oxide **3c** with 2-methylbenzyl bromide as alkylating agent was performed under the same conditions (CH_2_Cl_2_, room temperature, overnight). In that case, the reaction resulted in the formation of desired imidazolium salt **5e** contaminated with small amounts of **5a** and **5b** (in ca. 20:1:1 ratio). A similar result was observed in benzylation of **3c** with 3,5-dimethylbenzyl bromide, which afforded **5g** as the major component accompanied by ca. 10% symmetric salt **5d**. All these results can be explained by the assumption that, in the studied system, the “benzyl dance” takes place. The observed phenomenon results, very likely, from the fact that *O*-benzylations of imidazole *N*-oxides such as **3d**–**3f** functionalized with another type of benzyloxy group at N(1) occur as a reversible process. Therefore, in the case of nonsymmetric salts such as **5e**–**5g**, two different alkylating agents operate in the system, and a mixture of three salts can be formed. This interesting observation deserves a separate study.

In order to supplement the 4,5-dimethyl-substituted imidazolium series, a representative 4,5-diphenyl-functionalized analogue **4b** was obtained by *O*-benzylation of imidazole *N*-oxide **3b**. In that case, smooth counterion exchange (Br^−^ → PF_6_^−^) was carried out by the treatment of starting bromide **4b** with NH_4_PF_6_ in methanolic solution at room temperature. The expected hexafluorophosphate **6a** precipitated from the solution and was isolated in fair yield (79%) as colorless crystals. The characteristic singlet of the C(2)-*H* of bromide **4b** appeared at 11.17 ppm, whereas, in the corresponding hexafluorophosphate **6a,** slight high-field shift of this diagnostic signal was observed (10.96 ppm).

Finally, the imidazole *N*-oxides **3g**, **3h**, and **3i** functionalized either with 1-adamantyl or with (adamant-1-yl)oxy group were also treated with benzyl bromides to afford the expected salts **4c**,**d** and **5h**–**5k**. Notably, in contrast to mentioned above problems in the synthesis of benzyloxyimidazolium salts **5e**–**5g**, the preparation of unsymmetrically substituted derivatives bearing adamantyloxy groups at the N atom occurred with excellent selectivity, and a single product was isolated in each case.

**Scheme 4:**
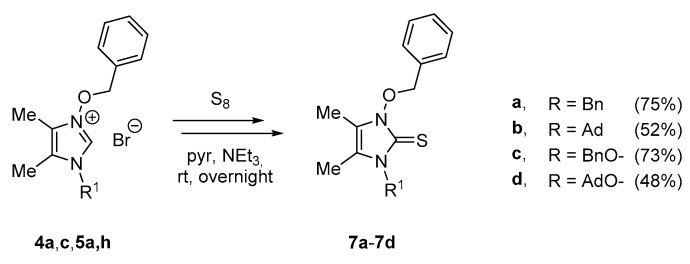
Sulfur-transfer reactions of imidazolium bromides **4** and **5** leading to non-enolisable *N*-alkoxy-functionalized imidazole-2-thiones **7** via in situ generated intermediate imidazol-2-ylidenes.

In situ generated *N*-heterocyclic carbenes (NHCs) are known to react with chalcogens such as O_2_, S_8_, and Se_8_, yielding imidazole-2-ones, imidazole-2-thiones, and imidazole-2-selones, respectively [26]. Treatment of imidazolium bromides **4a** and **5a** with Et_3_N in pyridine solutions, in the presence of elemental sulfur, led to the expected *N*-alkoxyimidazole-2-thiones **7a** and **7c**, respectively, which can formally be considered as new types of lepidiline derivatives. As depicted in Scheme 4, further *N*-alkoxyimidazole-2-thiones bearing either Ad and AdO groups were smoothly prepared in an analogous manner.

The structures of the isolated products **7a**–**d** were confirmed, e.g., by the presence of characteristic absorptions in ^13^C-NMR spectra attributed to the C=S group, which were found in the 152–158 ppm region.

It is well documented that alkoxyamines (oxime ethers) and their derivatives show diverse biological activities, and the hemolytic cleavage of the C–O bond is of great significance to generate active radical species (i.e., nitroxyls) [27]. For that reason, modification of the lepidiline structure by the replacement of *N*-benzyl with an *N*-benzyloxy group could be beneficial for the enhancement of their cytotoxicity. In the pioneering work on the isolation of lepidilines A and B from a root extract of *Lepidium meyenii*, their metabolic activity was tested against several human cancer cell lines, and lepidiline B was found to be highly cytotoxic for some of them (bladder carcinoma UMUC3, pancreatic adenocarcinoma PACA2, breast carcinoma MDA231, and ovarian carcinoma FDIGROV) [12]. Later on, cytotoxicity of some lepidiline analogues [28], as well as metal complexes of nucleophilic carbenes (NHCs), derived from lepidilines or related imidazole-based structures, was also reported [29,30,31]. 

In the present study, the cytotoxic activity of a series of 4,5-dimethylimidazolium salts **5** bearing either two benzyloxy (**5b**–**5f**) or benzyloxy and 1-adamantyloxy groups (**5h**–**5k**) located at the *N*-atoms of the core heterocycle, supplemented by structurally similar 4,5-diphenyl analogue **4b,** was evaluated against HL-60 and MCF-7 cell lines using the MTT assay. Generally, the analogues were more cytotoxic for HL-60 than for MCF-7 cells (Table 1).

In the course of the presented study, natural lepidilines were not available and, therefore, comparison of their activity with novel analogues was not possible.

On the HL-60 cell line, exceptional activity, below 1 µM, was observed for all compounds functionalized with the 1-adamantyloxy group (compounds **5h**–**5k**). Interestingly, introduction of methyl groups into the second benzyloxy substituent (compounds **51**–**5k**) seemed to slightly enhance activity in the series. Similarly, 4,5-diphenyl analogue **4b** exhibited a relatively low half maximal inhibitory concentration (IC_50_) value, comparable to that of the adamantyloxy derivatives. Compounds bearing benzyloxy substituents (compounds **5b**–**5f**) were slightly less, but still very cytotoxic, with IC_50_ values between 1.46 and 4.88 µM.

On MCF-7 cells, the highest cytotoxicity was observed for the unsymmetrically substituted **4b**. Again, molecules of adamantyloxy series, particularly those bearing *p*-tolyloxy (compound **5j**) and 3,5-dimethylbenzyloxy (compound **5k**) moieties as the second *N*-substituent, exhibited lower IC_50_ values in comparison to bis-benzyloxy imidazolium salts (compounds **5b**–**5f**). The activity of the latter only slightly varied depending on the position of methyl groups attached to the aromatic rings.

It is well established that biological activity of imidazolium salts varies with the type of the counterion present in the molecule [9]. For that reason, two selected hexafluorophosphates **6a** and **6b** derived from bromides **4b** and **5k**, respectively, were also examined. However, IC_50_ values of the bromides and corresponding hexafluorophosphates were almost identical on both cancer cell lines, indicating that, in the case of the tested imidazolium salts, counterions did not influence cytotoxicity. 

Selected compounds were also tested against HUVECs and MCF-10A cells, in order to evaluate their influence on normal, noncancerous cells. The most cytotoxic against leukemia cells compounds **5h**–**5k** were 4–6-fold less toxic for HUVECs. Such selectivity was not observed for MCF-7 versus MCF-10A cells, where similar or even lower IC_50_ values were observed for normal cells.

## 4. Conclusions

The present study showed that 2-unsubstituted imidazole *N*-oxides can be explored for a smooth and efficient preparation of alkoxy-analogues of naturally occurring imidazolium alkaloids known as lepidilines A and C. On the basis of the elaborated protocol, symmetric and nonsymmetric alkoxy-imidazolium bromides can be efficiently prepared and used for further transformations. For example, treatment with Et_3_N in pyridine solution leads to in situ generation of the corresponding imidazol-2-ylidenes (NHCs), which can be trapped by elemental sulfur to afford *N*-alkoxyimidazole-2-thiones. An interesting phenomenon was observed in the course of *O*-benzylations of *N*(1)-(methyl)benzyloxy-substituted imidazole *N*-oxides comprising the “benzyl dance”, leading to the formation of a mixture of two symmetric and one nonsymmetric bis-benzyloxyimidazolium bromides. Selected alkoxyimidazolium bromides were tested against tumor cell lines, HL-60 and MCF-7. Replacement of bromides into hexafluorophosphates did not influence cytotoxicity, pointing to the minimal role of the counterion in the biological activity of these salts. Taking into account the availability of starting materials and straightforward procedure, the presented method can be recommended for the preparation of alkoxy-analogues of lepidiline A and C alkaloids, even in a multigram scale.

## Figures and Tables

**Table 1:** In vitro cytotoxic activity of selected alkoxyimidazolium bromides **4** and **5** and hexafluorophosphates **6** tested on two cancer cell lines and two normal cell lines.

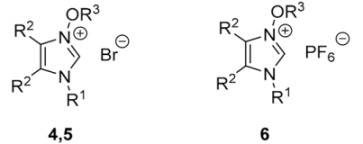							
**Compd.**	**R^1^**	**R^2^**	**R^3^**	**IC_50_ (μM) ^1^**			
				**HL-60**	**MCF-7**	**HUVEC**	**MCF-10A**
**4b**	Bn	Ph	Bn	0.70 ± 0.03	1.53 ± 0.09	0.97 ± 0.03	0.57 ± 0.01
**5b**	2-Me–C_6_H_4_CH_2_O–	Me	2-Me–C_6_H_4_CH_2_–	1.54 ± 0.01	8.30 ± 0.01	-	-
**5c**	4-Me–C_6_H_4_CH_2_O–	Me	4-Me-C_6_H_4_CH_2_–	4.88 ± 0.18	24.15 ± 0.46	-	-
**5d**	3,5-(Me)_2_–C_6_H_4_CH_2_O–	Me	3,5-(Me)_2_–C_6_H_4_CH_2_-	1.46 ± 0.02	8.57 ± 0.37	-	-
**5e ^2^**	BnO–	Me	2-Me–C_6_H_4_CH_2_–	3.16 ± 0.18	11.55 ± 0.25	-	-
**5f**	BnO–	Me	4-Me–C_6_H_4_CH_2_–	2.91 ± 0.08	16.60 ± 1.06	-	-
**5h**	AdO– ^3^	Me	Bn	0.88 ± 0.08	6.76 ± 0.08	3.70 ± 0.23	6.40 ± 0.03
**5i**	AdO–	Me	2-Me–C_6_H_4_CH_2_–	0.67 ± 0.01	5.95 ± 0.35	3.96 ± 0.08	-
**5j**	AdO–	Me	4-Me–C_6_H_4_CH_2_–	0.39 ± 0.01	2.63 ± 0.03	1.94 ± 0.01	1.69 ± 0.04
**5k**	AdO–	Me	3,5-(Me)_2_–C_6_H_4_CH_2_–	0.36 ± 0.01	2.40 ± 0.06	1.82 ± 0.08	1.36 ± 0.01
**6a**	Bn	Ph	Bn	0.64 ± 0.02	1.29 ± 0.01	-	-
**6b**	AdO–	Me	3,5-(Me)_2_–C_6_H_4_CH_2_–	0.37 ± 0.01	2.52 ± 0.03	-	-

^1^ Compound concentration required to inhibit metabolic activity by 50%. Values are expressed as mean ± SEM from concentration-response curves of at least three experiments. ^2^ Contaminated with **5a** and **5b** (*ca*. 5% each). ^3^ Ad = 1-adamantyl.

## Supplementary Materials

The following are available online at https://www.mdpi.com/1996-1944/13/18/4190/s1, Figures S1–S59: Collected copies of the ^1^H and ^13^C NMR spectra for all new compounds are available online as a separate file.
