# Supplementary material for: Synthesis, Selected Transformations, and Biological Activity of Alkoxy Analogues of Lepidilines A and C
Source: Materials (Basel). 2020 Sep 21;13(18):4190. doi: 10.3390/ma13184190 (PMC7560456; doi:10.3390/ma13184190)
Supplement: Supplementary file 1 [file materials-13-04190-s001.pdf]

materials

MDPI

Supporting Information

Synthesis, Selected Transformations, and Biological Activity of Alkoxy Analogues of Lepidilines A and C

Content:

- Copies of ^{1}H and ^{13}C NMR spectra of synthetized compounds S2–S27
- Copies of DEPT and HMQC spectra of selected compounds S28–S31

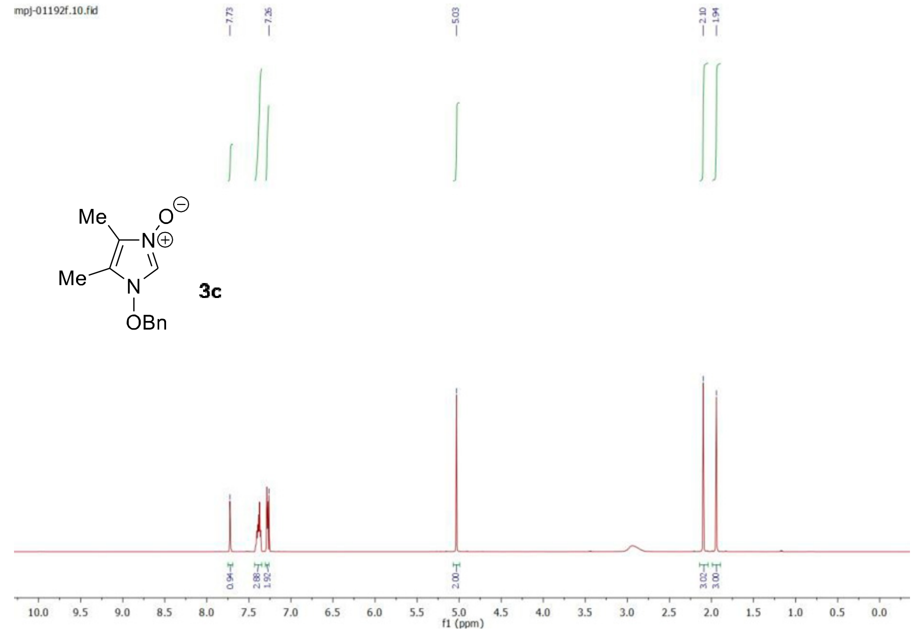

Figure S1. ^{1}H NMR of **3c** ($CDCl_3$, 600 MHz).

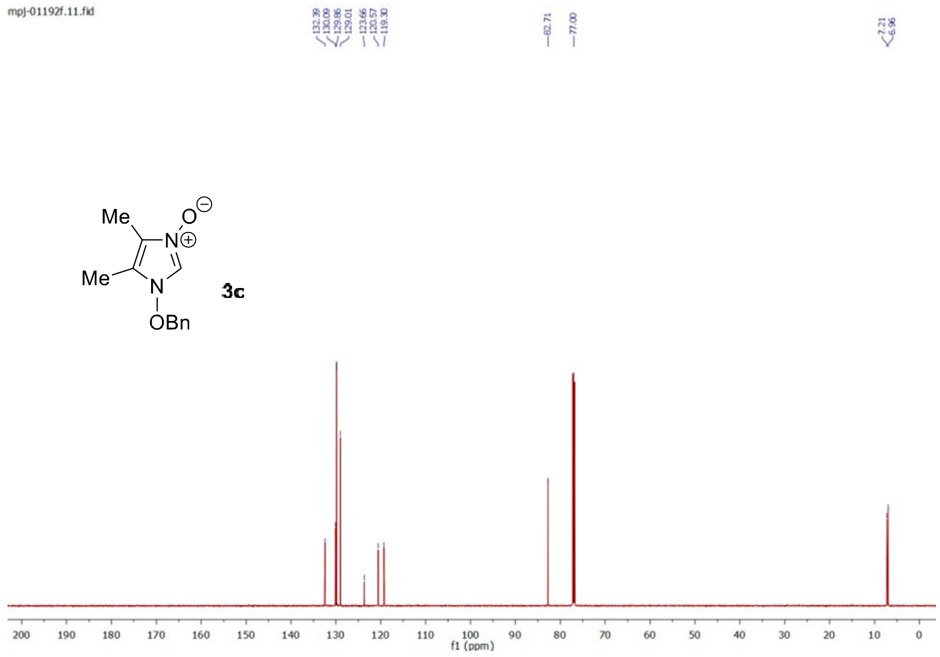

Figure S2. ^{13}C NMR of **3c** ($CDCl_3$, 151 MHz).

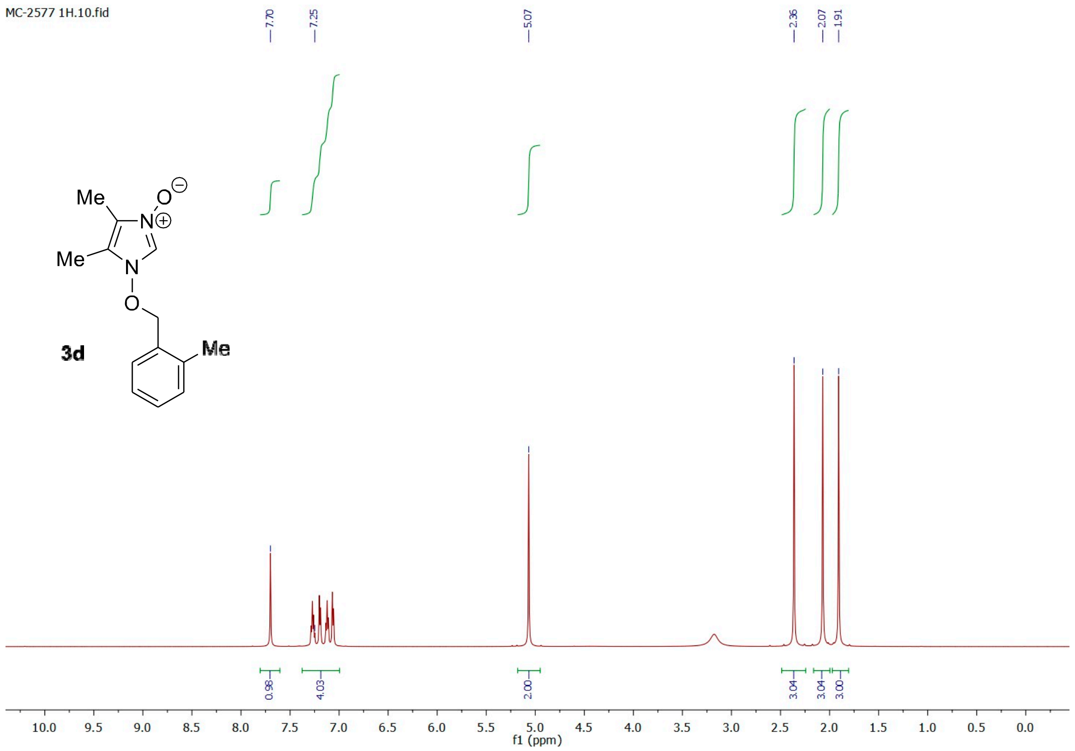

Figure S3. ^{1}H NMR of **3d** ($CDCl_3$, 600 MHz).

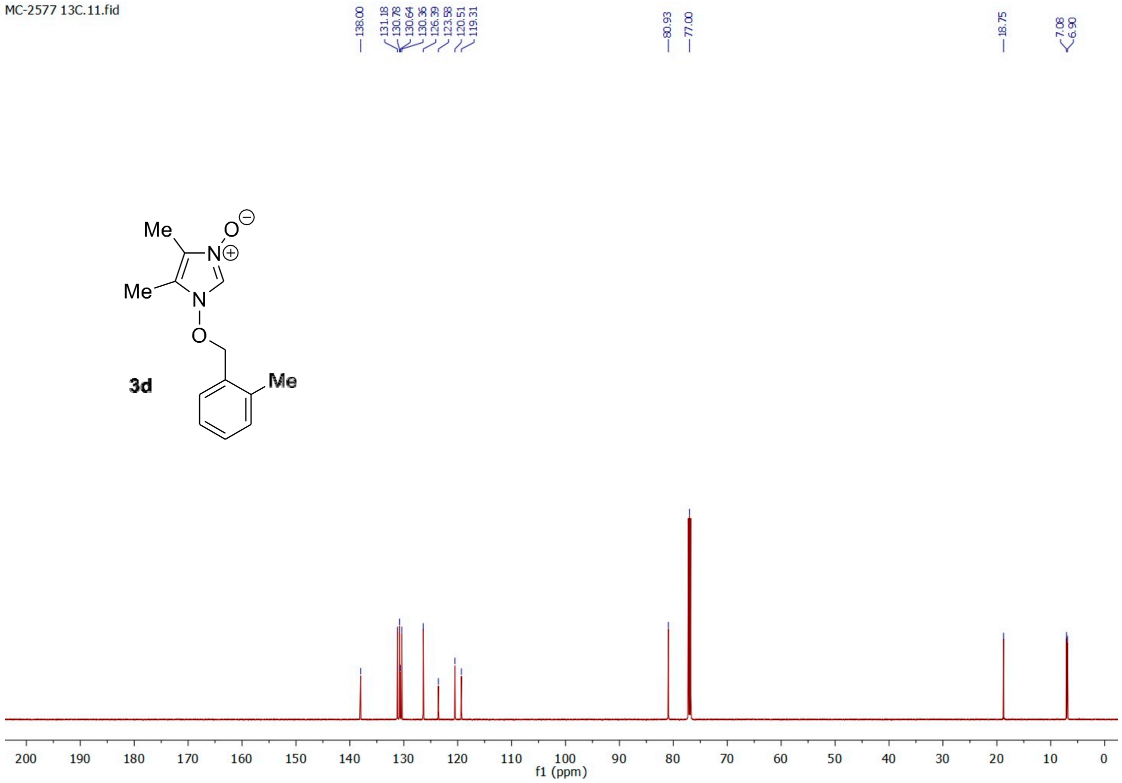

Figure S4. ^{13}C NMR of **3d** ($CDCl_3$, 151 MHz).

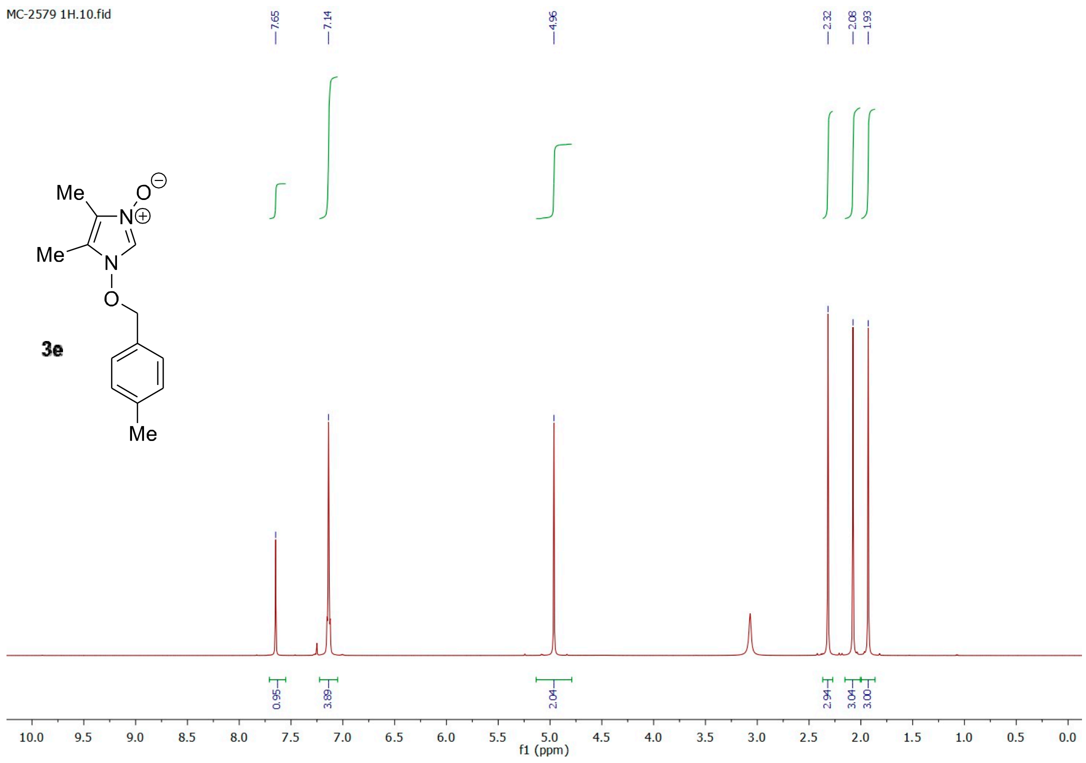

Figure S5. ^{1}H NMR of **3e** ($CDCl_3$, 600 MHz).

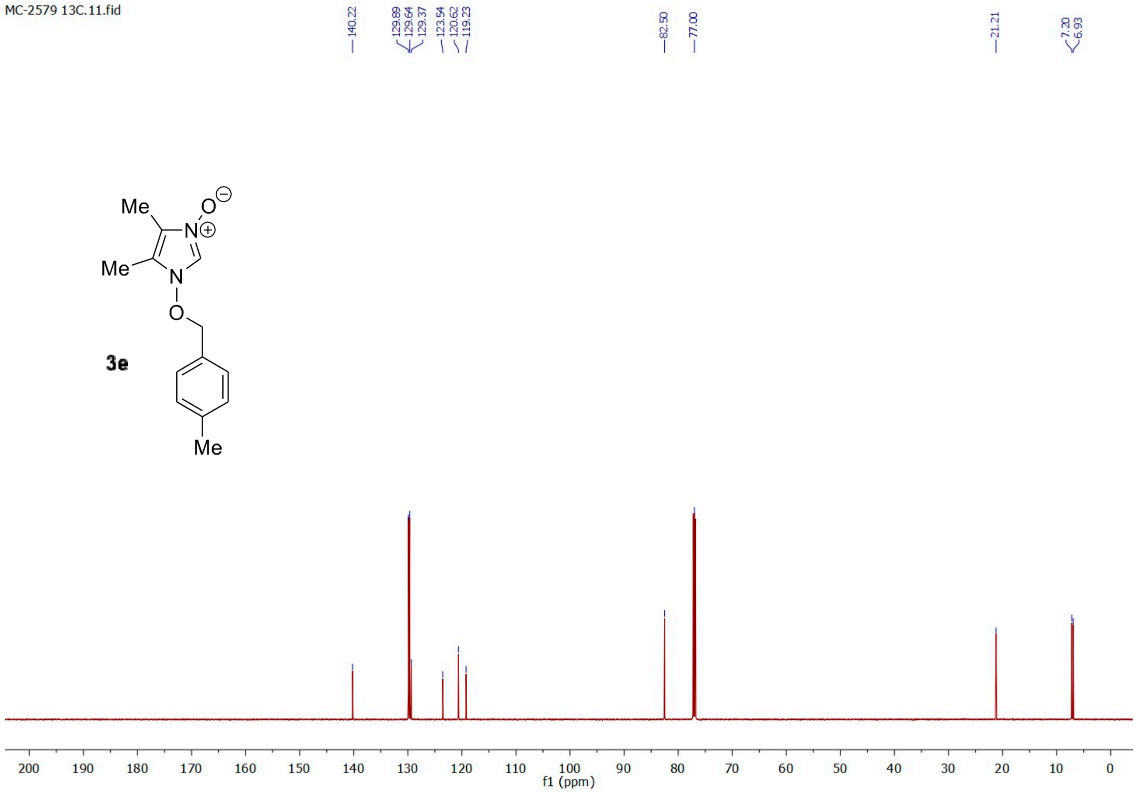

Figure S6. ^{13}C NMR of **3e** ($CDCl_3$, 151 MHz).

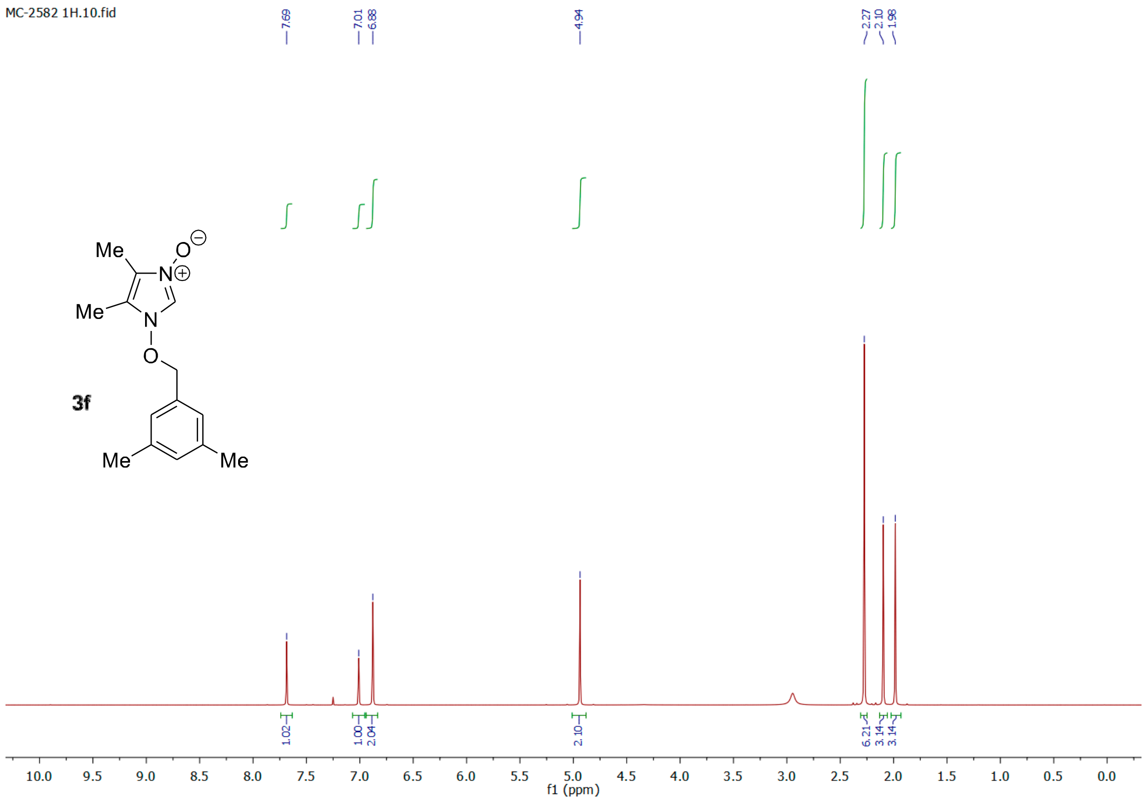

Figure S7. ^{1}H NMR of **3f** ($CDCl_3$, 600 MHz).

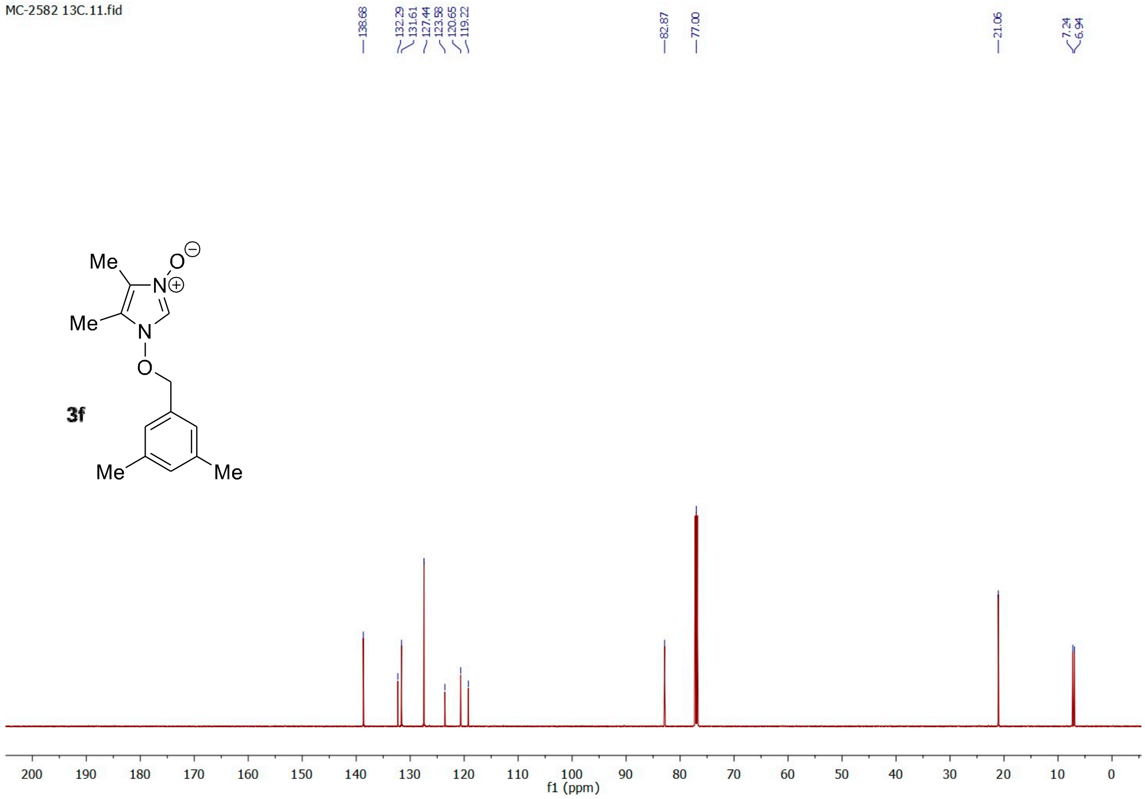

Figure S8. ^{13}C NMR of **3f** ($CDCl_3$, 151 MHz).

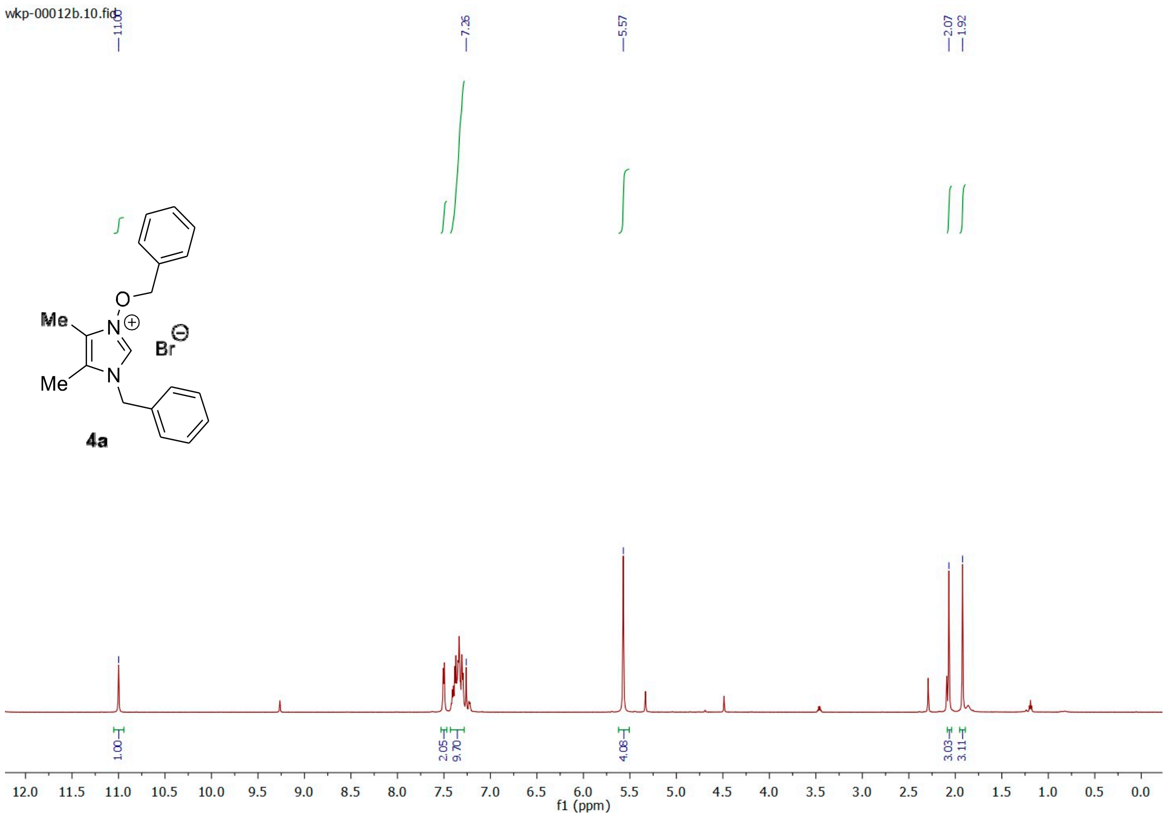

Figure S9. ^{1}H NMR of **4a** ($CDCl_3$, 600 MHz).

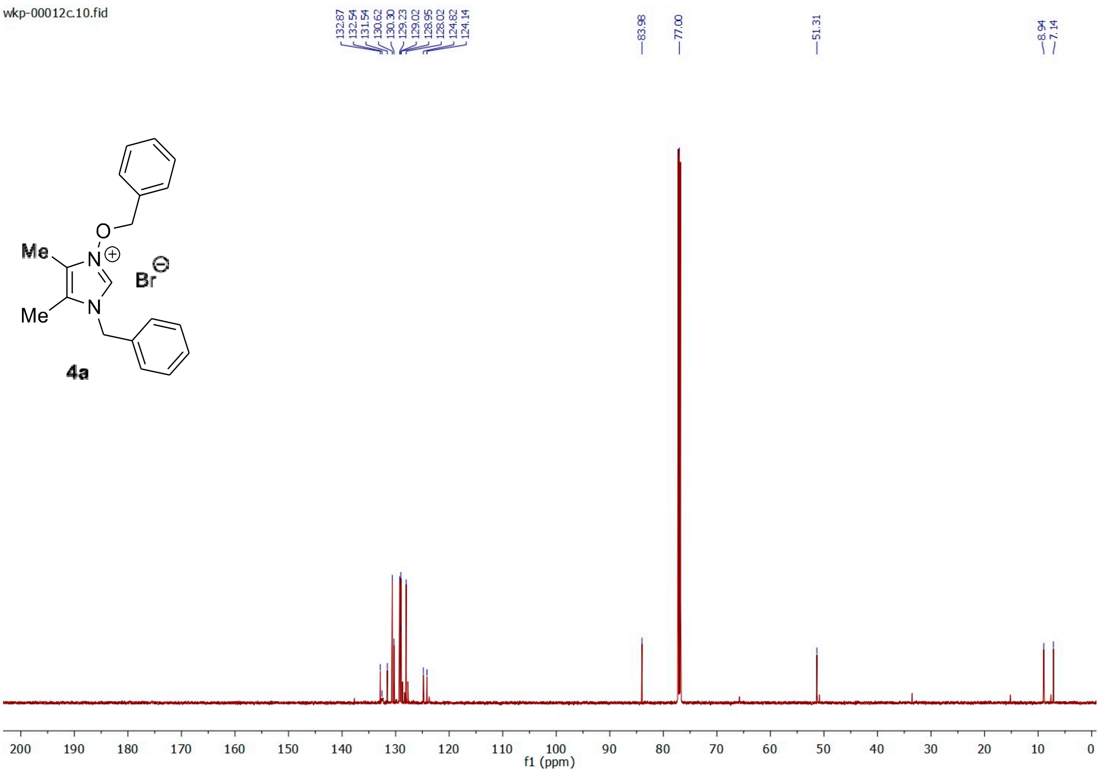

Figure S10. ^{13}C NMR of **4a** ($CDCl_3$, 151 MHz).

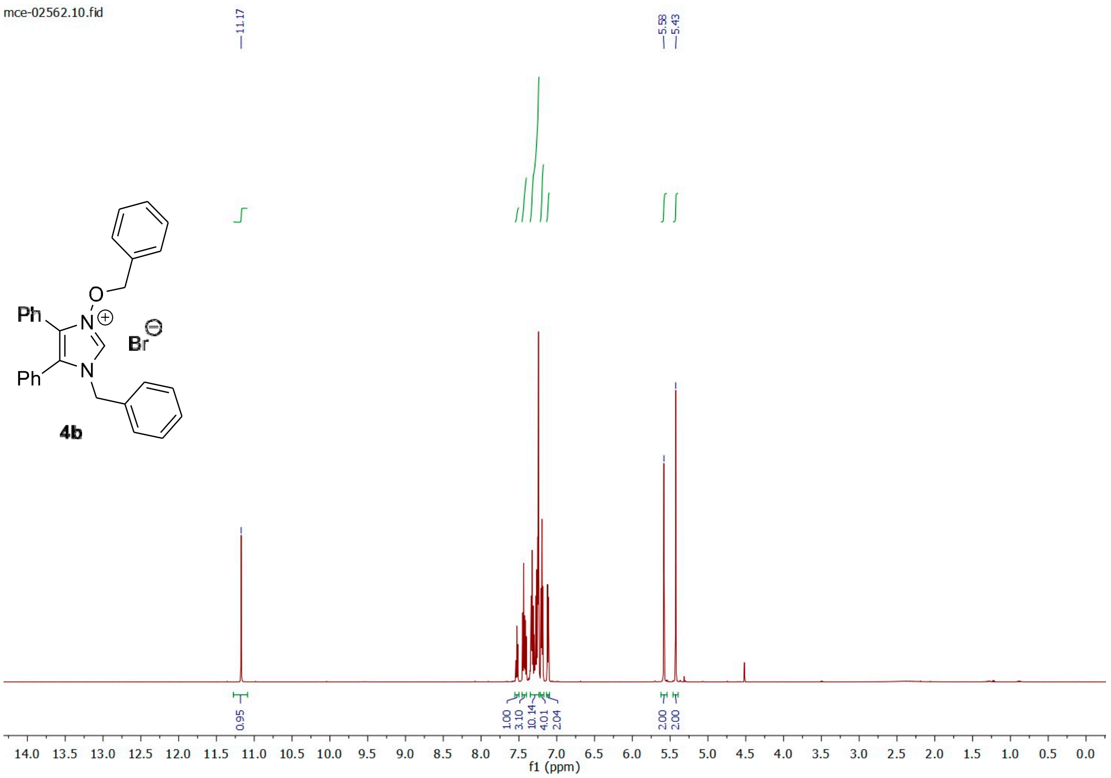

Figure S11. ^{1}H NMR of **4b** ($CDCl_3$, 600 MHz).

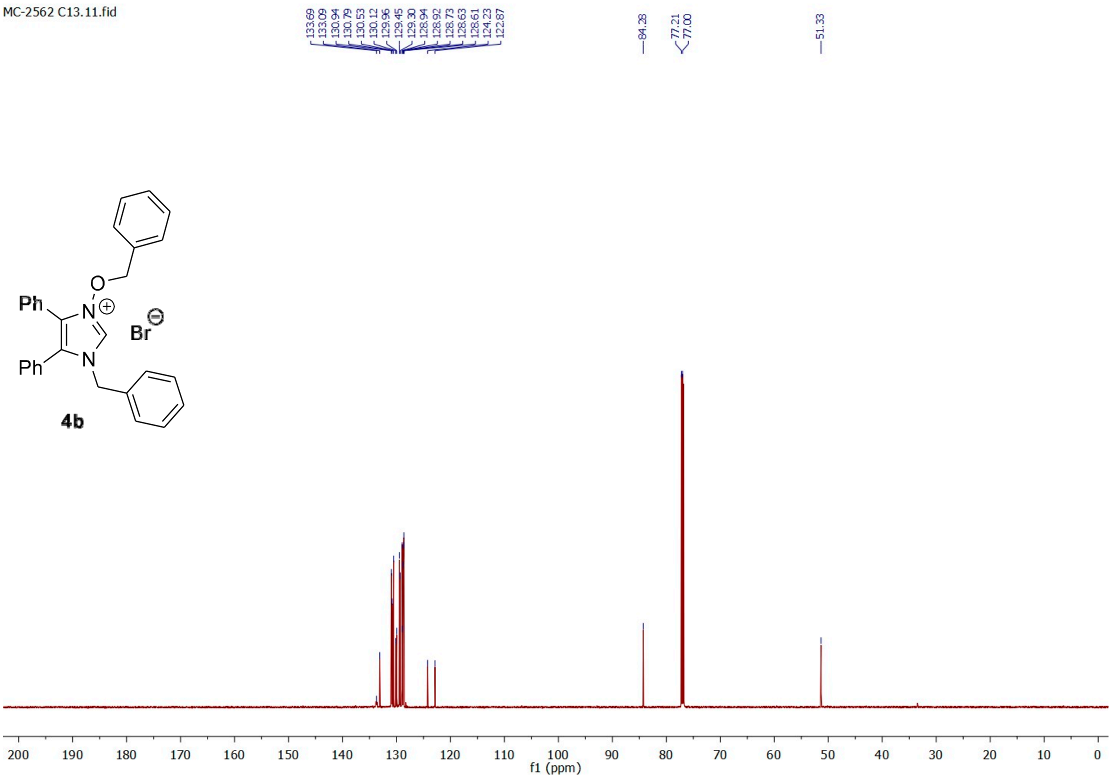

Figure S12. ^{13}C NMR of **4b** ($CDCl_3$, 151 MHz).

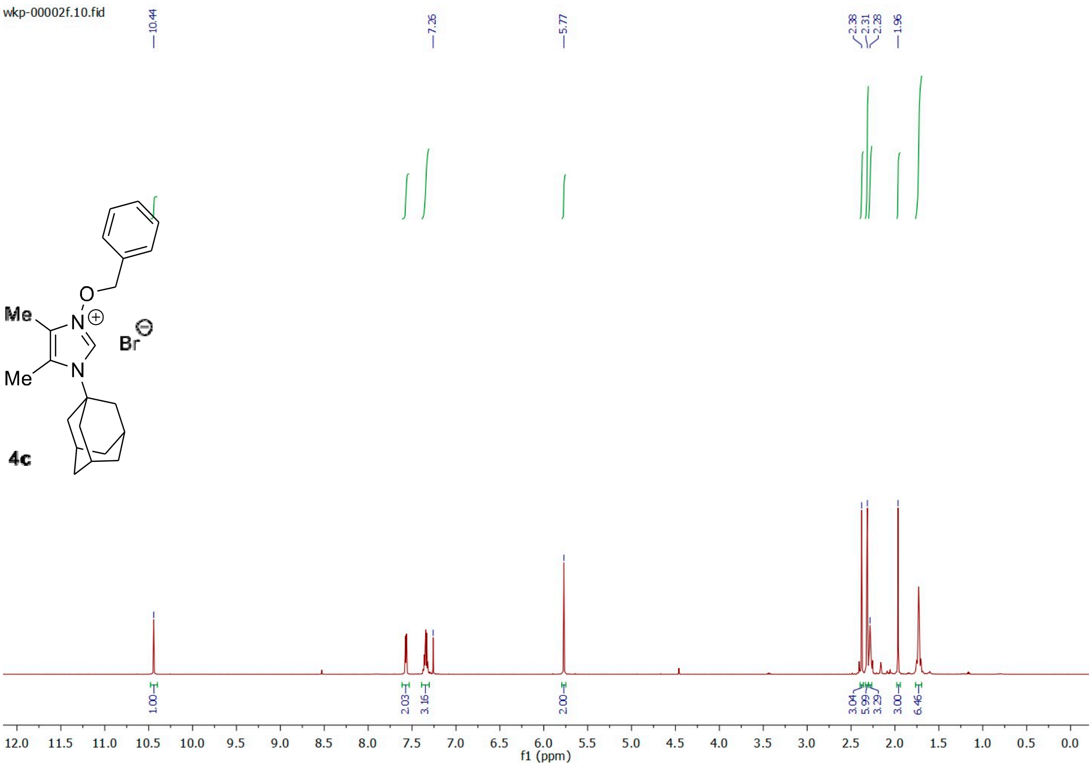

Figure S13. ^{1}H NMR of **4c** ($CDCl_3$, 600 MHz).

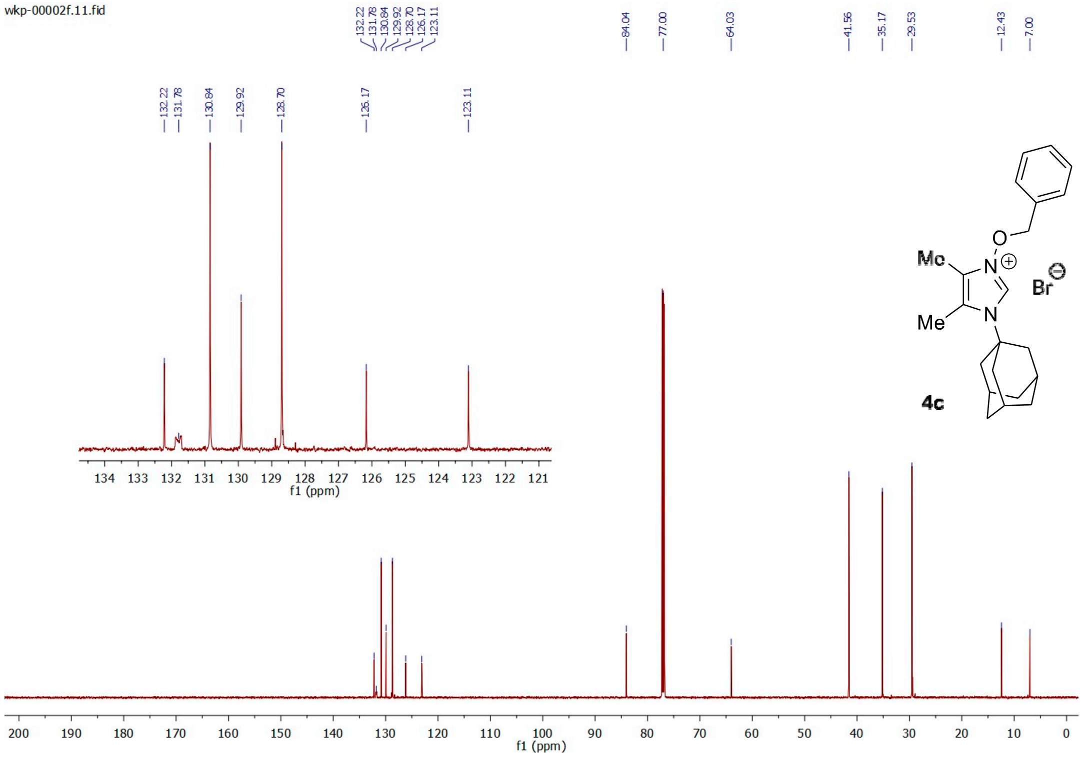

Figure S14. ^{13}C NMR of **4c** ($CDCl_3$, 151 MHz).

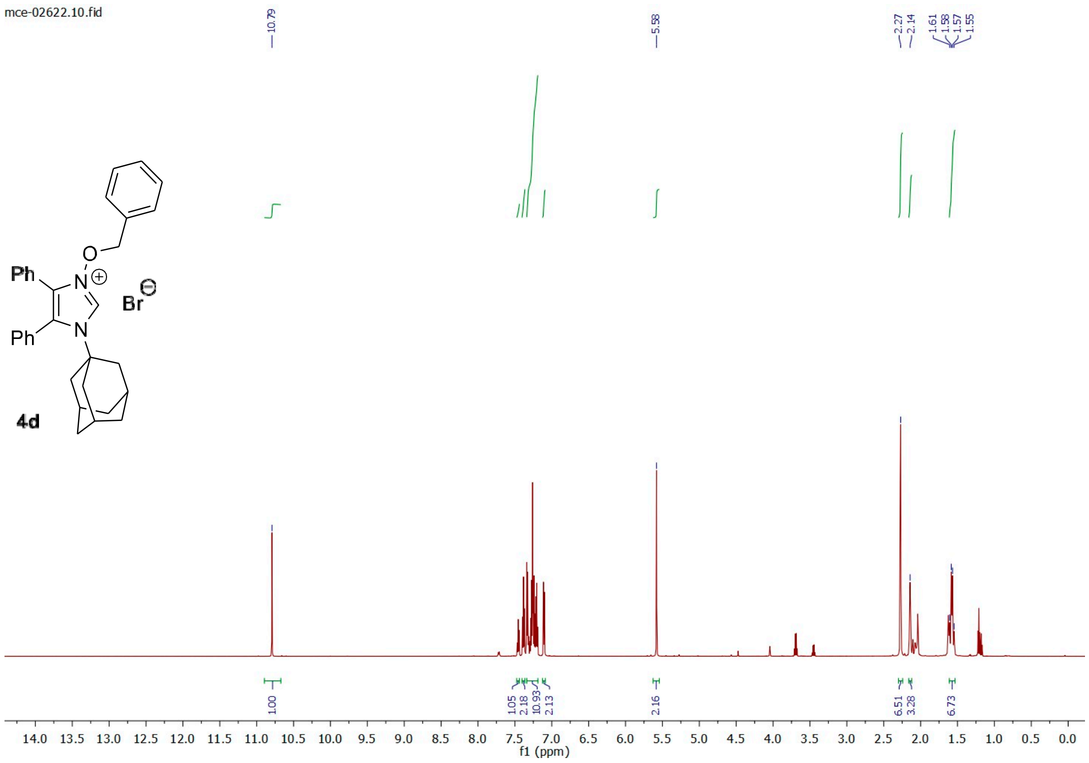

Figure S15. ^{1}H NMR of **4d** ($CDCl_3$, 600 MHz).

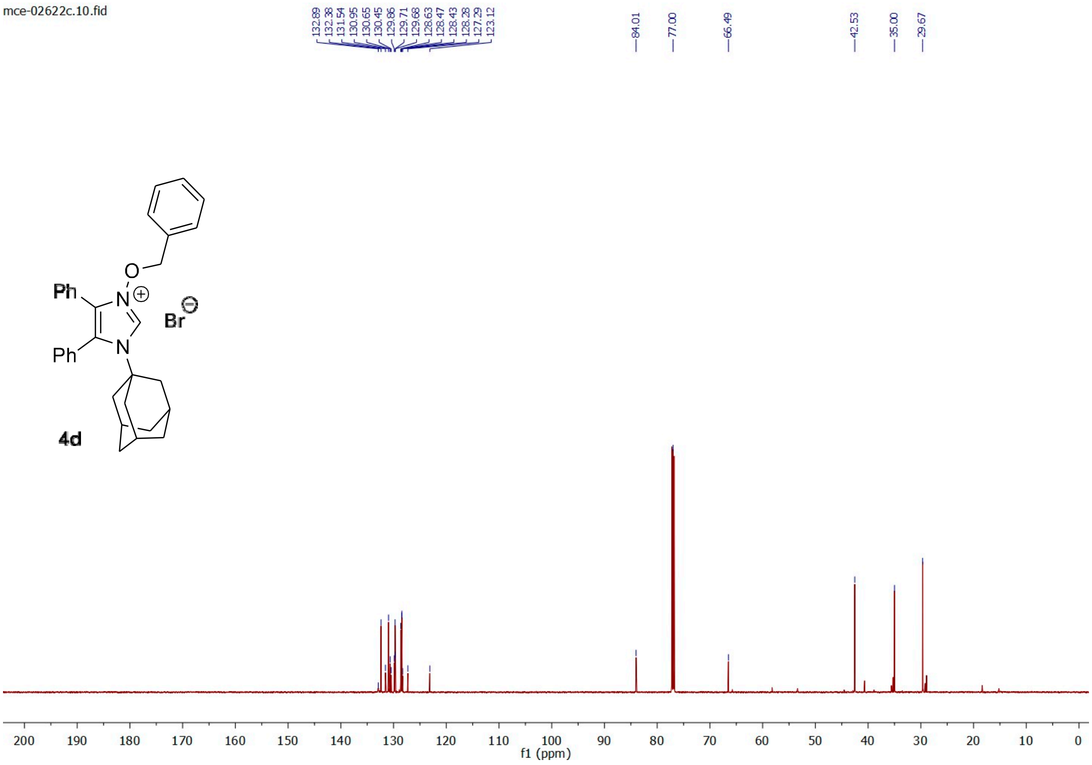

Figure S16. ^{13}C NMR of **4d** ($CDCl_3$, 151 MHz).

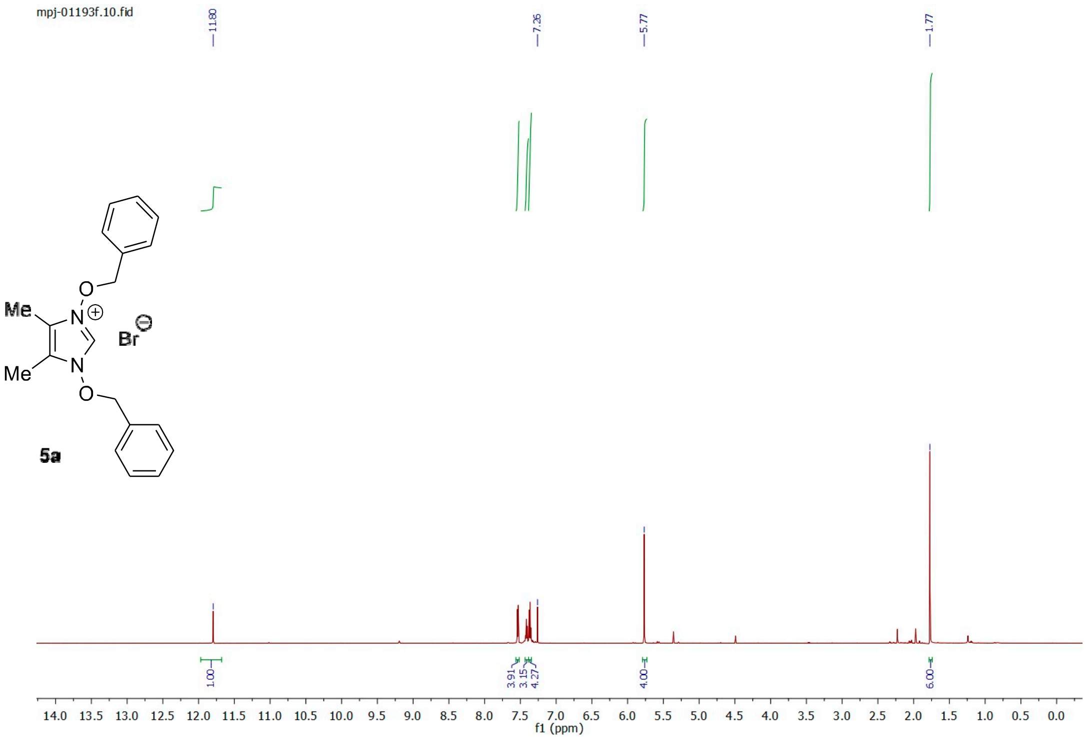

Figure S17. ^{1}H NMR of **5a** ($CDCl_3$, 600 MHz).

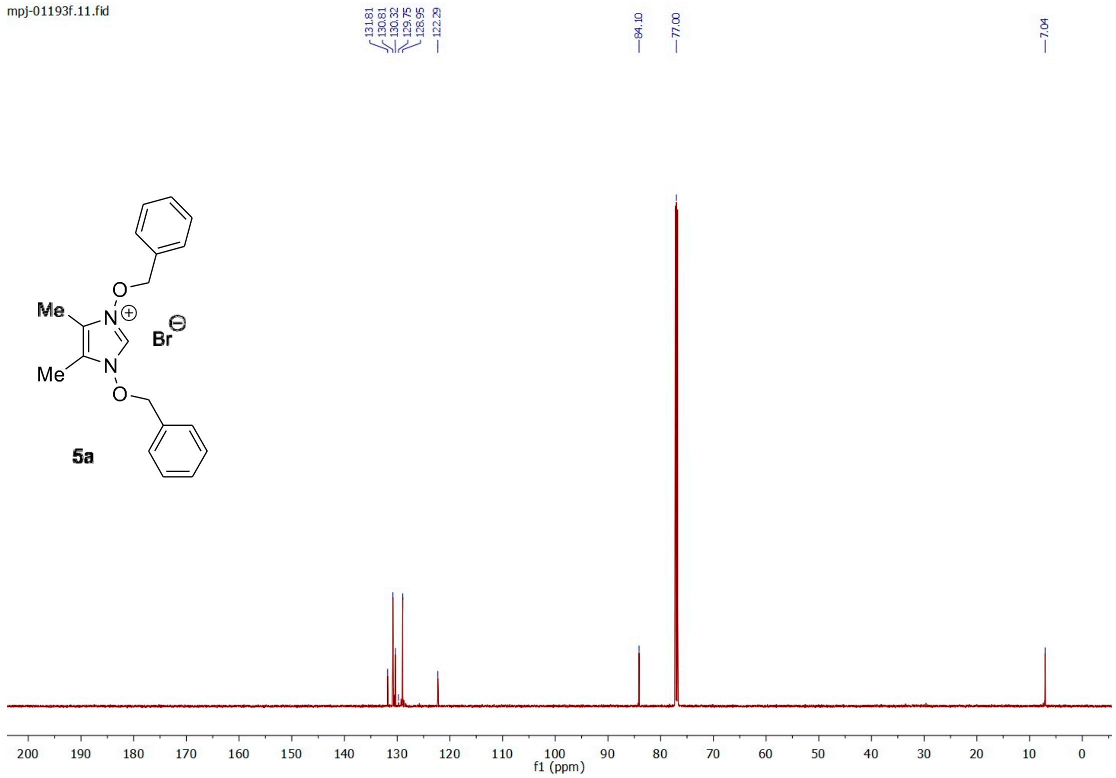

Figure S18. ^{13}C NMR of **5a** ($CDCl_3$, 151 MHz).

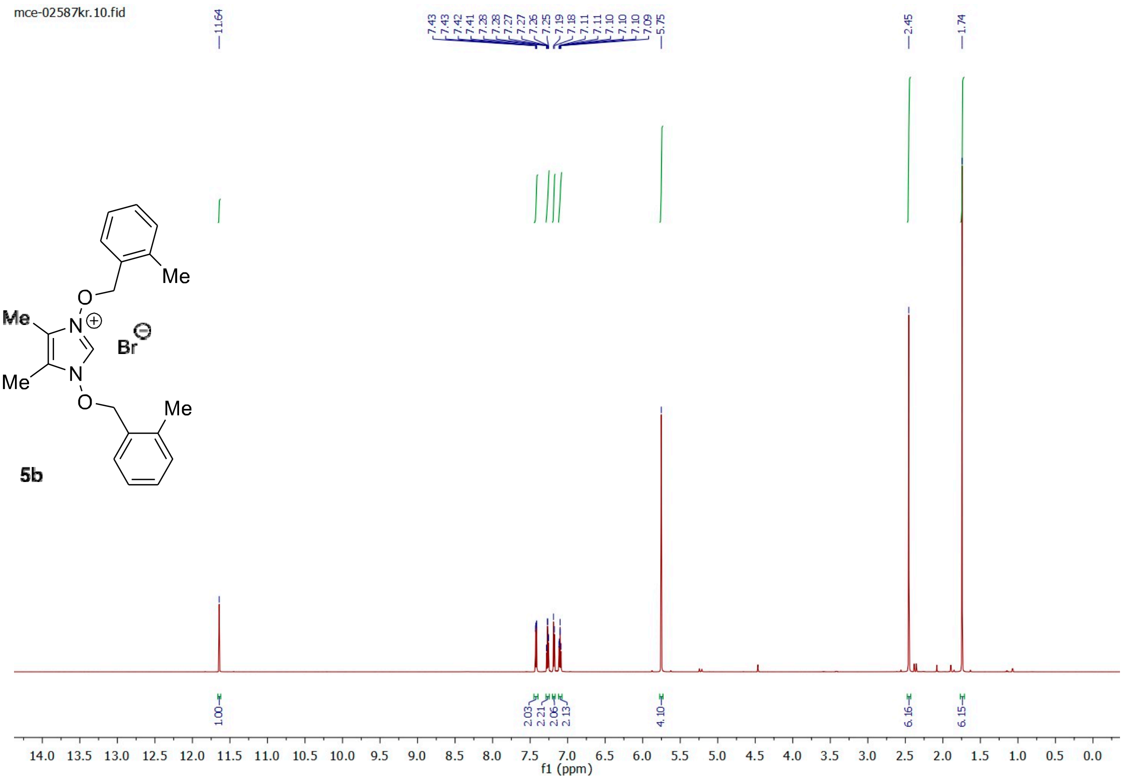

Figure S19. ^{1}H NMR of **5b** ($CDCl_3$, 600 MHz).

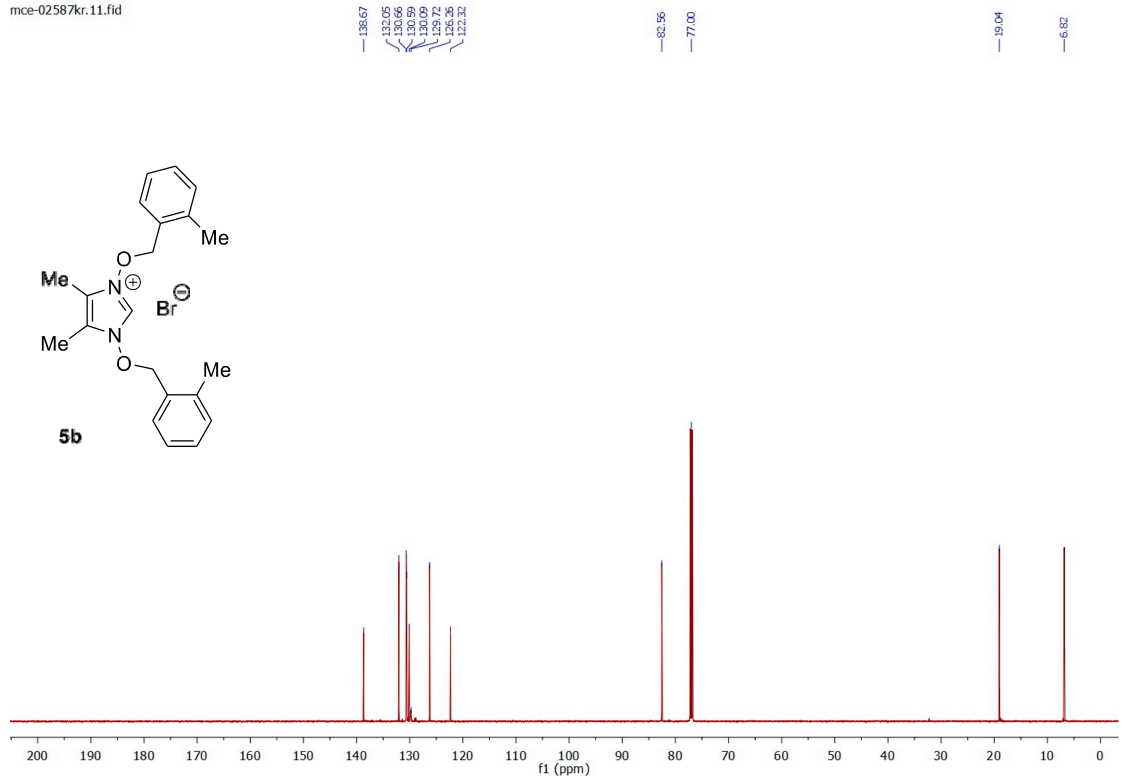

Figure S20. ^{13}C NMR of **5b** ($CDCl_3$, 151 MHz).

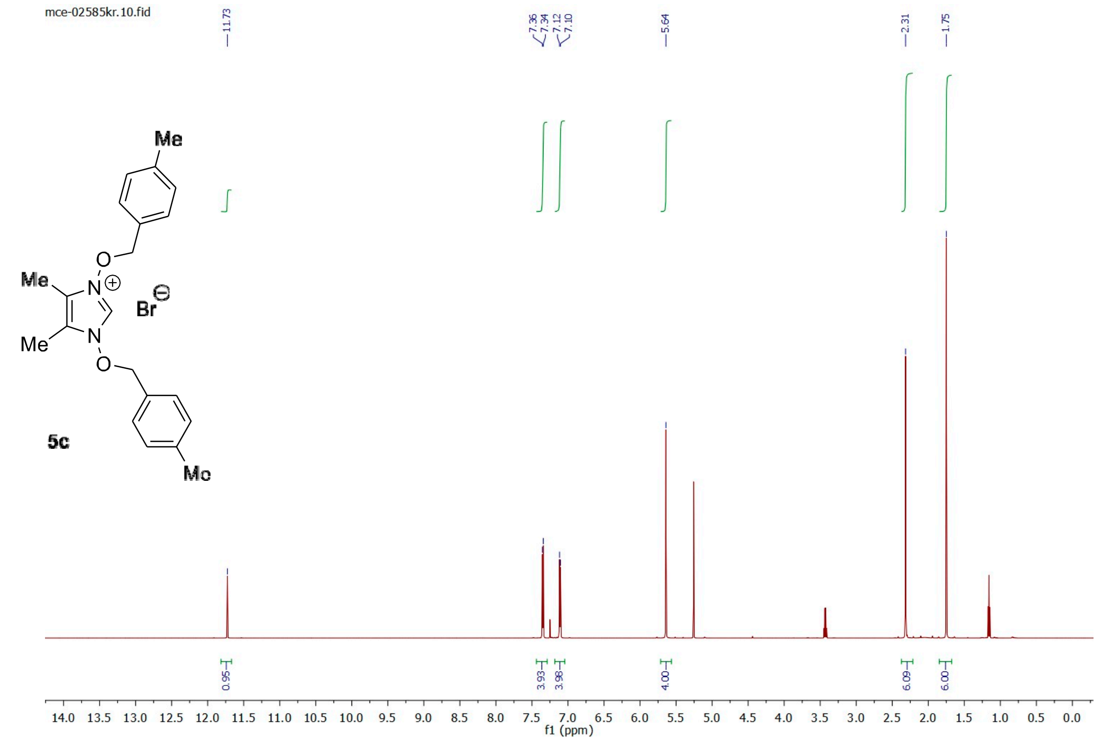

Figure S21. ¹H NMR of **5c** ($CDCl_3$, 600 MHz).

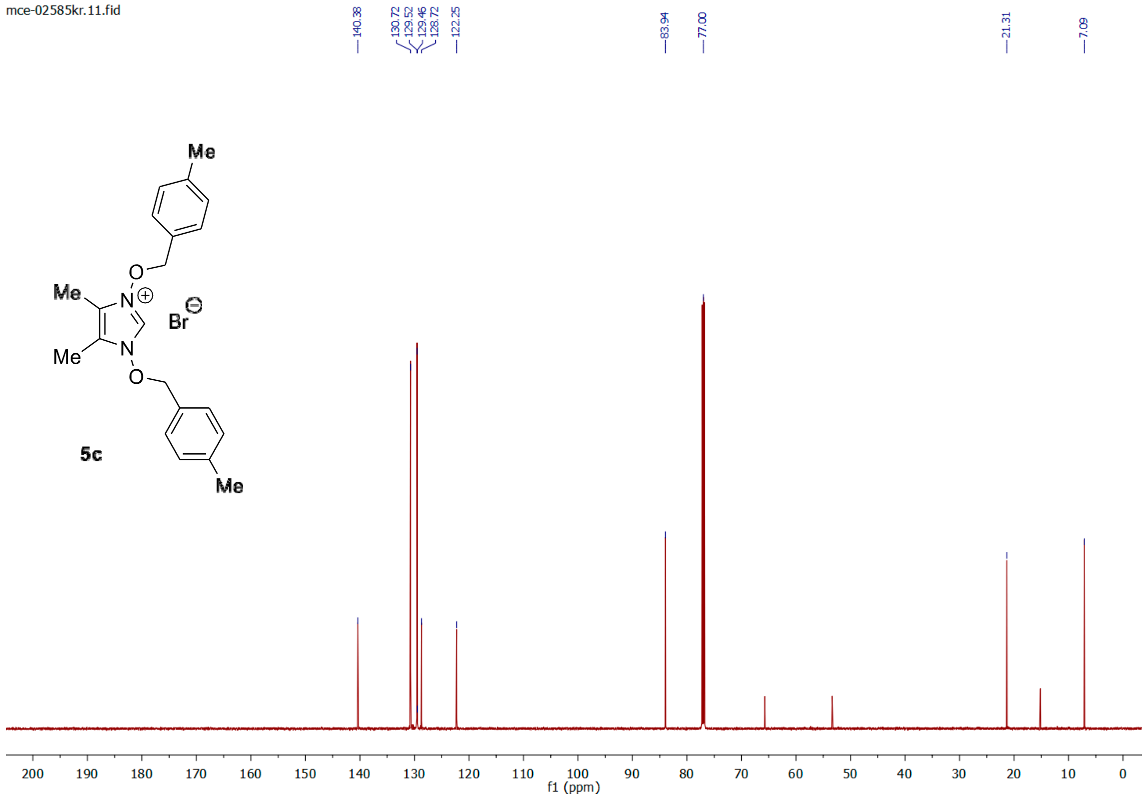

Figure S22. ¹³C NMR of **5c** ($CDCl_3$, 151 MHz).

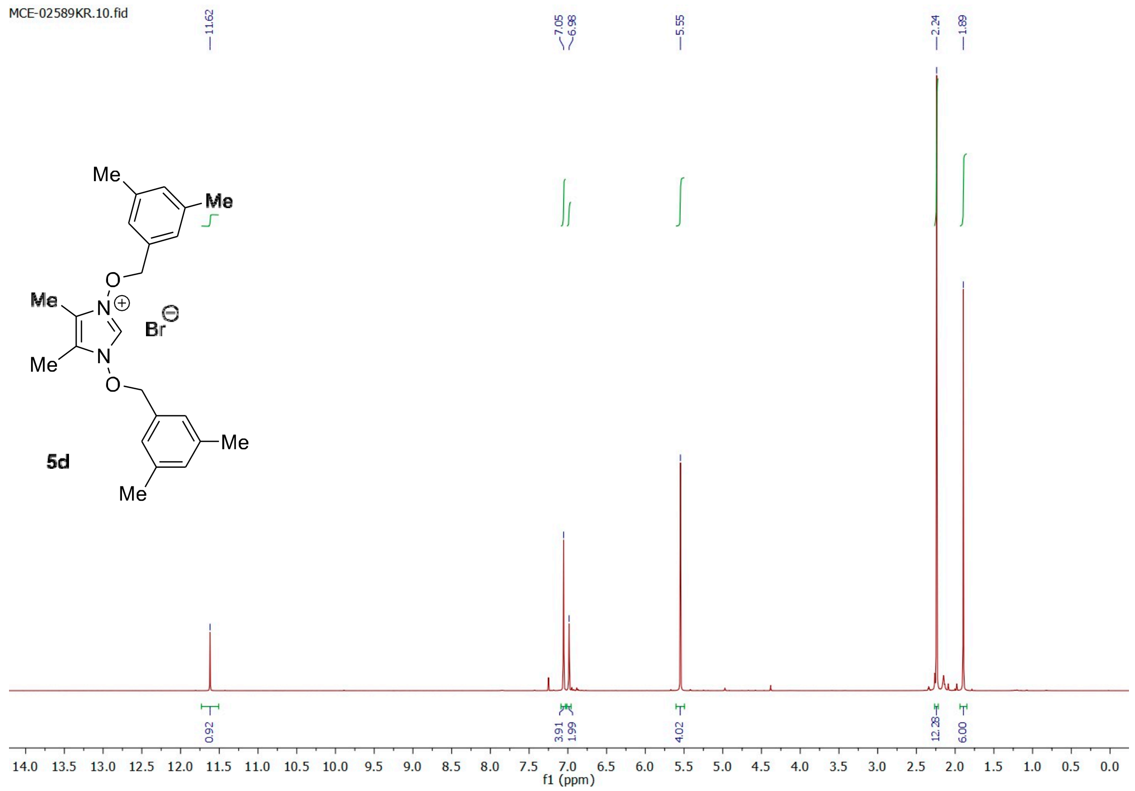

Figure S23. ^{1}H NMR of **5d** ($CDCl_3$, 600 MHz).

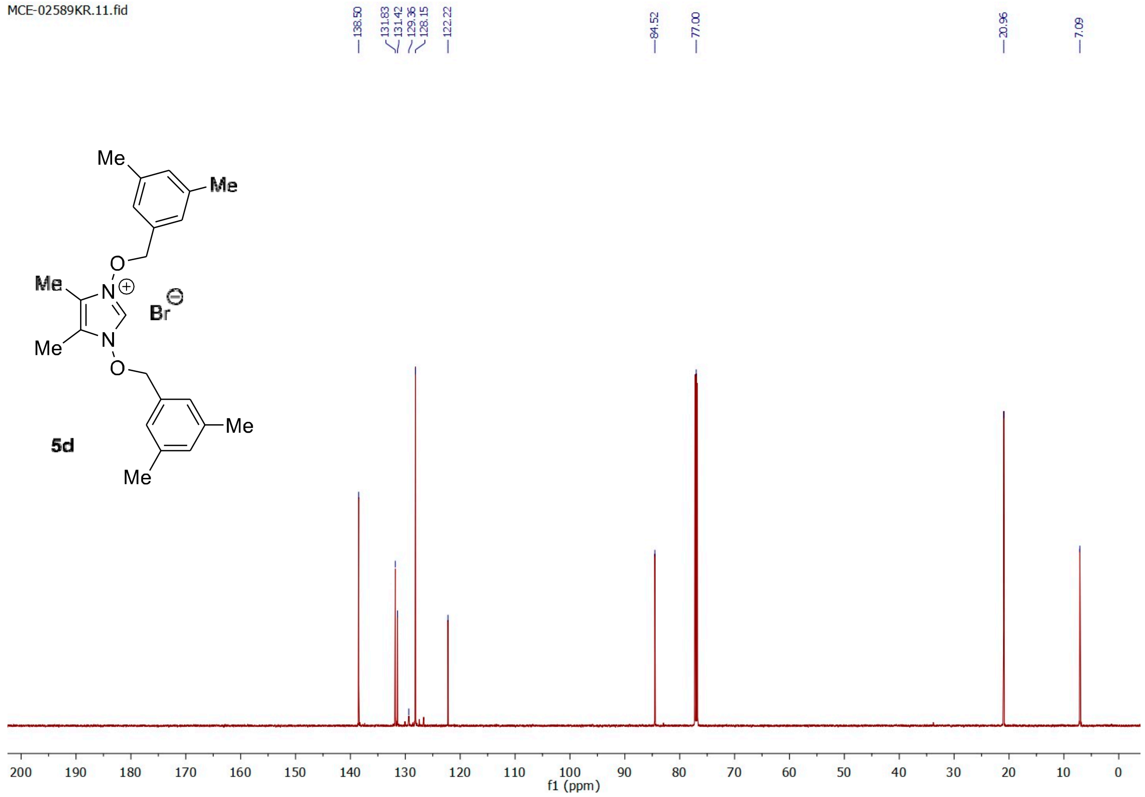

Figure S24. ^{13}C NMR of **5d** ($CDCl_3$, 151 MHz).

Bromide **5e** in a mixture with **5a** and **5b** (see the main text)

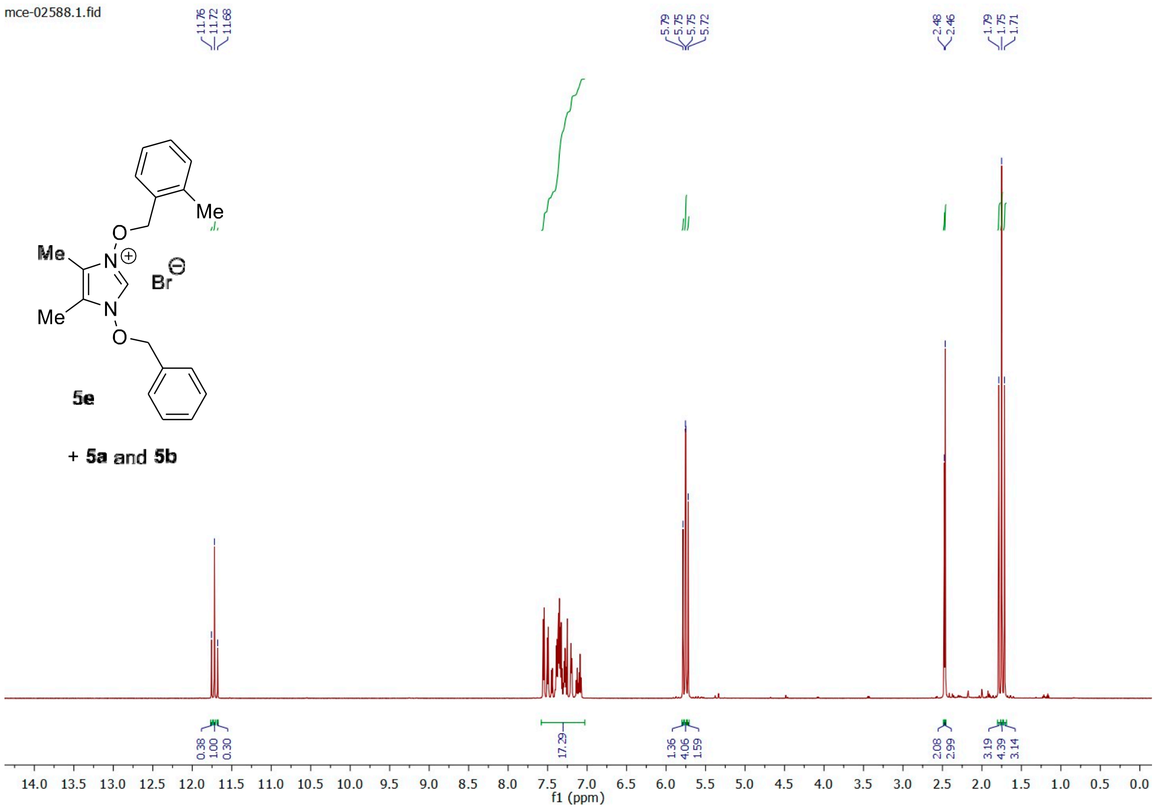

Figure S25. ^{1}H NMR ($CDCl_3$, 600 MHz) of a mixture of **5e**, **5a** and **5b** in a *ca*. 4:3:10 ratio obtained by treatment of imidazole *N*-oxide **3d** with benzyl bromide (CH_2Cl_2, rt, overnight).

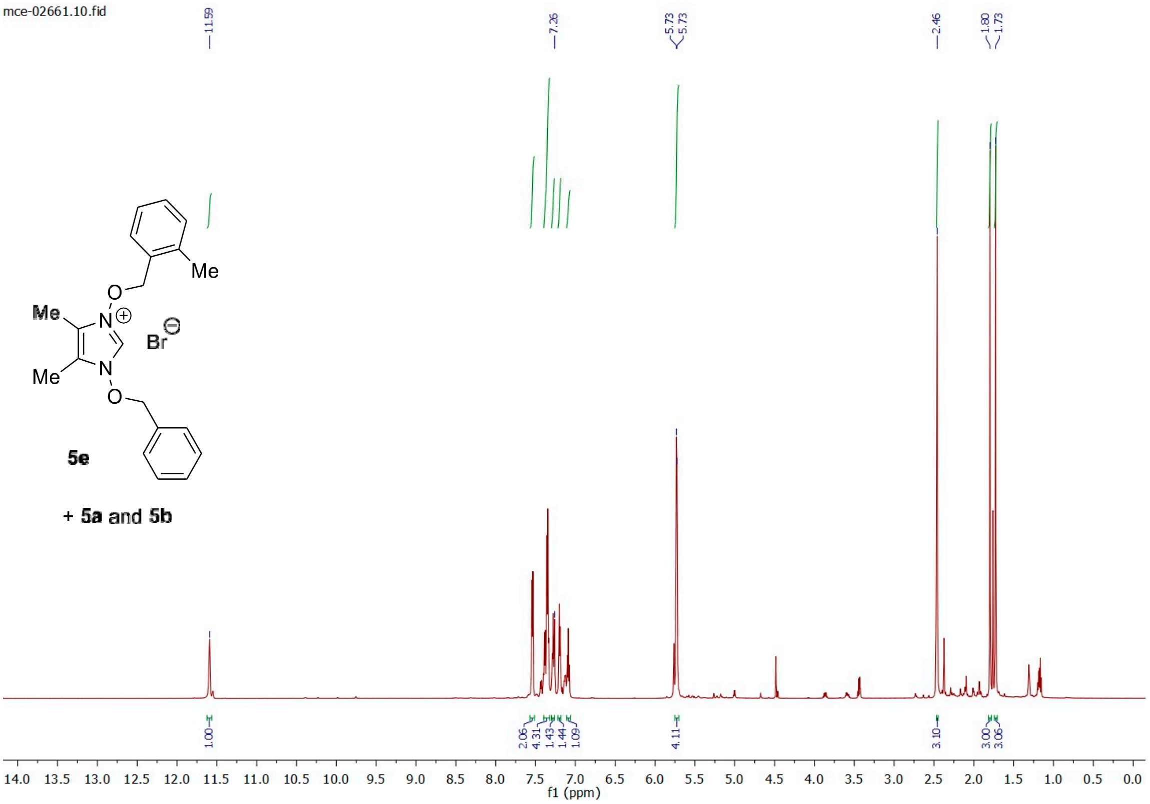

Figure S26. ^{1}H NMR ($CDCl_3$, 600 MHz) of a mixture of **5e**, **5a** and **5b** in a *ca*. 20:1:1 ratio obtained by treatment of imidazole *N*-oxide **3c** with 2-methylbenzyl bromide (CH_2Cl_2, rt, overnight).

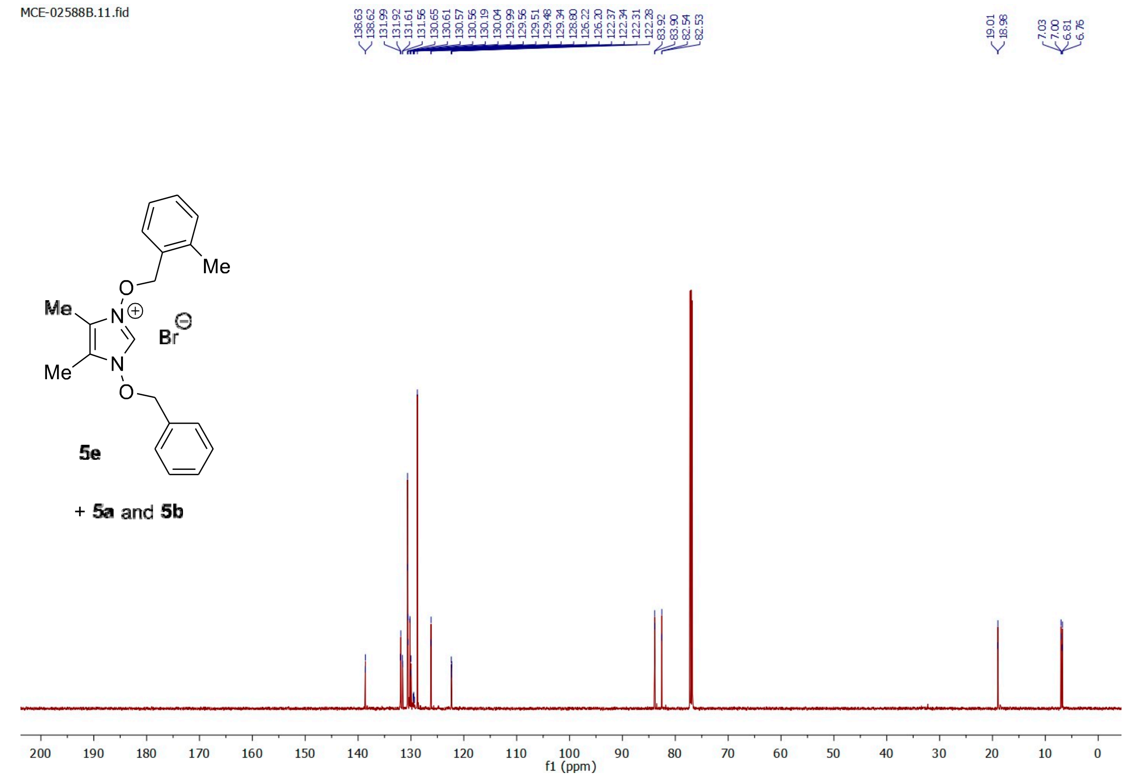

Figure S27. ^{13}C NMR of a mixture of **5e**, **5a** and **5b** in a *ca*. 4:3:10 ratio ($CDCl_3$, 151 MHz).

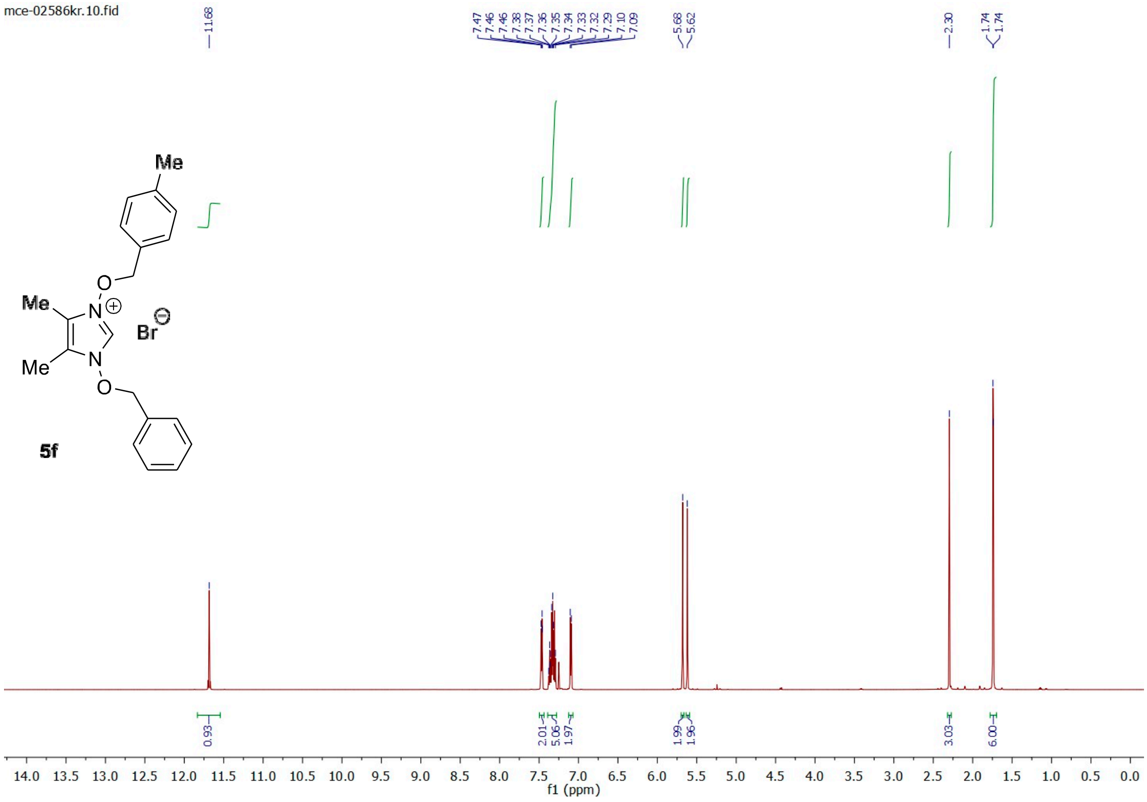

Figure S28. ^{1}H NMR of **5f** ($CDCl_3$, 600 MHz).

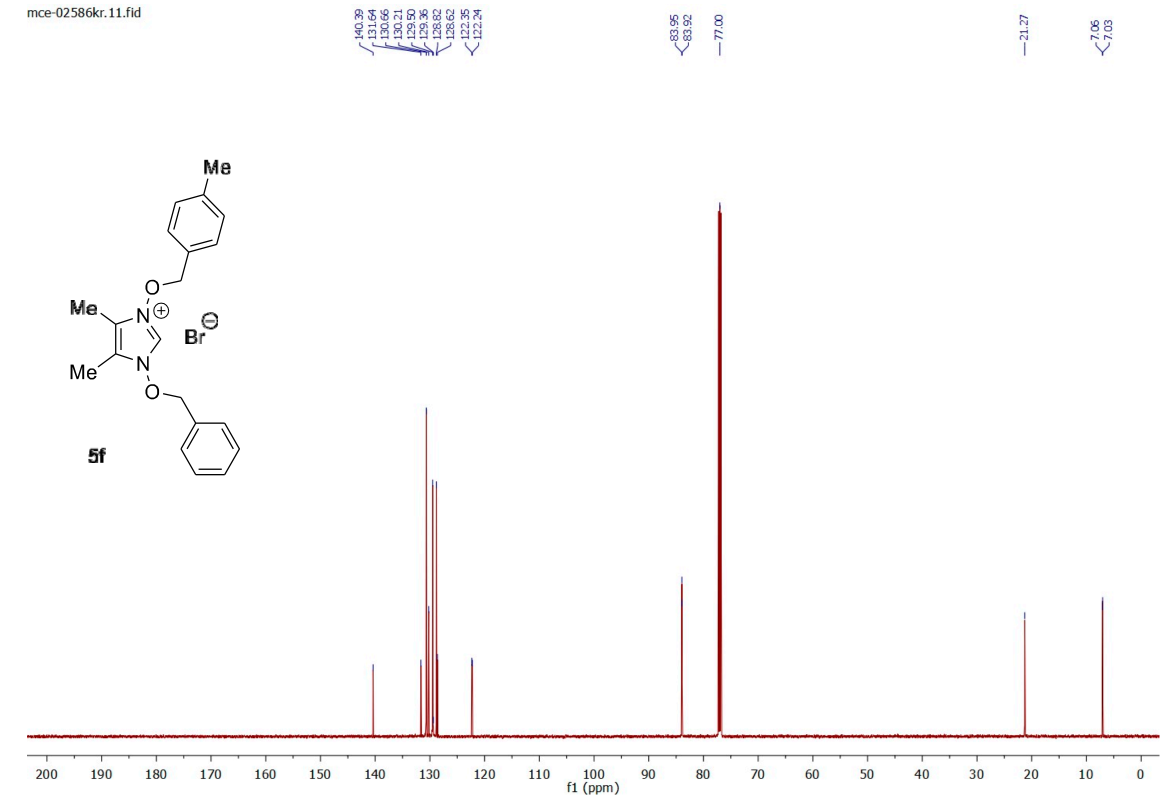

Figure S29. ^{13}C NMR of **5f** ($CDCl_3$, 151 MHz)..

Bromide **5g** in a mixture with *ca*. 10% of **5d** (see the main text)

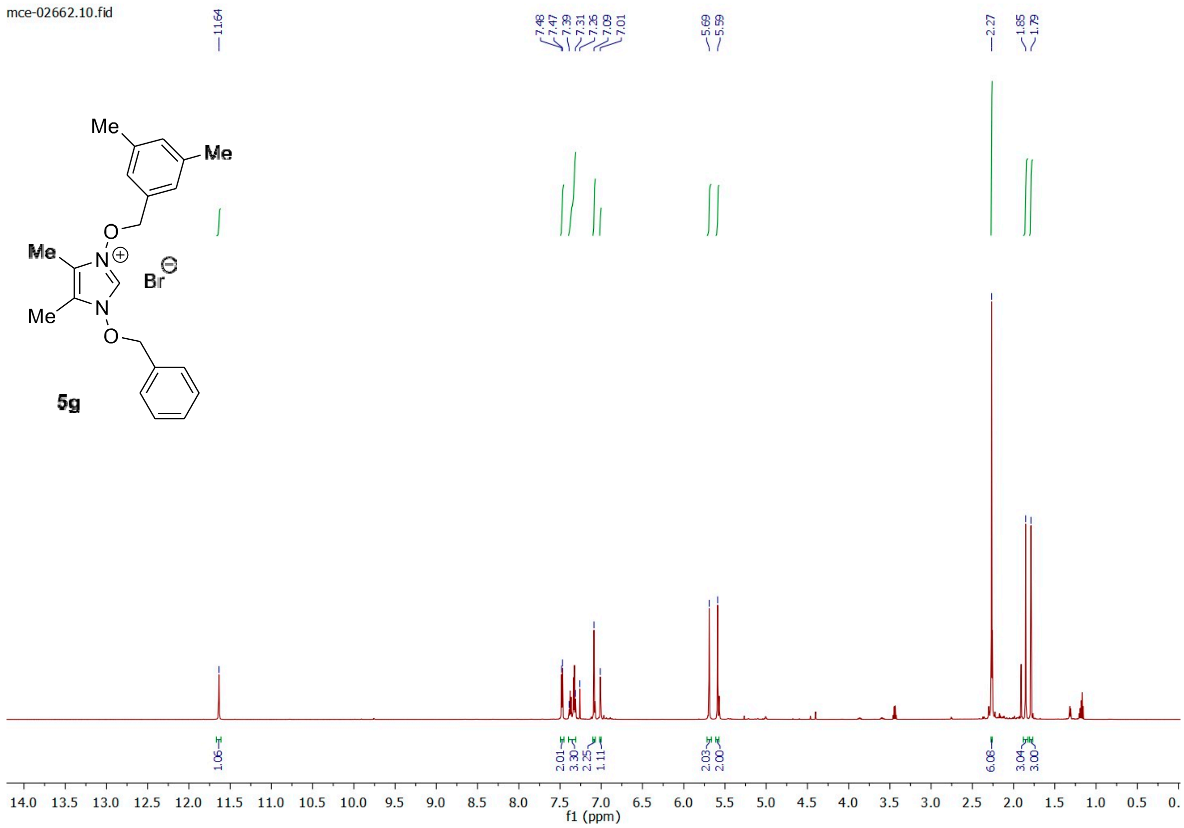

Figure S30. ^{1}H NMR of **5g** ($CDCl_3$, 600 MHz).

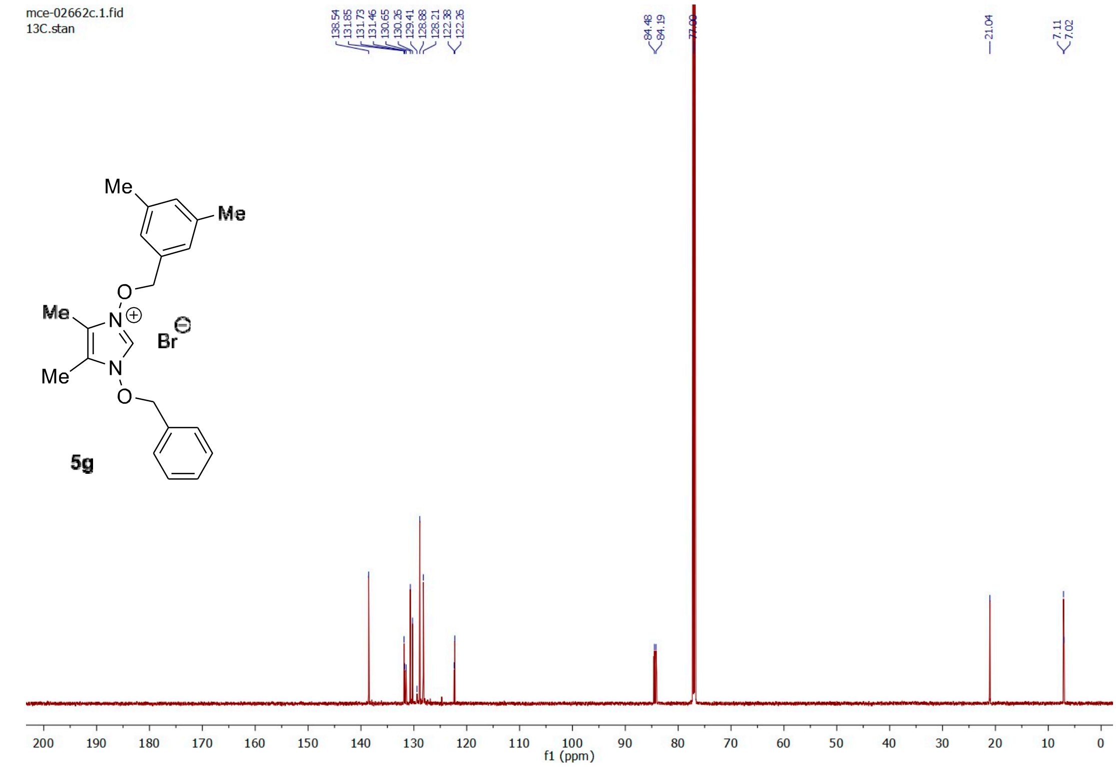

Figure S31. ^{13}C NMR of **5g** ($CDCl_3$, 151 MHz).

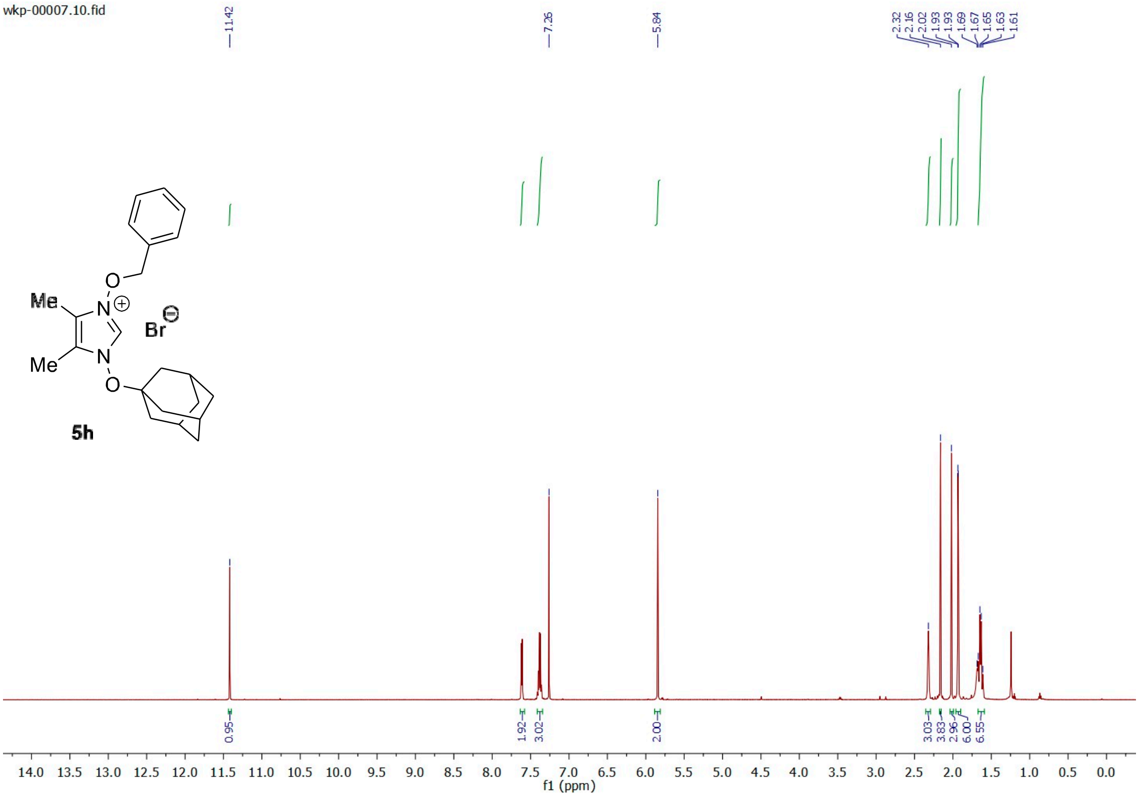

Figure S32. ^{1}H NMR of **5h** ($CDCl_3$, 600 MHz).

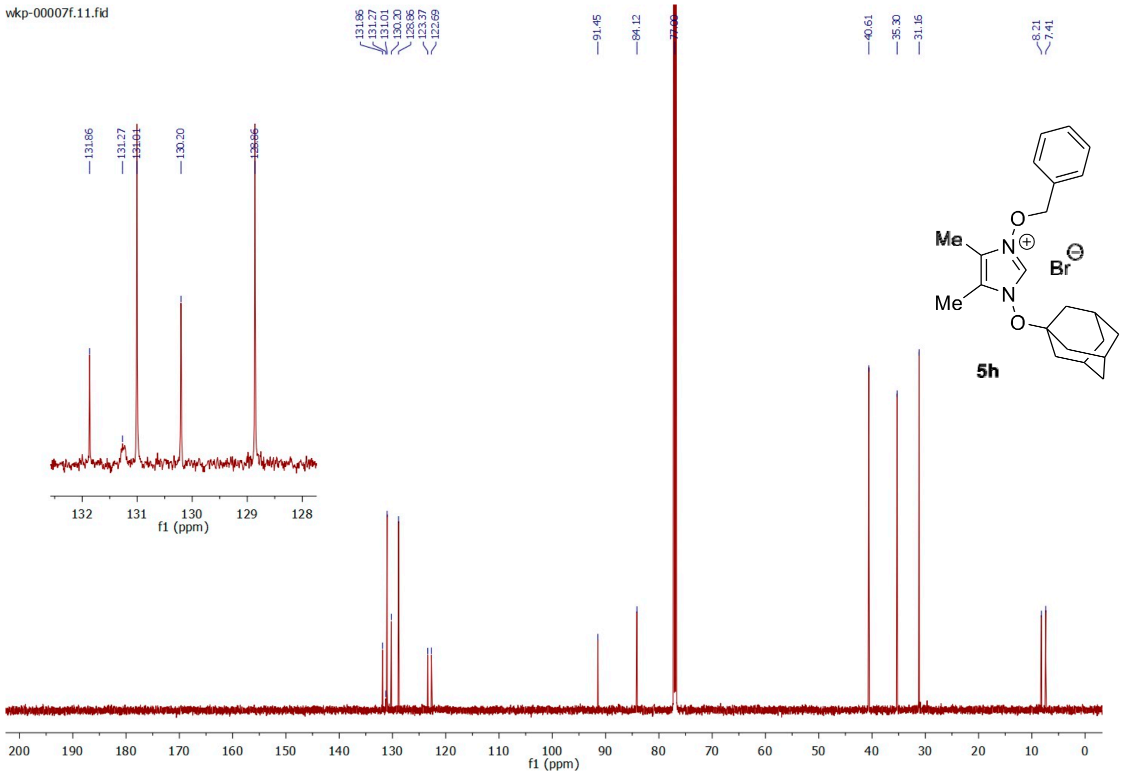

Figure S33. ^{13}C NMR of **5h** ($CDCl_3$, 151 MHz).

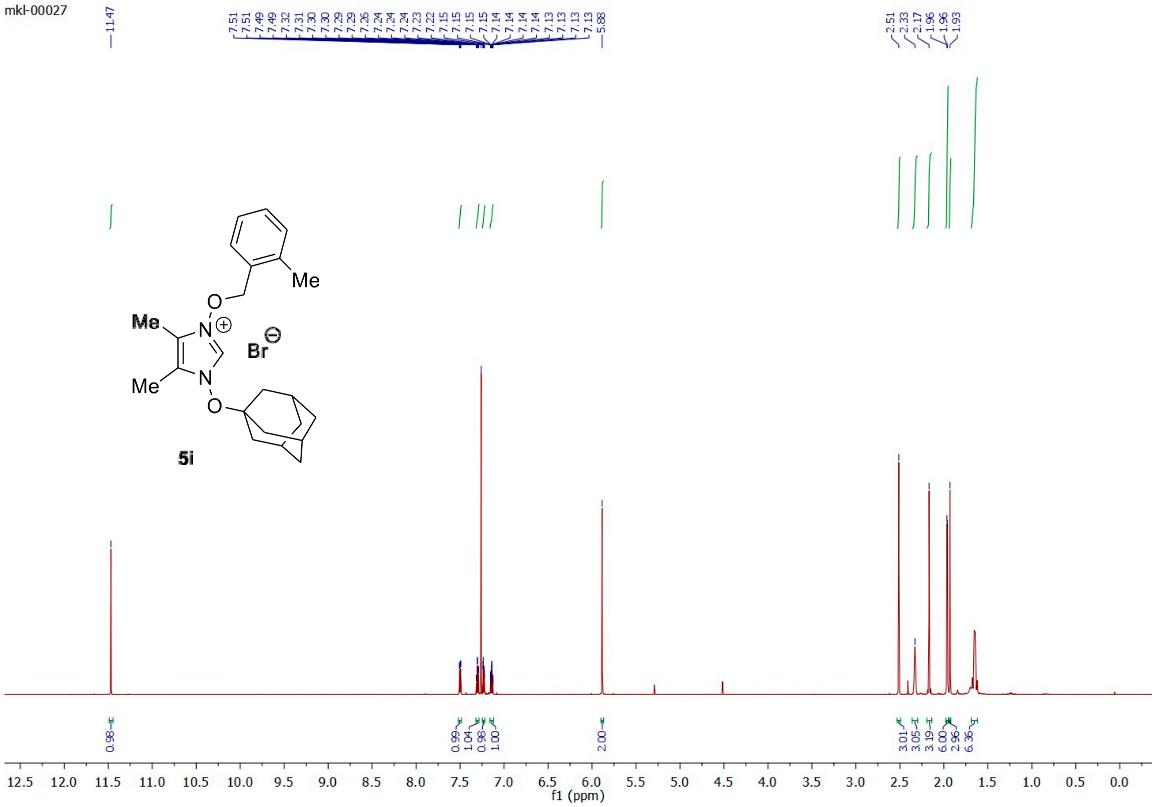

Figure S34. ^{1}H NMR of **5i** ($CDCl_3$, 600 MHz).

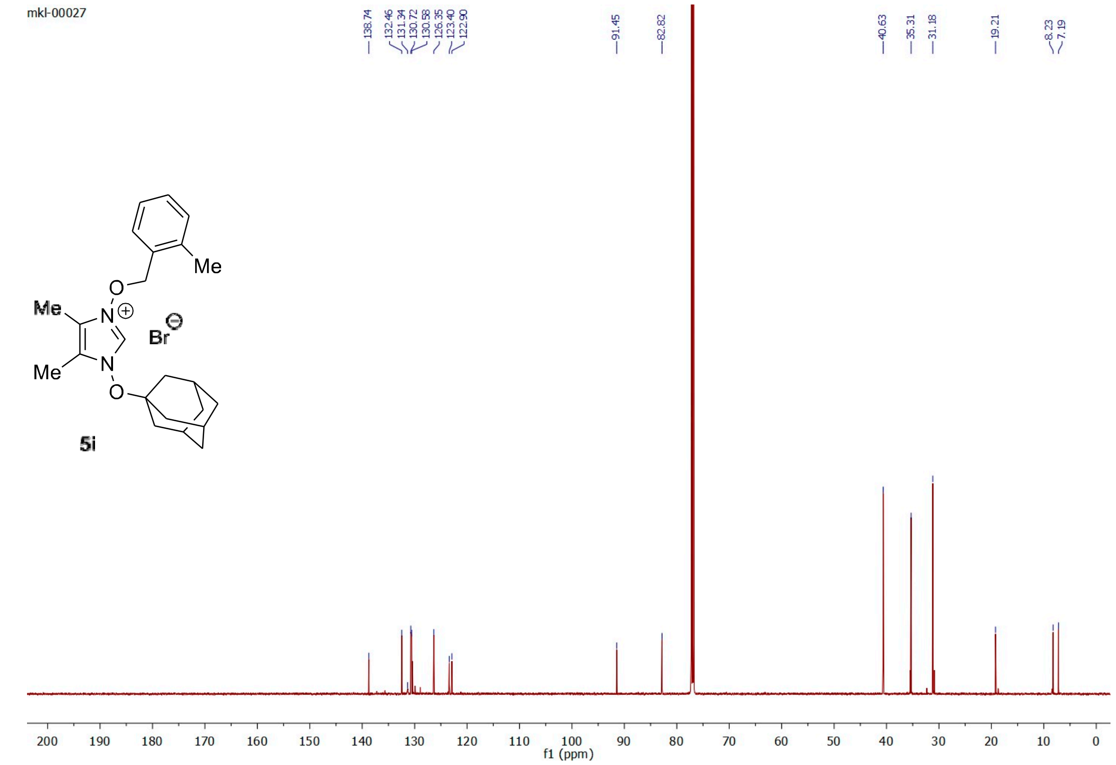

Figure S35. ^{13}C NMR of **5i** ($CDCl_3$, 151 MHz).

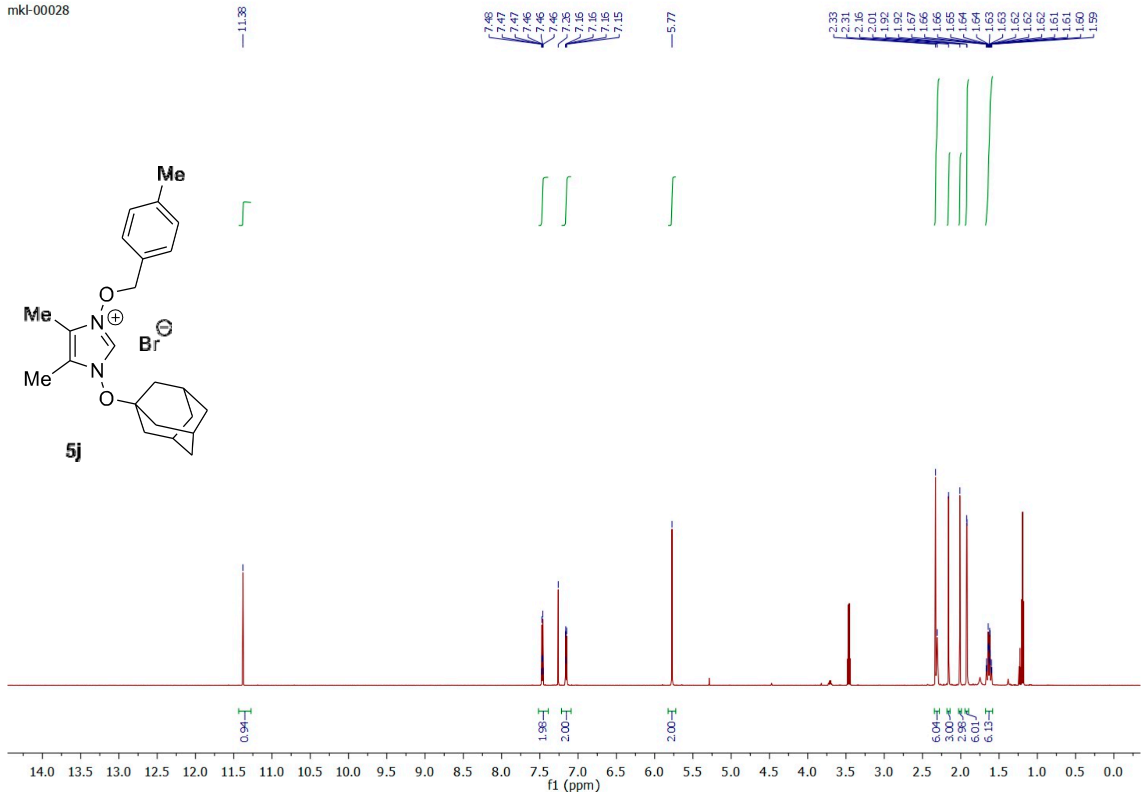

Figure S36. ^{1}H NMR of **5j** ($CDCl_3$, 600 MHz).

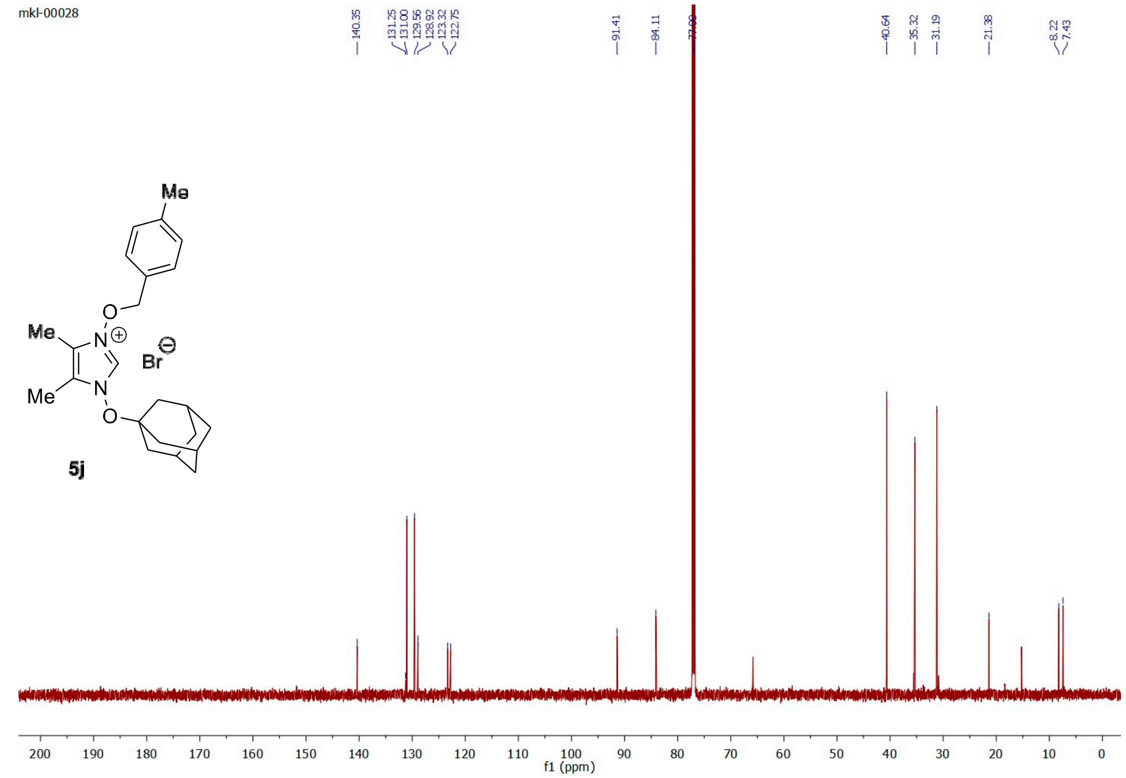

Figure S37. ^{13}C NMR of **5j** ($CDCl_3$, 151 MHz).

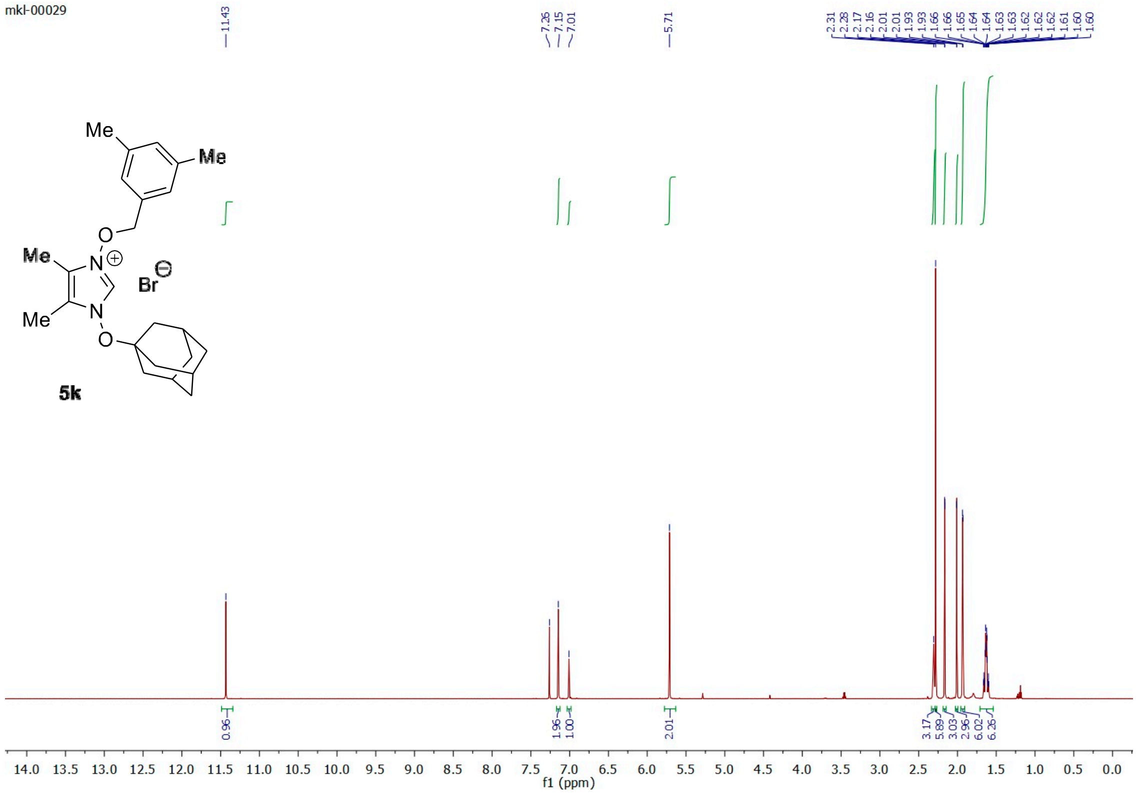

Figure S38. ^{1}H NMR of **5k** ($CDCl_3$, 600 MHz).

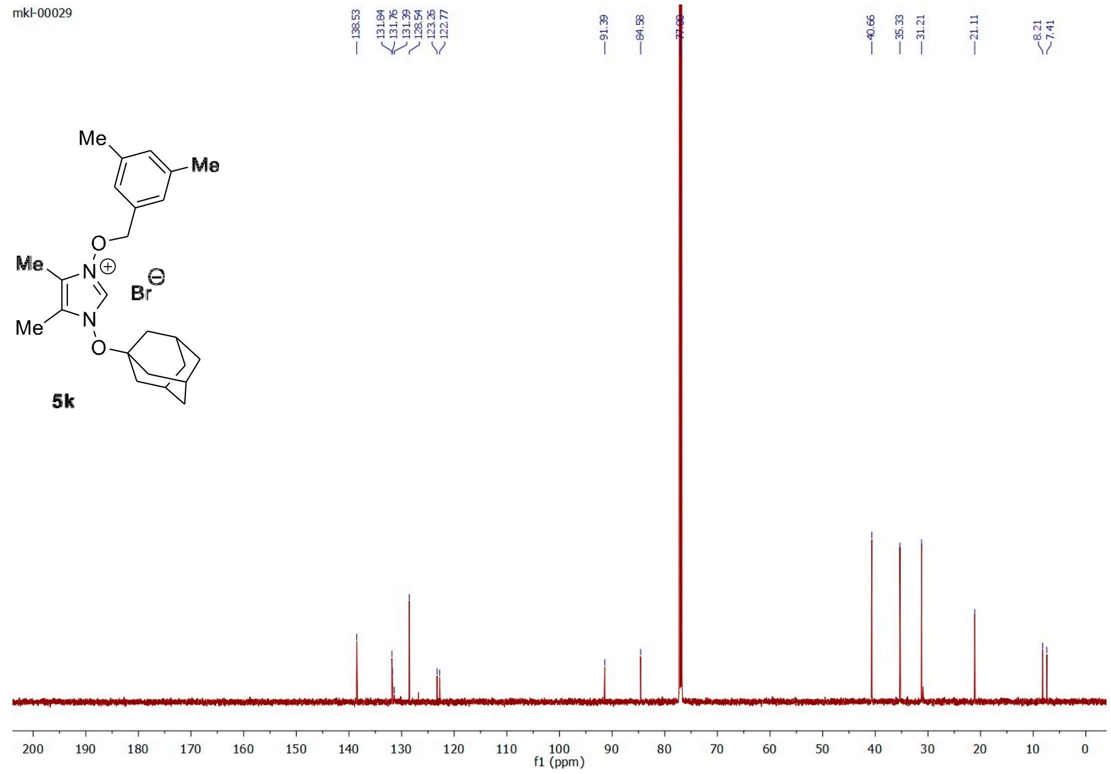

Figure S39. ^{13}C NMR of **5k** ($CDCl_3$, 151 MHz).

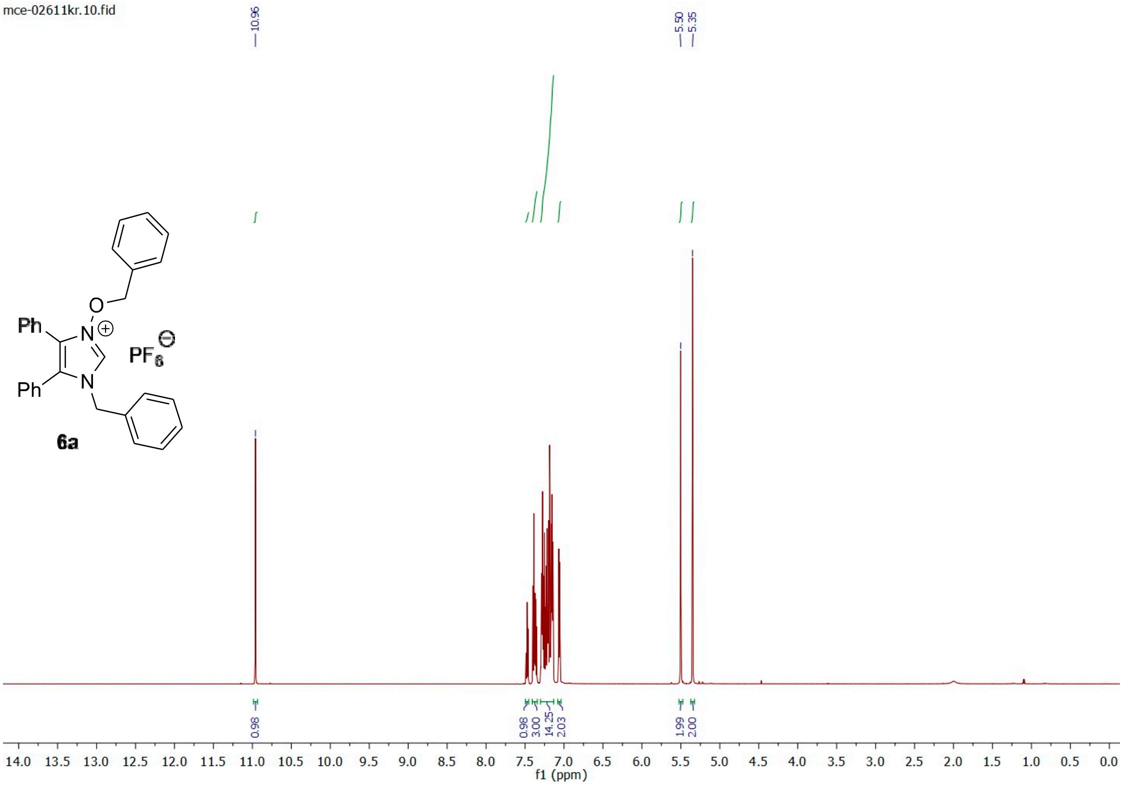

Figure S40. ^{1}H NMR of **6a** ($CDCl_3$, 600 MHz).

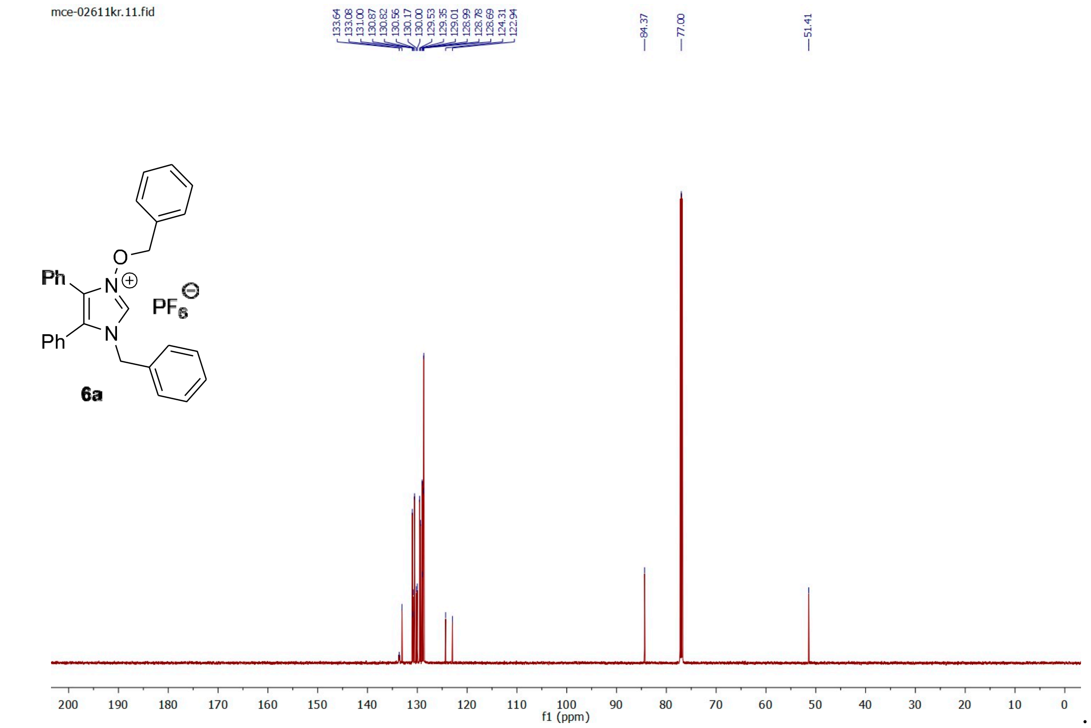

Figure S41. ^{13}C NMR of **6a** ($CDCl_3$, 151 MHz)..

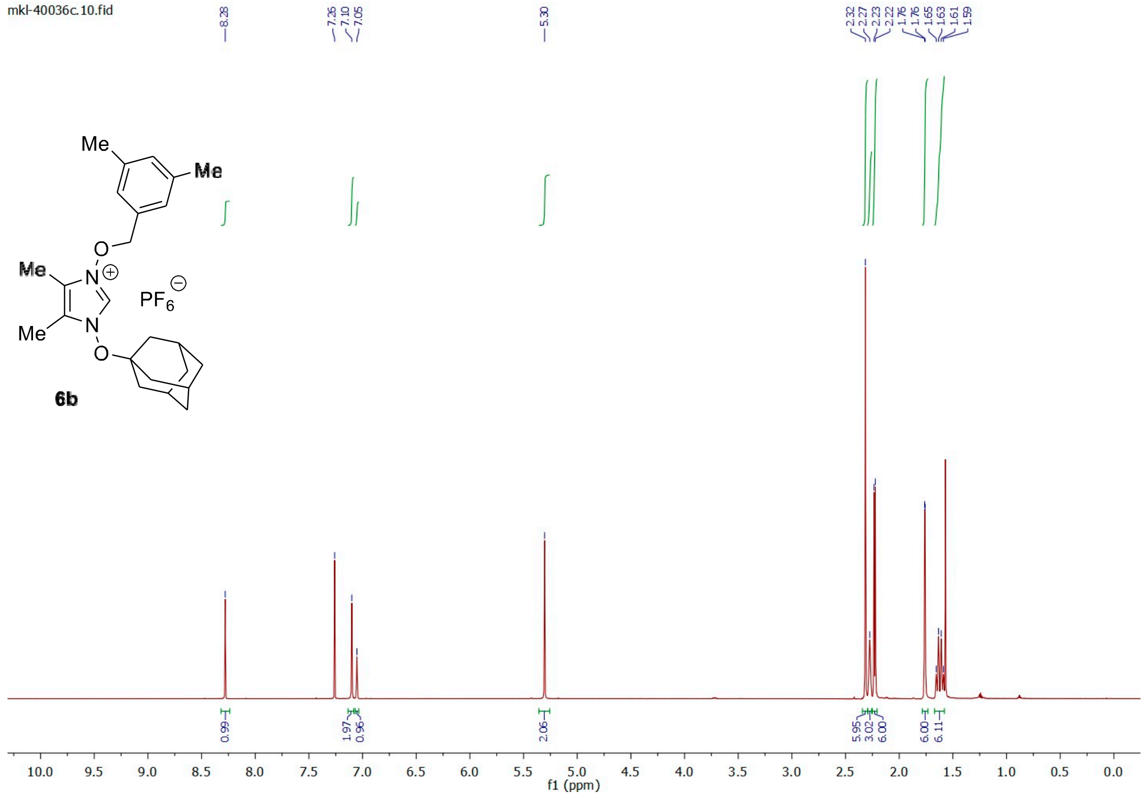

Figure S42. ^{1}H NMR of **6b** ($CDCl_3$, 600 MHz).

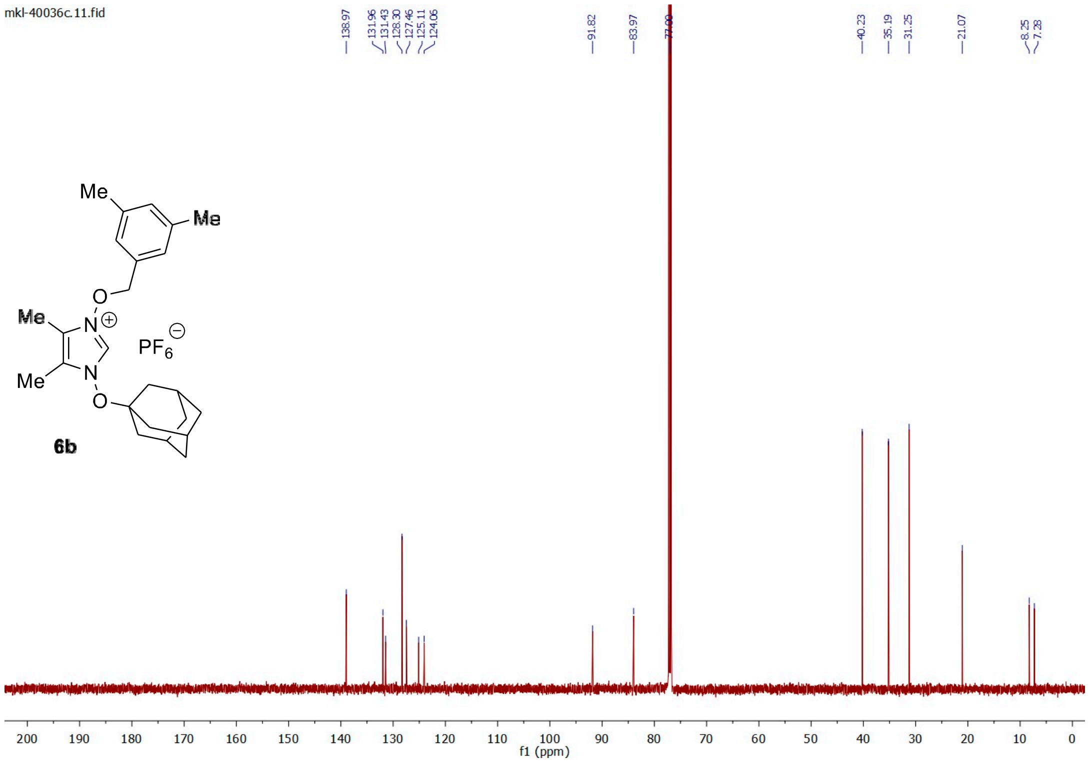

Figure S43. ^{13}C NMR of **6b** ($CDCl_3$, 151 MHz)..

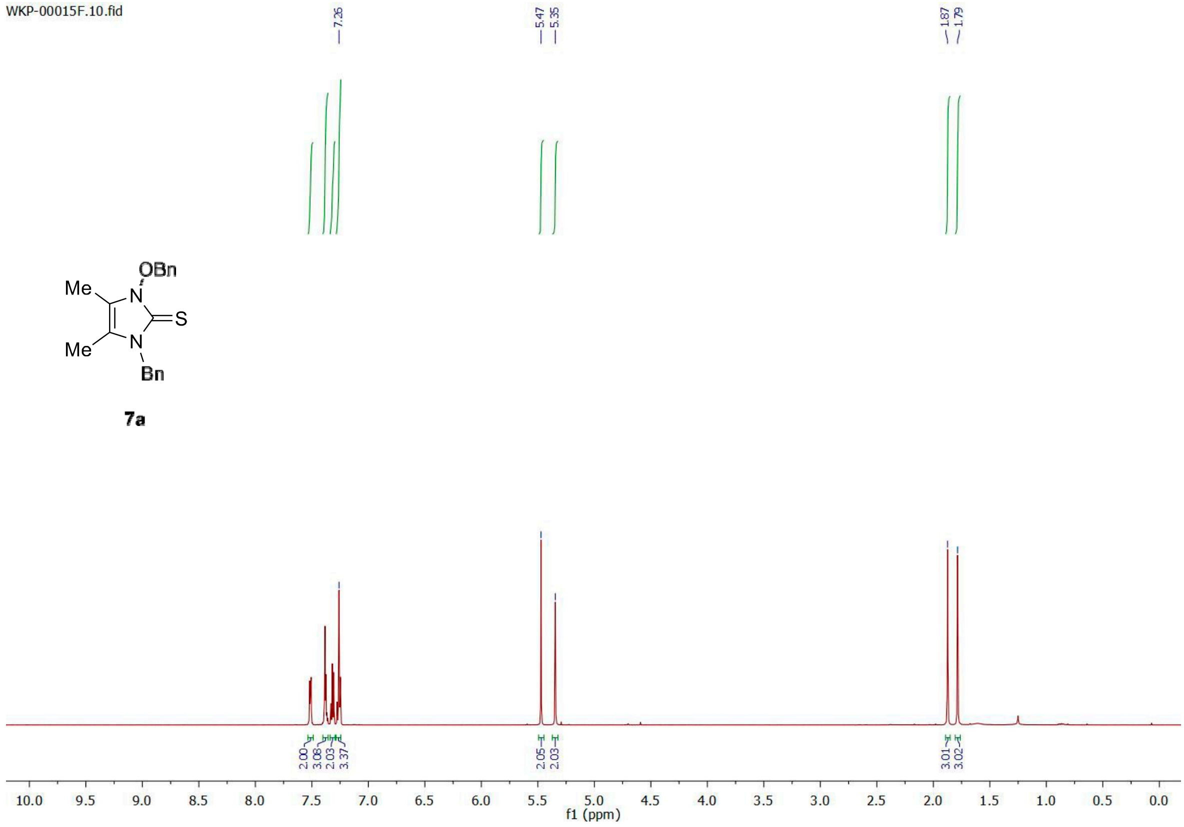

Figure S44. ^{1}H NMR of **7a** ($CDCl_3$, 600 MHz).

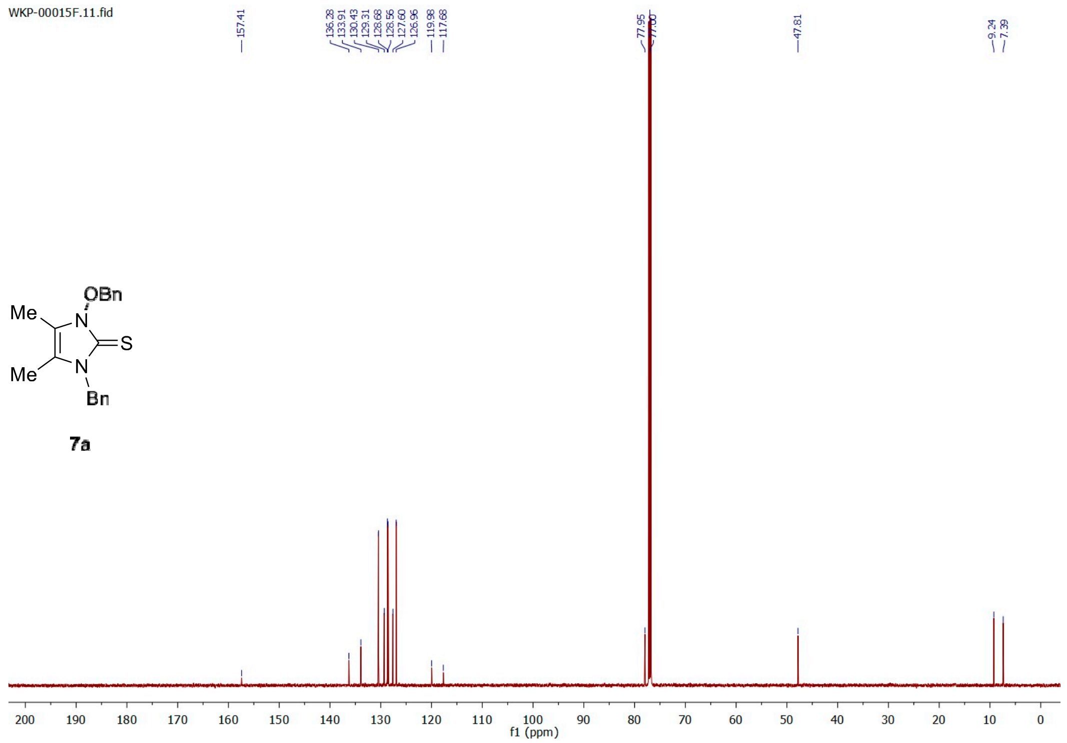

Figure S45. ^{13}C NMR of **7a** ($CDCl_3$, 151 MHz).

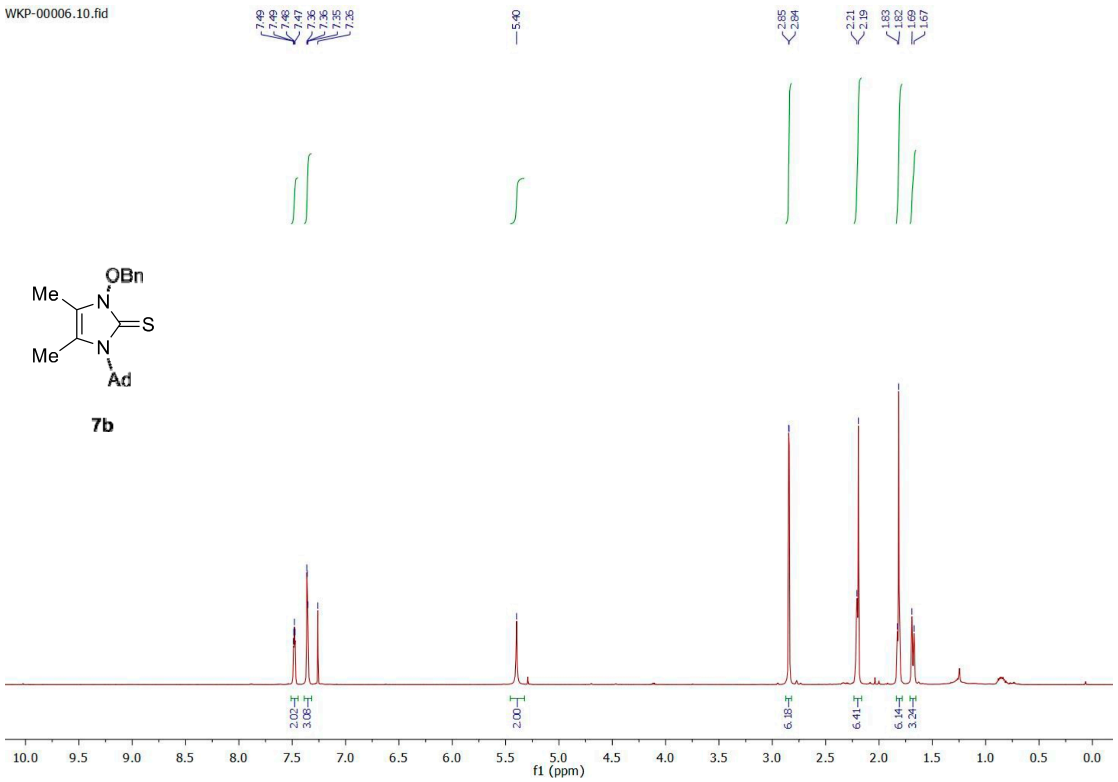

Figure S46. ^{1}H NMR of **7b** ($CDCl_3$, 600 MHz).

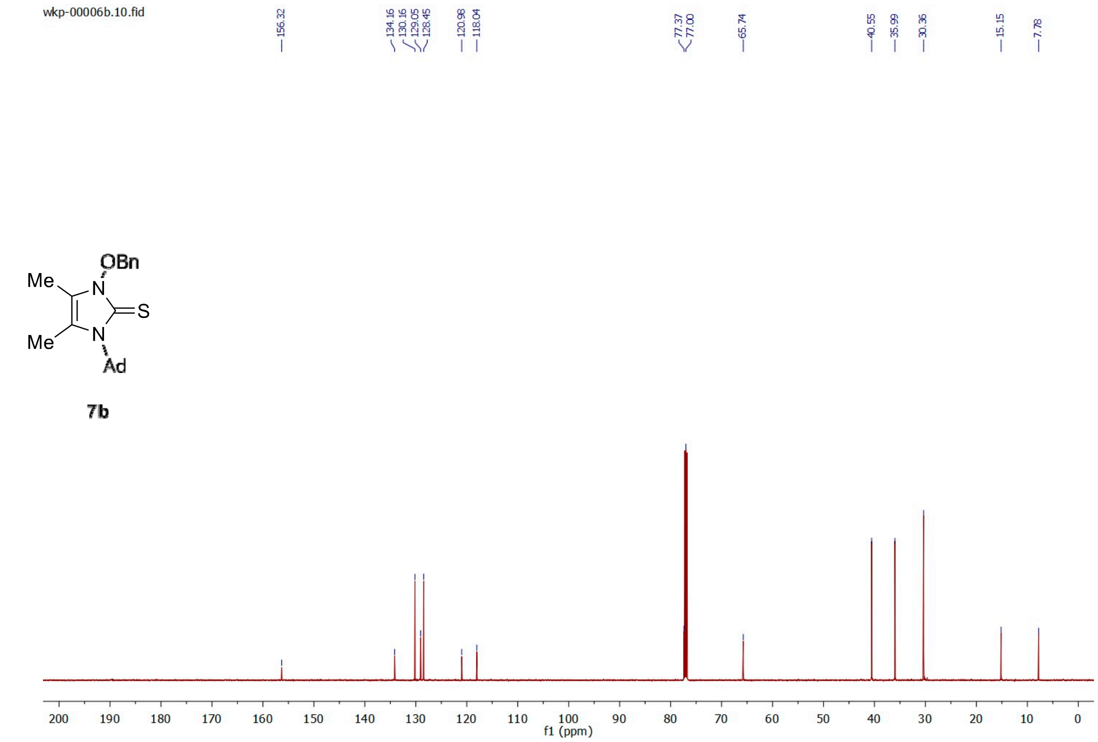

Figure S47. ^{13}C NMR of **7b** ($CDCl_3$, 151 MHz).

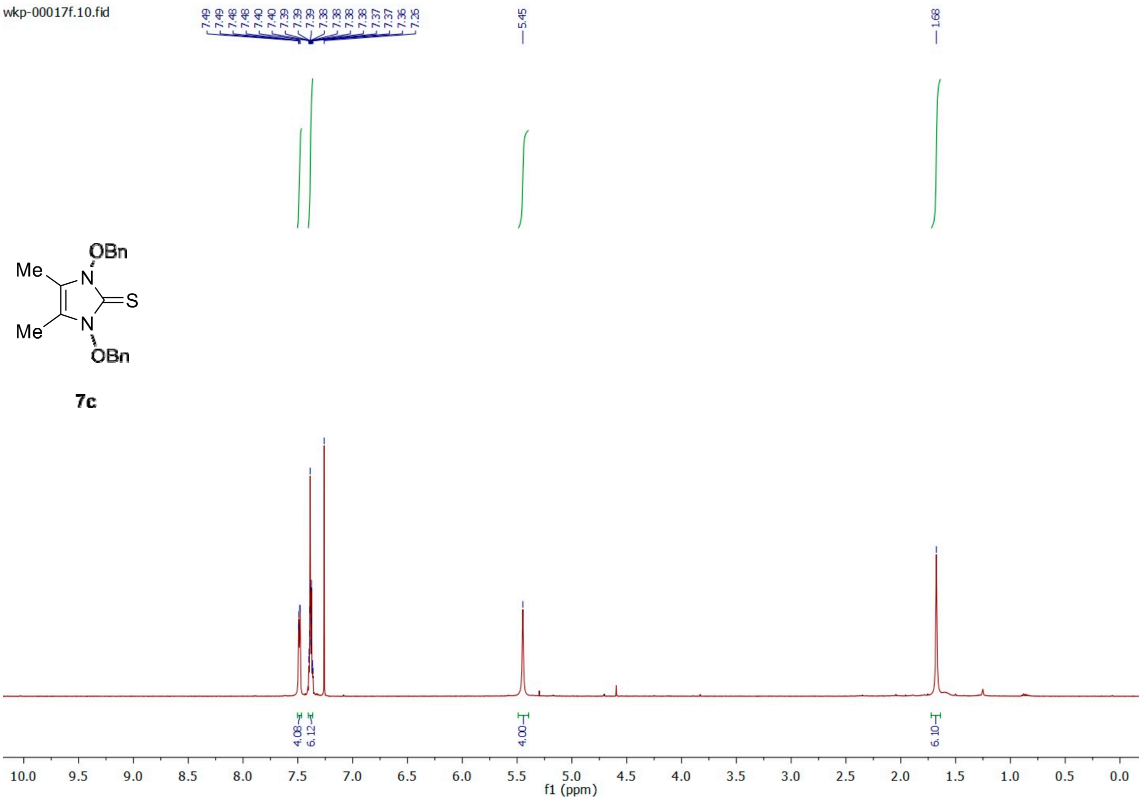

Figure S48. ^{1}H NMR of **7c** ($CDCl_3$, 600 MHz).

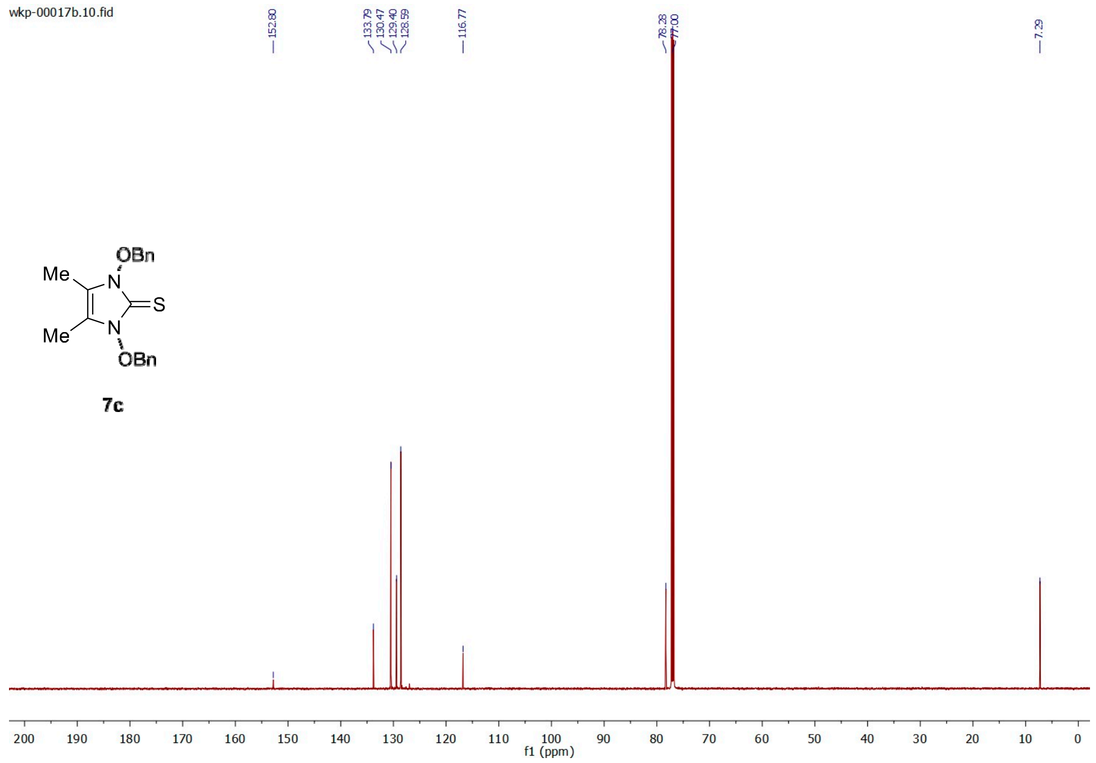

Figure S49. ^{13}C NMR of **7c** ($CDCl_3$, 151 MHz).

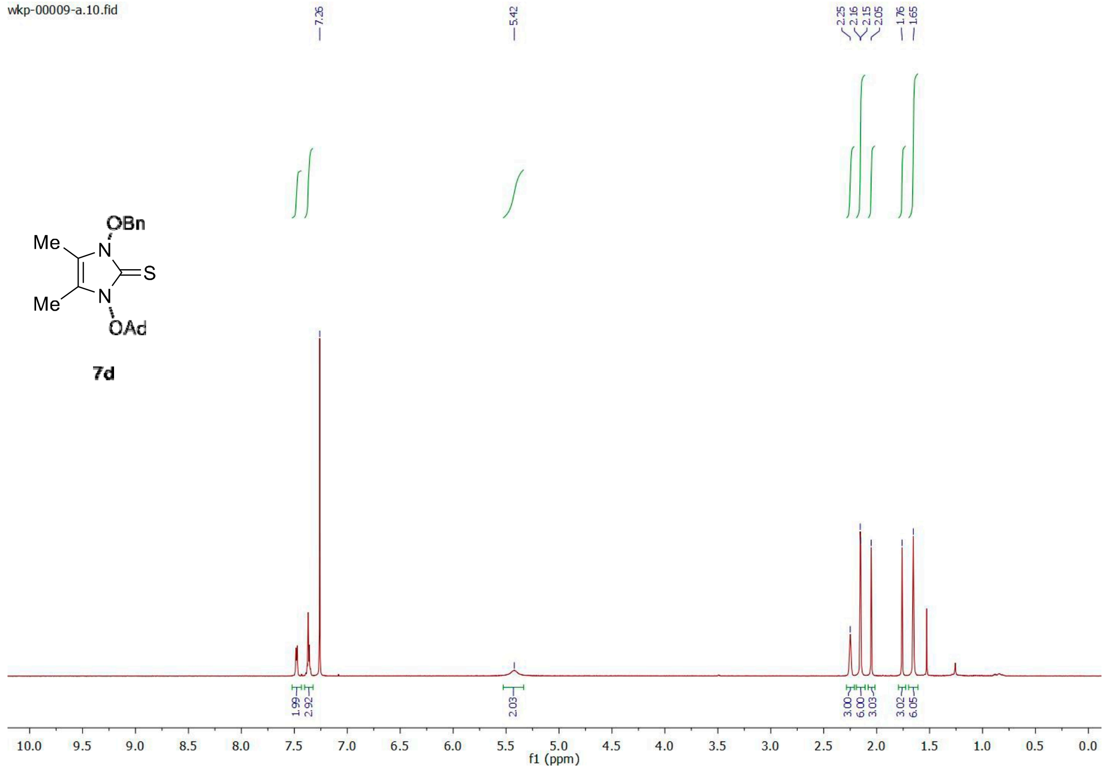

Figure S50. ^{1}H NMR of **7d** ($CDCl_3$, 600 MHz).

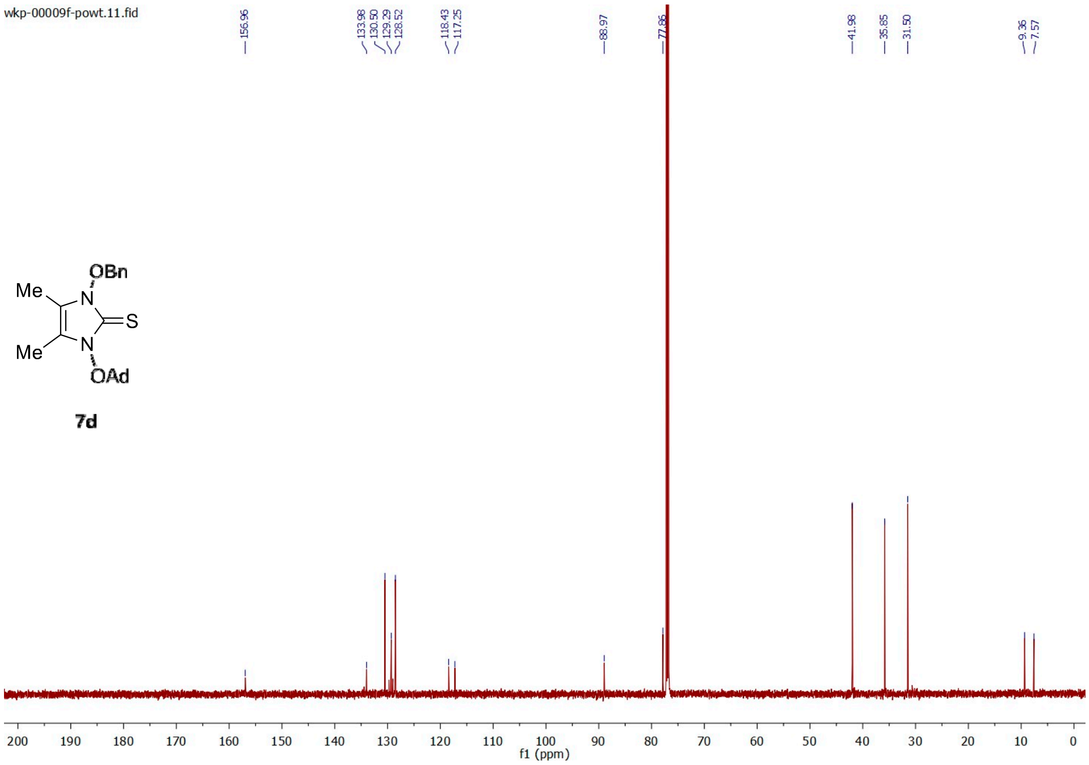

Figure S51. ^{13}C NMR of **7d** ($CDCl_3$, 151 MHz).

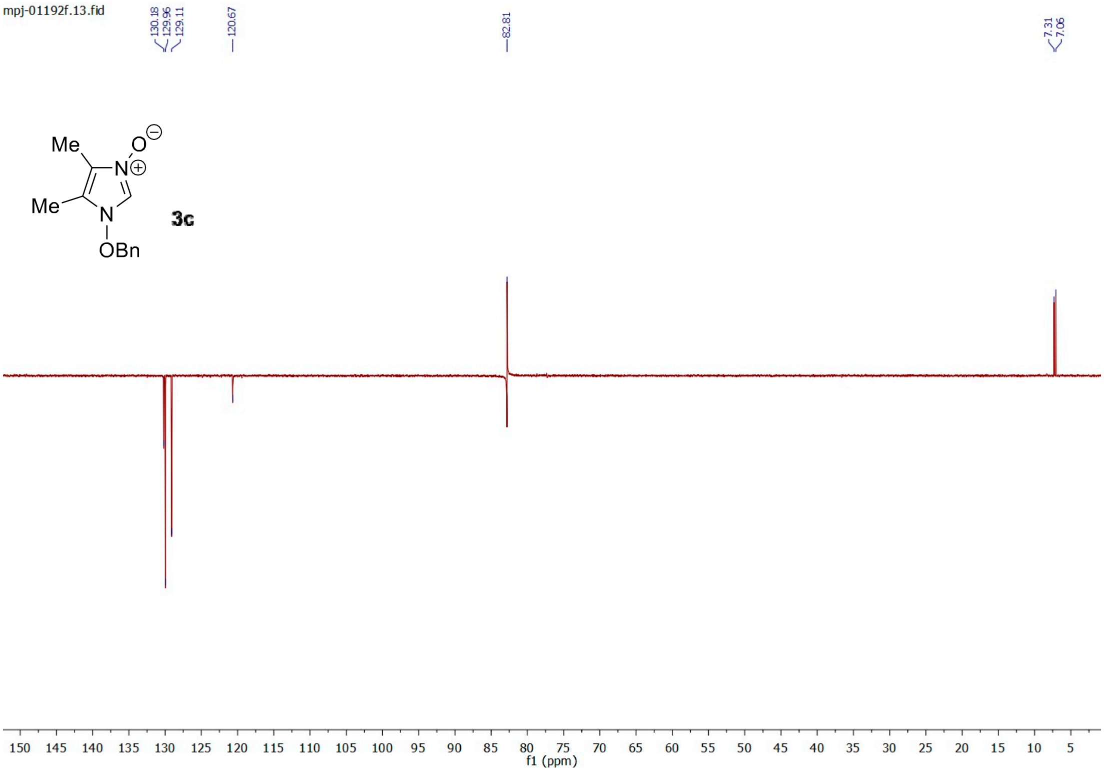

Figure S52. DEPT-135 of **3c** ($CDCl_3$).

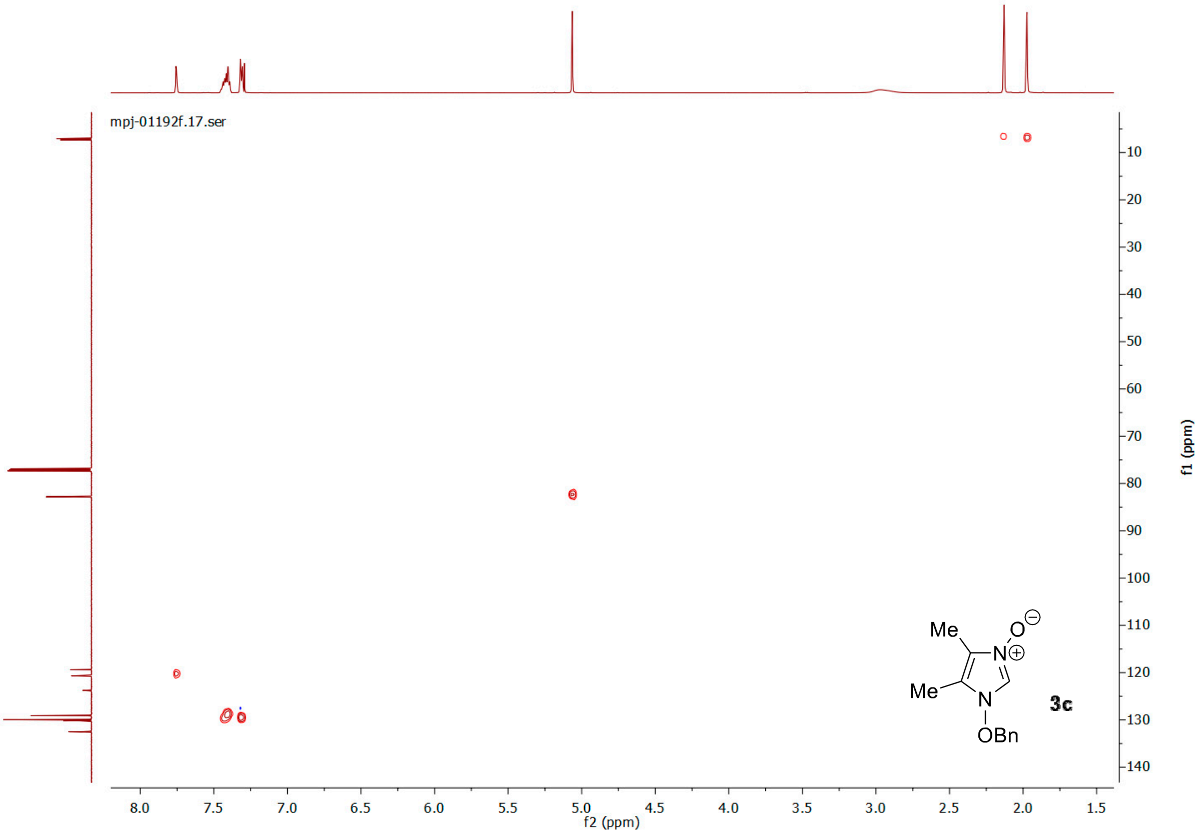

Figure S53. HMQC of **3c** ($CDCl_3$).

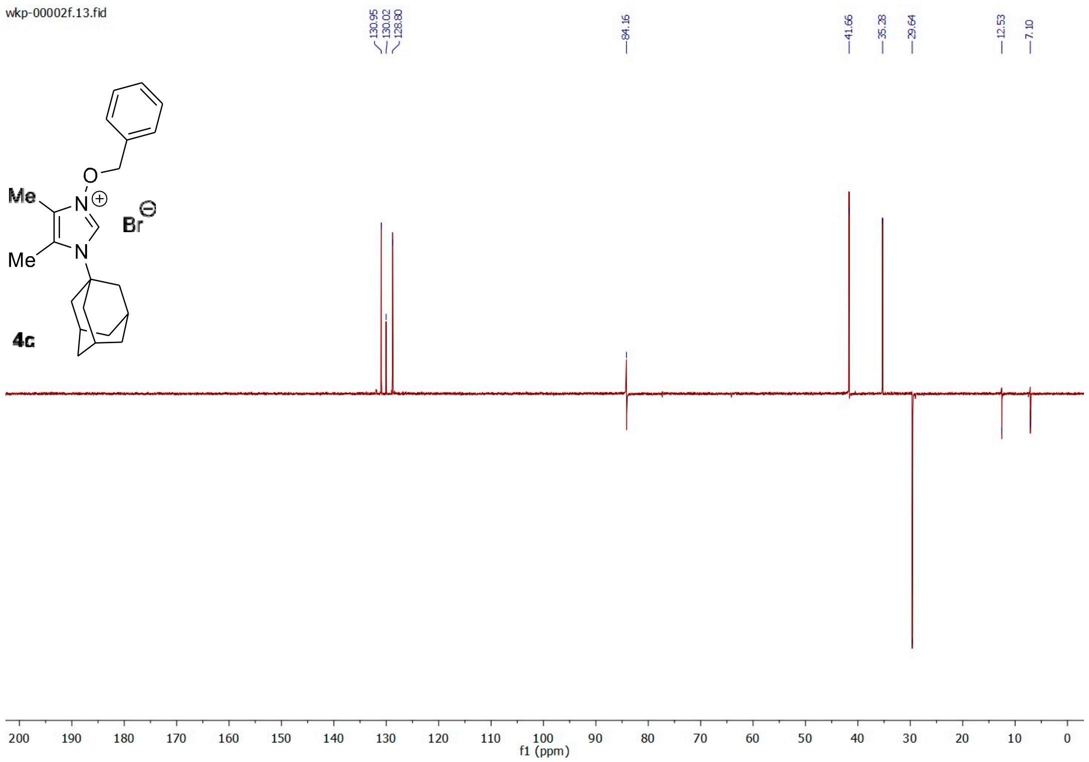

Figure S54. DEPT-135 of **4c** ($CDCl_3$).

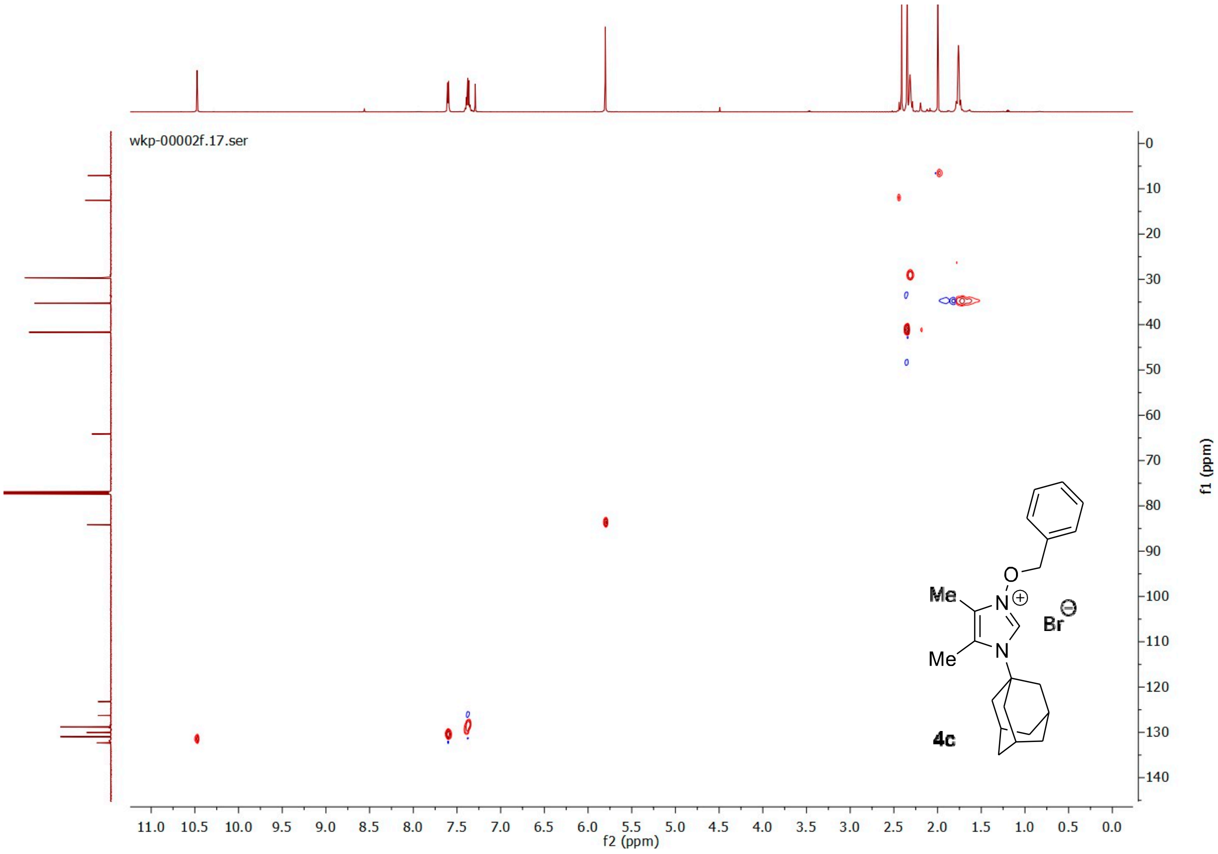

Figure S55. HMQC of **4c** ($CDCl_3$).

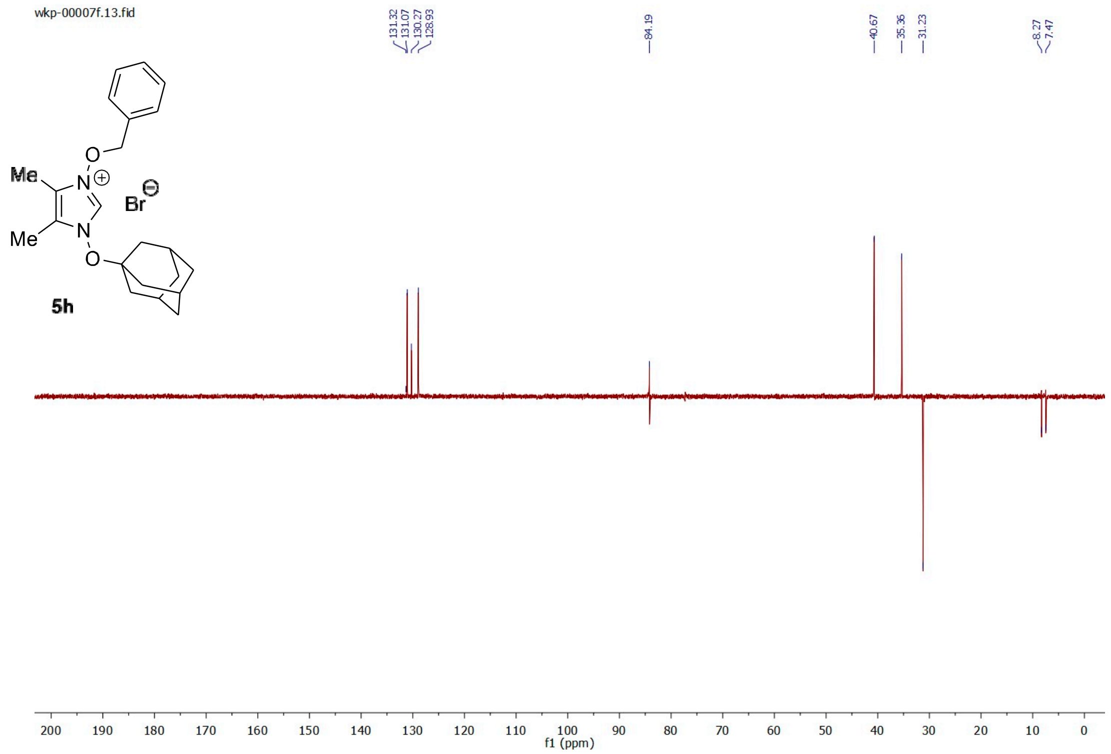

Figure S56. DEPT-135 of **5h** ($CDCl_3$).

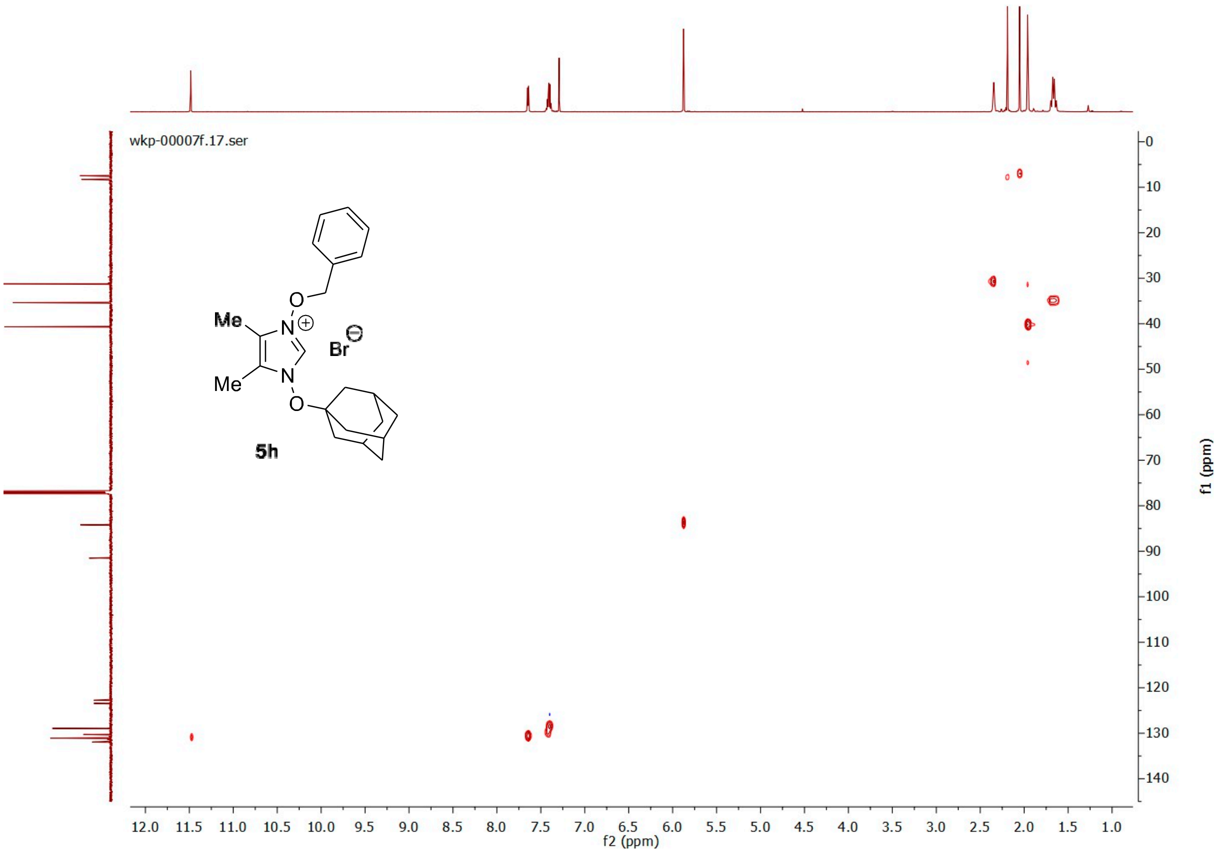

Figure S57. HMQC of **5h** (CDCl3).

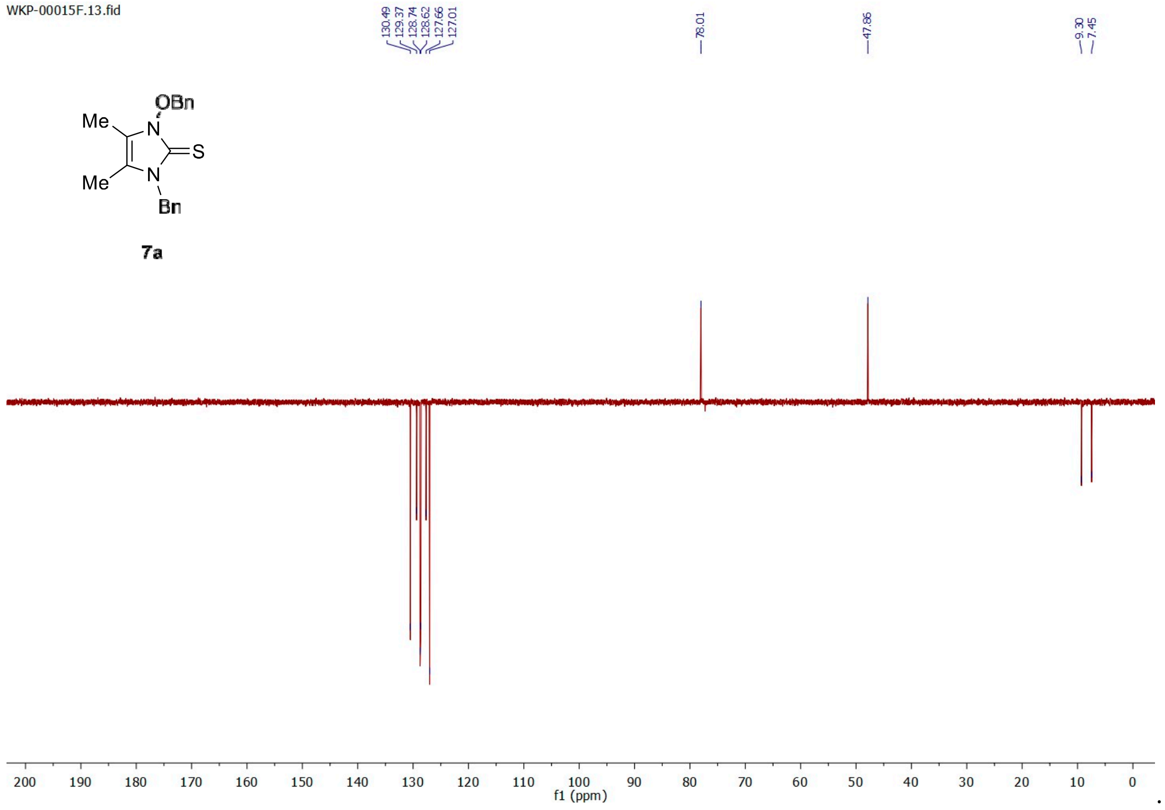

Figure S58. DEPT-135 of **7a** (CDCl3).

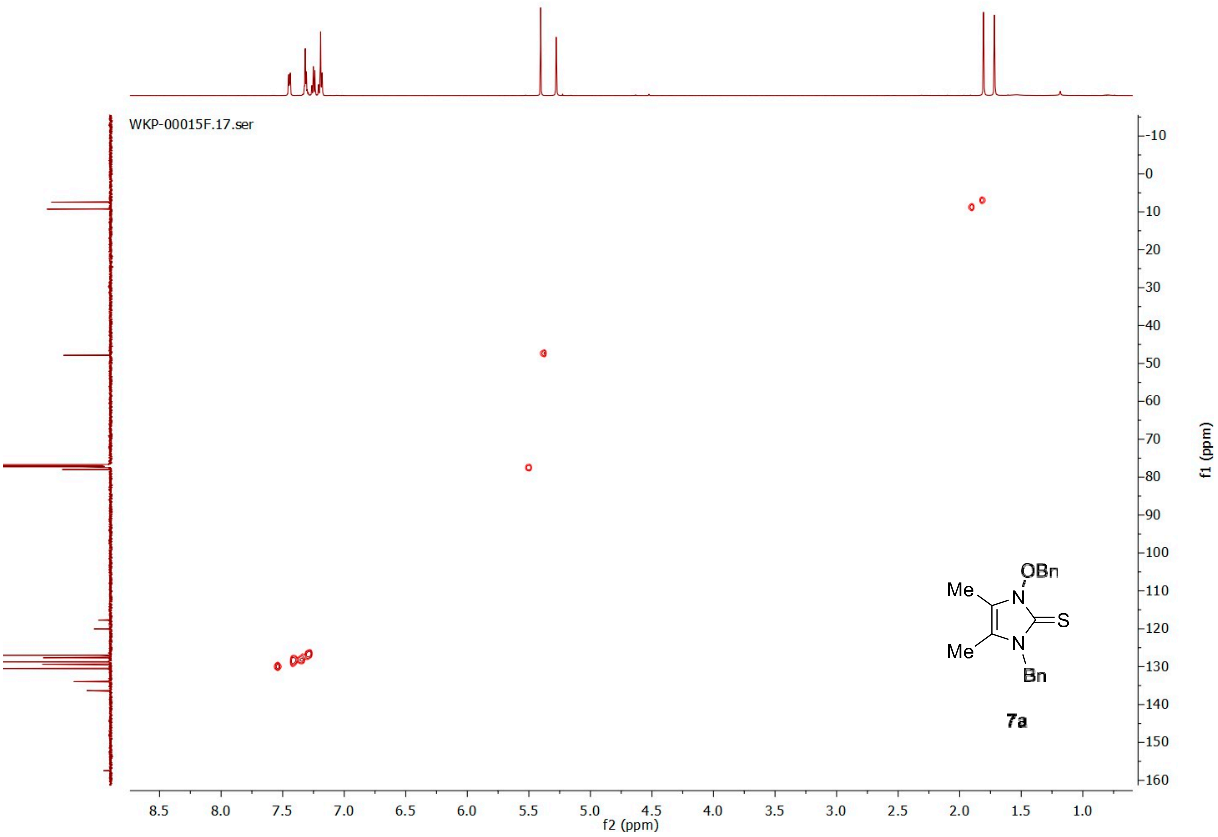

Figure S59. HMQC of **7a** ($CDCl_3$).
